# Assessment of Social Vulnerability in Pediatric Head and Neck Cancer Care and Prognosis in the United States

**DOI:** 10.1001/jamanetworkopen.2023.0016

**Published:** 2023-02-17

**Authors:** David J. Fei-Zhang, Daniel C. Chelius, Urjeet A. Patel, Stephanie S. Smith, Anthony M. Sheyn, Jeff C. Rastatter

**Affiliations:** 1 Northwestern University Feinberg School of Medicine, Chicago, Illinois; 2 Pediatric Thyroid Tumor Program and Pediatric Head and Neck Tumor Program, Department of Otolaryngology–Head and Neck Surgery, Texas Children’s Hospital, Baylor College of Medicine, Houston; 3 Department of Otolaryngology–Head and Neck Surgery, Northwestern University Feinberg School of Medicine, Chicago, Illinois; 4 Department of Pediatric Otolaryngology, Le Bonheur Children’s Hospital, Memphis, Tennessee; 5 Department of Otolaryngology–Head and Neck Surgery, University of Tennessee Health Science Center, Memphis; 6 Department of Pediatric Otolaryngology, St Jude Children’s Research Hospital, Memphis, Tennessee; 7 Division of Pediatric Otolaryngology, Ann & Robert H. Lurie Children’s Hospital of Chicago, Chicago, Illinois

## Abstract

**Importance:**

Prior investigations in social determinants of health (SDoH) in pediatric head and neck cancer (HNC) have only considered a narrow scope of HNCs, SDoH, and geography while lacking inquiry into the interrelational association of SDoH with disparities in clinical pediatric HNC.

**Objectives:**

To evaluate the association of SDoH with disparities in HNC among children and adolescents and to assess which specific aspects of SDoH are most associated with disparities in dynamic and regional sociodemographic contexts.

**Design, Setting, and Participants:**

This retrospective cohort study included data about patients (aged ≤19 years) with pediatric HNC who were diagnosed from 1975 to 2017 from the Surveillance, Epidemiology, and End Results Program (SEER) database. Data were analyzed from October 2021 to October 2022.

**Exposures:**

Overall social vulnerability and its subcomponent contributions from 15 SDoH variables, grouped into socioeconomic status (SES; poverty, unemployment, income level, and high school diploma status), minority and language status (ML; minoritized racial and ethnic group and proficiency with English), household composition (HH; household members aged ≥65 and ≤17 years, disability status, single-parent status), and housing and transportation (HT; multiunit structure, mobile homes, crowding, no vehicle, group quarters). These were ranked and scored across all US counties.

**Main Outcomes and Measures:**

Regression trends were performed in continuous measures of surveillance and survival period and in discrete measures of advanced staging and surgery receipt.

**Results:**

A total of 37 043 patients (20 729 [55.9%] aged 10-19 years; 18 603 [50.2%] male patients; 22 430 [60.6%] White patients) with 30 different HNCs in SEER had significant relative decreases in the surveillance period, ranging from 23.9% for malignant melanomas (mean [SD] duration, lowest vs highest vulnerability: 170 [128] months to 129 [88] months) to 41.9% for non-Hodgkin lymphomas (mean [SD] duration, lowest vs highest vulnerability: 216 [142] months vs 127 [94] months). SES followed by ML and HT vulnerabilities were associated with these overall trends per relative-difference magnitudes (eg, SES for ependymomas and choroid plexus tumors: mean [SD] duration, lowest vs highest vulnerability: 114 [113] months vs 86 [84] months; *P* < .001). Differences in mean survival time were observed with increasing social vulnerability, ranging from 11.3% for ependymomas and choroid plexus tumors (mean [SD] survival, lowest vs highest vulnerability: 46 [46] months to 41 [48] months; *P* = .43) to 61.4% for gliomas not otherwise specified (NOS) (mean [SD] survival, lowest vs highest vulnerability: 44 [84] months to 17 [28] months; *P* < .001), with ML vulnerability followed by SES, HH, and HT being significantly associated with decreased survival (eg, ML for gliomas NOS: mean [SD] survival, lowest vs highest vulnerability: 42 [84] months vs 19 [35] months; *P* < .001). Increased odds of advanced staging with non-Hodgkin lymphoma (OR, 1.21; 95% CI, 1.02-1.45) and retinoblastomas (OR, 1.31; 95% CI, 1.14-1.50) and decreased odds of surgery receipt for melanomas (OR, 0.79; 95% CI, 0.69-0.91) and rhabdomyosarcomas (OR, 0.90; 95% CI, 0.83-0.98) were associated with increasing overall social vulnerability.

**Conclusions and Relevance:**

In this cohort study of patients with pediatric HNC, significant decreases in receipt of care and survival time were observed with increasing SDoH vulnerability.

## Key Points

**Question:**

Are social determinants of health associated with pediatric head and neck cancer (HNC) disparities in clinical contexts across the United States?

**Findings:**

This cohort study of 37 043 patients with pediatric HNC observed statistically significant decreases in the surveillance period as large as 41.2% and in the survival period as large as 61.4% as overall social vulnerability increased. Socioeconomic status, race and ethnicity, proficiency with English, household composition, and housing and transportation vulnerabilities were differentially associated with these overall trends.

**Meaning:**

These findings could help to inform health care professionals about which social determinants contribute to pediatric HNC disparities, setting foundations toward initiating specific investigations and policies.

## Teaser

This cohort study evaluates the association of overall social vulnerability and specific social determinants of health with disparities in head and neck cancer care and prognosis among children and adolescents in the United States.

## Introduction

Social determinants of health (SDoH) crucially contribute to pediatric cancer disparities.^1,2,3,4^ SDoH are especially important in head and neck cancers (HNCs), which comprise the leading causes of child and adolescent mortality by disease in the United States.^5^ Most literature on SDoH in HNC focuses on the adult population, with marked disparities noted for groups differing by patient sex, race and ethnicity, substance use, and other socioeconomic determinants.^6,7,8^ From the limited SDoH studies within pediatric HNC, Gruszczynski et al^9^ observed that male sex, minoritized race, poverty, and language isolation contributed to worse overall survival among 3919 patients with pediatric thyroid cancer across the United States from 1973 to 2015. Marcotte et al^1^ assessed the association of race and ethnicity with a broad range of pediatric cancers and found that patients who belonged to minorized racial and ethnic groups experienced significantly lower incidence among several tumor types. Despite these prior studies of SDoH and HNC, they did not principally focus on pediatric populations and did not include all HNC types. Furthermore, to our knowledge, no prior studies have examined a wider range of SDoH within pediatric HNC or how multiple SDoH come together to affect pediatric HNC disparities in a nationwide clinical context.

The US Centers for Disease Control and Prevention Social Vulnerability Index (CDC-SVI) is a recently updated, US Census–based tool featuring 15 social factors grouped into 4 SDoH-related themes that are relatively ranked across all US Census tracts and counties, providing a wide yet in-depth index for evaluating SDoH (eFigure 1 in Supplement 1).^10^ Although intended for gauging health resources during natural disasters, the CDC-SVI has been used to investigate how social vulnerabilities affect specific diseases, namely COVID-19.^11,12,13,14^ Its utility has yet to be fully explored in cancer, especially pediatric HNC, as prior studies using CDC-SVI have only examined disparities of care settings and postoperative outcomes for lung and gastrointestinal pathologies.^15,16,17^ Thus, our study aimed to use the CDC-SVI and its themes of socioeconomic status (SES), minority and language status (ML), household composition (HH), and housing and transportation (HT) to explore the associations of SDoH with care and prognostic outcomes among children and adolescents with HNC across the United States.

## Methods

This retrospective cohort study follows the Strengthening the Reporting of Observational Studies in Epidemiology (STROBE) reporting guideline. No prior institutional review board or ethics committee approval or waiver of informed consent was needed per Northwestern University’s institutional policy because the databases queried consist of publicly available deidentified data.

### Databases

The CDC-SVI was queried for ranked scores among 15 Census factors within 4 SDoH themes of SES (poverty, unemployment, income level, and high school diploma status), ML (minoritized racial and ethnic group [American Indian and Alaska Native, Asian, Black or African American, American Indian and Alaska Native, Asian, Native Hawaiian or other Pacific Islander, other, and multiracial (per the US Census) and proficiency with English), HH (household members aged ≥65 and ≤17 years, disability status, single-parent status), and HT (multiunit structure, mobile homes, crowding, no vehicle, group quarters) as well as total composite scores. Based on CDC-SVI documentation,^10^ theme subscores are differentially weighed to formulate the total composite score and are assigned different weights based on the designated area’s sociodemographic and Census data. Total and theme scores are based on relative social vulnerabilities of a particular census tract among all 72 158 US Census tracts, ranging from 0 to 1, with 0 representing the lowest social vulnerability and 1 representing the highest.

The National Cancer Institute Surveillance, Epidemiology, and End Results Program (SEER) database contains national data sets of patient variables, pathological characteristics, treatment modalities, and prognostic outcomes. Race and ethnicity (American Indian or Alaska Native, Asian and Pacific Islander, Black, Hispanic, White, and unknown) are collected and were used to determine minority status. Months under surveillance is a length-of-care measurement reflecting the active follow-up a patient receives for their primary malignant neoplasm until the last interaction with a health care professional. Months’ survival represents active follow-up until the patient dies. Staging is based on SEER-designated variables labeled as, eg, stage 4, distant (expansion), or distal (expansion), and recoded under *American Joint Committee on Cancer*, 6th Edition (AJCC-6) classifications. Primary surgery occurrence indicates whether patients received surgery for their primary malignant neoplasm.

SVI scores were abstracted and matched to patients in the SEER database on county of residence at the time of diagnosis. County-assigned scores were generated by weighted score means per population density of each census tract within the county. The schematic workflow is provided in eFigure 2 in Supplement 1.

### Population Definitions

SEER was queried for pediatric patients (≤19 years) diagnosed with head and neck malignant neoplasms from 1975 to 2017. Head and neck sites were extracted using the *International Classification of Diseases for Oncology, Third Edition* (*ICD-O-3*) topographic codes: C00.0 to C15.0; C15.3; C30.0 to C34.0; C37.9; C39.0 to C40.1; C41.0 to C41.2; C42.3; C44.0 to C44.8; C47.0 to C47.1; C49.0 to C49.1; C69.0 to C72.0; C72.2 to C72.8; C73.9; C75.0 to C75.3; C76.0; C76.4; and C77.0. Specific diseases were categorized by the International Classification of Childhood Cancers (ICCC).

### Statistical Analysis

Months surveyed or followed up within each disease class were analyzed by total CDC-SVI score and theme subscores. CDC-SVI scores were split into relative equivalently sampled quintiles based on actual scores within each disease class. The relative SVI quintiles were delineated by less than 20, 20 to 39.99, 40 to 59.99, 60 to 79.99, and 80 to 99.99, representing their relative percentiles per disease class (eg, within disease A, patients with the lowest CDC-SVI scores are grouped into the <20 quintile group).

Among these total and theme quintiles, differences between the mean months’ surveyed for lowest and highest quintiles were calculated. Trend significance was assessed by linear regression across all data points against relative SVI quintiles for months’ surveyed (ie, not a trend through the baseline descriptive values), and violin plots were generated to assess relative sample distributions for months’ surveyed along the contour widths within each relative SVI quintile while simultaneously measuring the median, IQR, and 1.5 × IQR with its inner boxplot. Means, SDs, and ranges for months’ surveyed per quintile were also calculated. The proportion of patients who were alive or lost to follow-up vs dead was calculated per quintile. Means, SDs, and ranges for actual total CDC-SVI scores and theme subscores were calculated per relative quintile group. A representative figure of this analysis for a data subset can be seen in eFigure 3 in Supplement 1.

Survival months within respective ICCC disease classes were analyzed similarly as months surveyed. However, after separating patients into relative SVI quintiles within each respective disease class, patients who were alive or lost on last follow-up were excluded to extract patients who died. Primary surgery occurrence and advanced staging on time of diagnosis within disease classes were analyzed with univariate logistic regression across relative SVI quintiles per CDC-SVI category.

Statistical significance was set as *P* < .05. Two-sided *P* values are reported for analyses. Analyses were conducted in R version 4.2.1 (R Project for Statistical Computing).

## Results

Of the 37 231 pediatric patients (≤19 years) with primary malignant neoplasms located in sites of the head and neck that were identified in SEER, 37 043 patients were included in the analytic cohort based on presence of necessary analytical variables. Overall, 20 729 participants (55.9%) were aged 10 to 19 years, 18 603 (50.2%) were male patients, and 22 430 (60.6%) were White patients. Total CDC-SVI scores ranged from 0.000 to 0.947. SES subscores ranged from 0.00 to 0.976. ML subscores ranged from 0.002 to 0.945. HH subscores ranged from 0.091 to 0.971. HT subscores ranged from 0.051 to 0.894. Patient clinical characteristics stratified by total CDC-SVI and theme subscores appear in eTable 1 in Supplement 1.

### ICCC Disease-Specific Trends in Months Under Surveillance by Relative CDC-SVI Percentile

**Figure 1.**
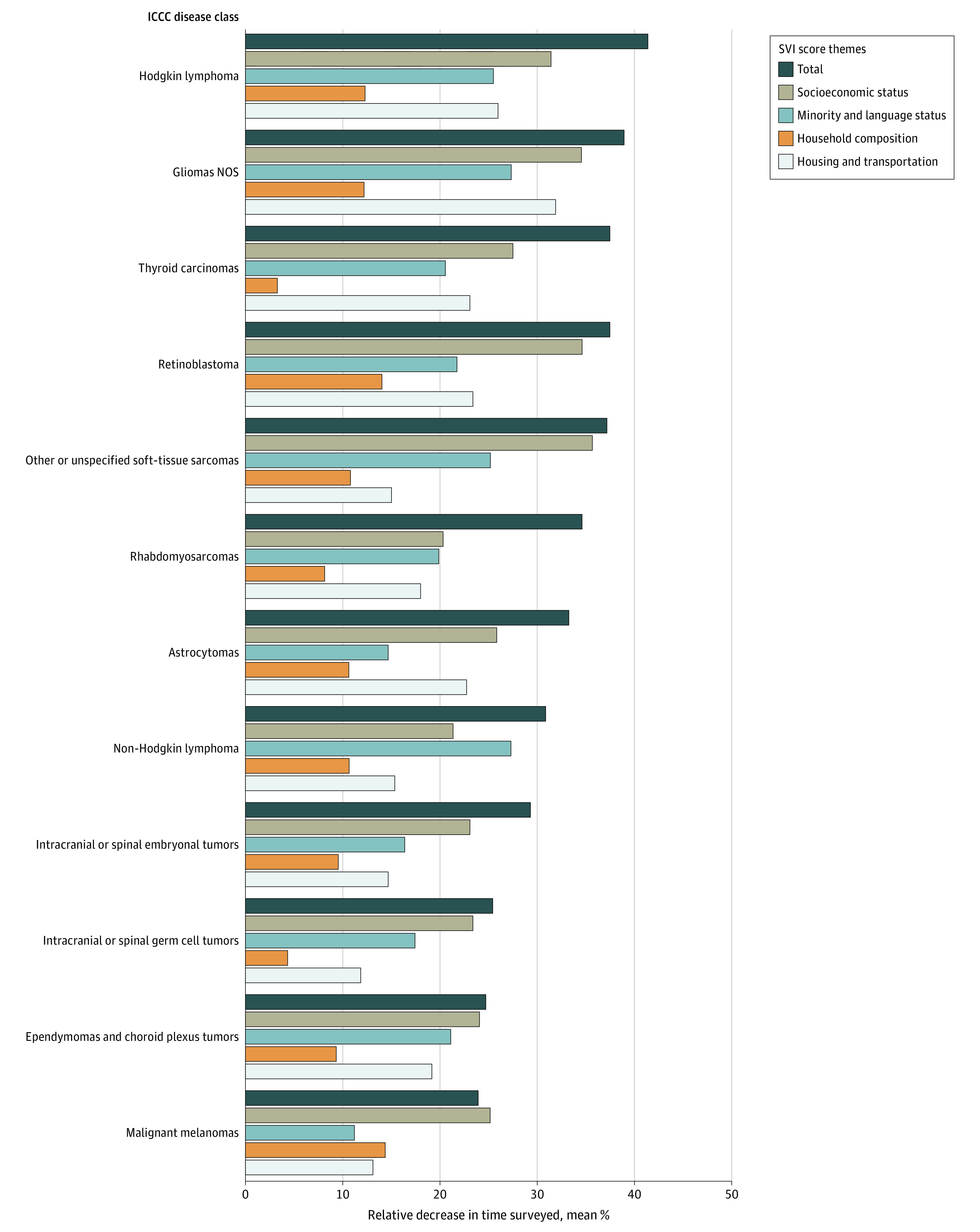
Association of Increasing Social Vulnerability Index (SVI) Scores With Months’ Surveyed, by International Classification of Childhood Cancers (ICCC) Disease Class Bars represent the percentage decreases from lowest to highest SVI quintiles based on mean months’ surveyed for total SVI score and subcomponent SVI theme subscores per class. Socioeconomic status included poverty, unemployment, income level, and high school diploma status; minority and language status, minoritized racial and ethnic group and proficiency with English, household composition, household members aged 65 years and older and 17 years and younger, disability status, single-parent status; and housing and transportation, multiunit structure, mobile homes, crowding, no vehicle, group quarters. NOS indicates not otherwise specified.

Across ICCC disease classes, substantial relative decreases in mean months’ surveyed were observed among patients from the lowest (ie, least socially vulnerable overall) to the highest total SVI quintiles, with differences ranging from as high as 41.2% for Hodgkin lymphomas (mean [SD] duration, 216 [142] months vs 127 [94] months) to as low as 23.9% for malignant melanomas (mean [SD] duration, 170 [128] months to 129 [88] months) (Figure 1 and eTable 2 in Supplement 1). These decreases were statistically significant for astrocytomas, ependymomas and choroid plexus tumors, intracranial or spinal embryonal tumors, intracranial or spinal germ cell tumors, Hodgkin lymphoma, non-Hodgkin lymphoma, malignant melanomas, retinoblastomas, rhabdomyosarcomas, thyroid carcinomas, gliomas not otherwise specified (NOS), and other or unspecified soft tissue sarcomas (eFigure 4 and eTable 2 in Supplement 1).

Contributing to these total SVI and overall vulnerability trends, increasing theme subscores (ie, vulnerability in a specific SDoH theme) were associated with decreases in months’ surveyed. Increasing SES and ML vulnerabilities were associated with significant decreases across all classes (eg, SES for ependymomas and choroid plexus tumors: mean [SD] duration, lowest vs highest vulnerability: 114 [113] months vs 86 [84] months; *P* < .001). Increasing vulnerability with HH was associated with significant decreases across all classes except for ependymomas and choroid plexus tumors, intracranial or spinal germ cell tumors, non-Hodgkin lymphomas, and rhabdomyosarcomas (eg, astrocytomas: mean [SD] duration, lowest vs highest vulnerability: 131 [120] months vs 117 [115] months; *P* < .001). Increasing HT vulnerability was associated with significant decreases across all classes except intracranial or spinal germ cell tumors (eg, Hodgkin lymphoma: mean [SD] duration, lowest vs highest vulnerability: 197 [143] months vs 146 [104] months; *P* < .001) (eFigure 4 and eTable 2 in Supplement 1).

### ICCC Disease-Specific Trends in Survival Months by Relative CDC-SVI Percentile

**Figure 2.**
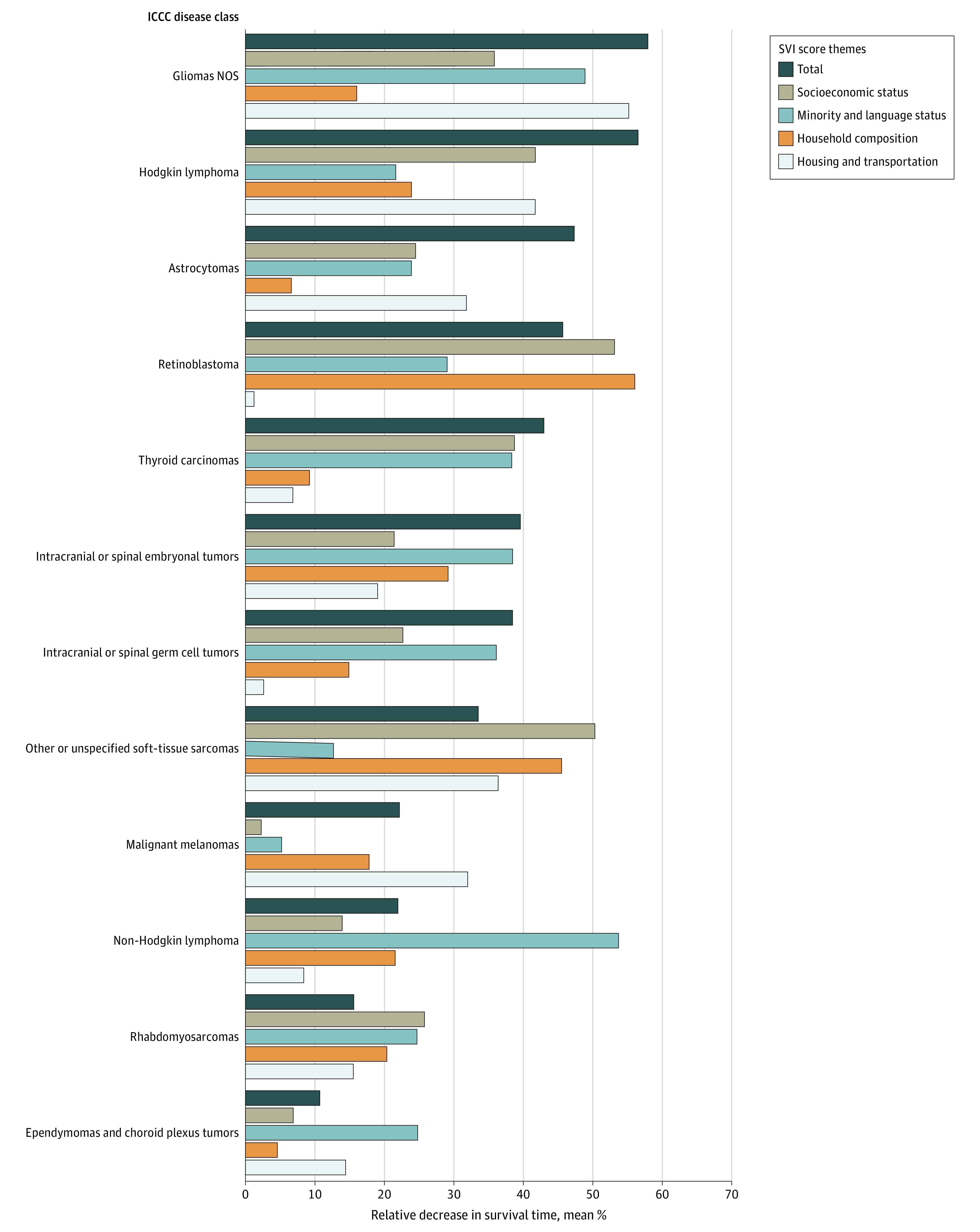
Association of Increasing Social Vulnerability Index (SVI) Scores With Months’ Survival, by International Classification of Childhood Cancers (ICCC) Disease Class Bars represent the percentage decreases from lowest to highest SVI quintiles based on mean months’ survived for total SVI score and subcomponent SVI theme subscores per class. Socioeconomic status included poverty, unemployment, income level, and high school diploma status; minority and language status, minoritized racial and ethnic group and proficiency with English, household composition, household members aged 65 years and older and 17 years and younger, disability status, single-parent status; and housing and transportation, multiunit structure, mobile homes, crowding, no vehicle, group quarters. NOS indicates not otherwise specified.

For ICCC disease classes, differences in mean months’ survival between the lowest to highest total SVI quintiles ranged from as high as a 61.4% decrease for gliomas NOS (mean [SD] survival, 44 [84] months to 17 [28] months; *P* < .001) to as low as an 11.3% decrease for ependymomas and choroid plexus tumors (mean [SD] survival, 46 [46] months to 41 [48] months) (Figure 2 and eTable 3 in Supplement 1). These decreases in months’ survived were statistically significant for astrocytomas (mean [SD] survival, lowest vs highest vulnerability: 48 [79] months vs 27 [42] months; *P* < .001), intracranial or spinal embryonal tumors (mean [SD] survival, lowest vs highest vulnerability: 0.31 [0.05] months vs 0.69 [0.07] months; *P* < .001), Hodgkin lymphoma (mean [SD] survival, lowest vs highest vulnerability: 252 [143] months vs 113 [133] months; *P* < .001), thyroid carcinomas (mean [SD] survival, lowest vs highest vulnerability: 218 [149] months vs 122 [114] months; *P* = .03), and gliomas NOS (mean [SD] survival, lowest vs highest vulnerability: 44 [84] months vs 17 [28] months; *P* < .001) (eFigure 5 and eTable 3 in Supplement 1).

Contributing to these total CDC-SVI vulnerability trends, increasing subscore vulnerability in specific themes showed differential associations with decreased survival across the ICCC classes. Increasing vulnerability in SES was associated with significant decreases in survival months for all prior-mentioned classes (eg, astrocytomas: mean [SD] survival, lowest vs highest vulnerability: 58 [93] months vs 42 [78] months; *P* < .001). Increasing vulnerability in ML status was associated with significant decreases in survival across ICCC classes except Hodgkin lymphoma and thyroid carcinomas (eg, gliomas NOS: mean [SD] survival, lowest vs highest vulnerability: 42 [84] months vs 19 [35] months; *P* < .001). Increasing vulnerability in HH was associated with decreased survival across ICCC classes except with astrocytomas, gliomas NOS, and thyroid carcinomas (eg, Hodgkin lymphoma: mean [SD] survival, lowest vs highest vulnerability: 206 [152] months vs 162 [137] months; *P* = .03). Increasing vulnerability in HT was associated with decreases across ICCC classes except for thyroid carcinomas (eg, Hodgkin lymphoma: mean [SD] survival, lowest vs highest vulnerability: 236 [149] months vs 137 [102] months; *P* = .01) (eFigure 5 and eTable 3 in Supplement 1).

### ICCC Disease-Specific Analyses of Advanced Staging on Presentation by Relative CDC-SVI Percentile

For ICCC disease classes, increasing total CDC-SVI vulnerability was associated with increased odds of advanced stage on preliminary presentation for those with non-Hodgkin lymphoma (odds ratio [OR], 1.21; 95% CI, 1.02-1.45; *P* = .03) and retinoblastomas (OR, 1.31; 95% CI, 1.14-1.50; *P* < .001). For non-Hodgkin lymphoma, only increasing vulnerability in SES was associated with increased odds. For retinoblastomas, increasing SES, ML, and HT vulnerability were associated with increased odds of advanced stage on presentation (Table 1).

**Table 1.** Association Between Advanced Staging on Preliminary Presentation and Increasing Social Vulnerability Index Score^a^

ICCC class	OR (95% CI)	*P* value
Astrocytomas		
Overall	1.09 (0.97-1.22)	.14
Socioeconomic status	1.14 (1.02-1.29)	.03
Minority and language status	1.01 (0.90-1.14)	.84
Household composition	1.16 (1.04-1.31)	.01
Housing and transportation	1.03 (0.92-1.15)	.63
Intracranial or spinal embryonal tumors		
Overall	0.95 (0.88-1.02)	.19
Socioeconomic status	0.94 (0.88-1.02)	.13
Minority and language status	0.96 (0.89-1.03)	.26
Household composition	0.96 (0.89-1.03)	.28
Housing and transportation	0.98 (0.91-1.06)	.64
Ependymomas and choroid plexus tumors		
Overall	0.85 (0.71-1.01)	.06
Socioeconomic status	0.93 (0.78-1.11)	.41
Minority and language status	0.99 (0.84-1.18)	.95
Household composition	0.94 (0.79-1.12)	.47
Housing-transportation	0.85 (0.72-1.01)	.07
Hodgkin lymphomas		
Overall	1.09 (0.85-1.40)	.50
Socioeconomic status	1.11 (0.87-1.43)	.42
Minority and language status	1.07 (0.84-1.36)	.59
Household composition	0.98 (0.77-1.25)	.86
Housing and transportation	1.00 (0.79-1.29)	.97
Intracranial or spinal germ cell tumors		
Overall	1.07 (0.90-1.27)	.46
Socioeconomic status	1.06 (0.89-1.26)	.51
Minority and language status	1.09 (0.92-1.29)	.35
Household composition	1.02 (0.86-1.20)	.85
Housing and transportation	1.08 (0.91-1.29)	.37
Malignant melanomas		
Overall	1.00 (0.76-1.30)	.97
Socioeconomic status	1.05 (0.80-1.38)	.71
Minority and language status	1.07 (0.82-1.41)	.61
Household composition	1.06 (0.81-1.39)	.68
Housing and transportation	0.91 (0.69-1.18)	.47
Non-Hodgkin lymphoma		
Overall	1.21 (1.02-1.45)	.03
Socioeconomic status	1.27 (1.06-1.52)	.008
Minority and language status	1.04 (0.88-1.24)	.64
Household composition	1.11 (0.93-1.32)	.24
Housing-transportation	1.08 (0.91-1.28)	.37
Gliomas NOS		
Overall	1.08 (0.85-1.39)	.51
Socioeconomic status	1.15 (0.90-1.49)	.28
Minority and language status	1.30 (1.02-1.69)	.04
Household composition	0.83 (0.64-1.06)	.13
Housing and transportation	1.10 (0.87-1.41)	.42
Other or unspecified soft-tissue sarcomas		
Overall	0.99 (0.82-1.20)	.93
Socioeconomic status	1.03 (0.85-1.25)	.78
Minority and language status	1.08 (0.89-1.31)	.44
Household composition	0.91 (0.74-1.10)	.32
Housing and transportation	0.94 (0.78-1.14)	.54
Retinoblastomas		
Overall	1.31 (1.14-1.50)	<.001
Socioeconomic status	1.17 (1.02-1.34)	.02
Minority and language status	1.29 (1.13-1.48)	<.001
Household composition	1.01 (0.88-1.15)	.90
Housing and transportation	1.49 (1.29-1.73)	<.001
Rhabdomyosarcomas		
Overall	1.03 (0.92-1.16)	.60
Socioeconomic status	0.98 (0.88-1.10)	.76
Minority and language status	1.05 (0.94-1.18)	.39
Household composition	1.02 (0.91-1.14)	.76
Housing-transportation	1.20 (1.06-1.35)	.003
Thyroid carcinomas		
Overall	0.90 (0.78-1.03)	.13
Socioeconomic status	0.84 (0.73-0.96)	.01
Minority and language status	1.12 (0.98-1.29)	.10
Household composition	0.89 (0.78-1.02)	.10
Housing and transportation	0.97 (0.84-1.11)	.62

Abbreviations: ICCC, International Classification of Childhood Cancers; NOS, not otherwise specified; OR, odds ratio.

^a^ Univariate logistic regressions across Social Vulnerability Index quintiles based on first presentation occurrence of stage 4 or distant expansion for increasing overall Social Vulnerability Index score and subcomponent scores per disease class.

### ICCC Disease-Specific Analyses of Primary Surgery Receipt by Relative CDC-SVI Percentile

Across ICCC disease classes, increasing total CDC-SVI vulnerability was associated with decreased odds of receiving surgical intervention for those with malignant melanomas (OR, 0.79; 95% CI, 0.69-0.91; *P* = .001) and rhabdomyosarcomas (OR, 0.90; 95% CI, 0.83-0.98; *P* = .01). For both malignant melanomas and rhabdomyosarcomas, increasing SES, ML, and HT vulnerability, but not HH vulnerability, were associated with decreased odds of receiving surgery (Table 2).

**Table 2.** Association Between Primary Surgery Receipt and Increasing Social Vulnerability Index Score

ICCC class^a^	OR (95% CI)	*P* value
Astrocytomas		
Overall	0.99 (0.95-1.02)	.44
Socioeconomic status	0.97 (0.94-1.01)	.13
Minority and language status	1.02 (0.98-1.05)	.30
Household composition	0.97 (0.94-1.00)	.07
Housing and transportation	1.00 (0.97-1.04)	.86
Intracranial or spinal embryonal tumors		
Overall	0.98 (0.89-1.07)	.62
Socioeconomic status	1.01 (0.92-1.10)	.87
Minority and language status	0.98 (0.90-1.08)	.69
Household composition	0.97 (0.89-1.07)	.58
Housing and transportation	1.02 (0.93-1.12)	.70
Ependymomas and choroid plexus tumors		
Overall	1.07 (0.90-1.27)	.44
Socioeconomic status	1.07 (0.90-1.27)	.44
Minority and language status	1.06 (0.90-1.25)	.50
Household composition	1.06 (0.90-1.26)	.49
Housing and transportation	0.99 (0.84-1.18)	.95
Intracranial or spinal germ cell tumors		
Overall	1.03 (0.94-1.12)	.52
Socioeconomic status	1.10 (1.01-1.20)	.03
Minority and language status	0.92 (0.84-1.00)	.05
Household composition	1.07 (0.98-1.17)	.12
Housing and transportation	0.98 (0.90-1.07)	.68
Malignant melanomas		
Overall	0.79 (0.69-0.91)	.001
Socioeconomic status	0.81 (0.70-0.92)	.002
Minority and language status	0.84 (0.73-0.96)	.01
Household composition	1.00 (0.87-1.14)	.98
Housing and transportation	0.82 (0.71-0.93)	.003
Gliomas NOS		
Overall	0.97 (0.92-1.03)	.32
Socioeconomic status	0.96 (0.91-1.01)	.15
Minority and language status	0.99 (0.93-1.04)	.61
Household composition	0.97 (0.92-1.03)	.32
Housing and transportation	0.97 (0.92-1.03)	.30
Other or unspecified soft-tissue sarcomas		
Overall	0.92 (0.81-1.05)	.20
Socioeconomic status	0.88 (0.77-0.99)	.04
Minority and language status	0.93 (0.82-1.06)	.27
Household composition	0.94 (0.83-1.07)	.37
Housing and transportation	1.02 (0.90-1.15)	.81
Retinoblastomas		
Overall	1.08 (0.99-1.18)	.09
Socioeconomic status	1.05 (0.96-1.14)	.29
Minority and language status	1.03 (0.95-1.13)	.48
Household composition	1.06 (0.97-1.16)	.18
Housing and transportation	1.05 (0.96-1.14)	.29
Rhabdomyosarcomas		
Overall	0.90 (0.83-0.98)	.01
Socioeconomic status	0.95 (0.87-1.03)	.22
Minority and language status	0.90 (0.83-0.98)	.01
Household composition	1.01 (0.93-1.10)	.78
Housing and transportation	0.89 (0.82-0.97)	.007
Thyroid carcinomas		
Overall	0.93 (0.78-1.10)	.38
Socioeconomic status	0.95 (0.80-1.14)	.60
Minority and language status	1.00 (0.84-1.19)	.99
Household composition	0.98 (0.83-1.17)	.86
Housing and transportation	1.00 (0.84-1.19)	.99

Abbreviations: ICCC, International Classification of Childhood Cancers; NOS, not otherwise specified; OR, odds ratio.

^a^ Univariate logistic regressions across Social Vulnerability Index quintiles based on primary tumor resection for increasing total Social Vulnerability Index score and subcomponent scores per disease class.

## Discussion

To our knowledge, this is the first study to both use CDC-SVI as a comprehensive measure for evaluating SDoH across pediatric HNC and to evaluate the associations of specific SDoH themes with care and prognostic variables for patient with pediatric HNC. Overall, increasing social vulnerability measured by CDC-SVI and theme scores were associated with decreases in both months under surveillance and survival months for many ICCC disease classes within pediatric HNC as well as increased odds of advanced preliminary staging and decreased odds of primary surgery receipt for some ICCC disease classes.

As elucidated by the wide range of CDC-SVI scores within our study population, the need to assess the association of SDoH with pediatric health disparities is of universal importance. The development and evaluation of SDoH screening tools have seen increased attention within pediatrics.^18,19,20,21,22^ Sokol et al^19^ suggested that the various validated SDoH screening tools for children enable a myriad of methods to be given at the discretion of health care professionals based on the specific needs of their patients. Despite this diversity, evidence is lacking on which specific SDoH factors affect child health the most, owing to the difficulties of evaluating the accuracy of screenings when performed, the limited range of SDoH examined per method, and the few inquiries into the generalizability of one screening tool’s results to another.^19^ Furthermore, the many SDoH studies within pediatrics predominantly focus on noncancerous illnesses and outcomes.^21,23,24,25,26,27,28^ Thus, SDoH cancer-related studies in children and adolescents, let alone in pediatric HNC, are understudied.

Using the CDC-SVI themes, our current study presents a novel interpretation of how SDoH interact to affect the association of overall social vulnerability with pediatric HNC care and prognosis. Our method allowed specific diseases displaying significant overall SDoH disparities, as measured by total CDC-SVI score, to be assessed for not only which select SDoH types and themes were associated with this overall disparity but also how much each SDoH affects the interactional context of other SDoH. This provides a unique quantitative perspective within the realm of qualitative and complex relationships between varied SDoH.

For example, many significant differences in months’ surveillance and follow-up across ICCC disease classes saw the highest-magnitude decreases with increasing SES subscore (Figure 1). But when observing the significant differences in months’ survival, the highest-magnitude decreases were observed with vulnerabilities in non-SES–related themes (Figure 2). As seen with this comparison, the utility of our study methods reveals not only how different SDoH substantially contribute to the overall social vulnerability differences but also how there are quantitative differences in their associations when observing different aspects of HNC clinical characteristics (ie, surveillance vs survival). Moreover, these features dynamically change across disease types. For example, as seen in Figure 2, Hodgkin lymphoma survival had a larger association with HT vulnerability, whereas retinoblastomas were more associated with HH. These results further unveil nuanced aspects of our CDC-SVI–based SDoH analysis that go beyond general identification of SDoH factors.

Unlike using individual-level SDoH variables, the CDC-SVI uniquely assesses the sociodemographic contexts of the respective census tract or county by incorporating a dynamic, differential weighing of each theme subscore for calculating the overall CDC-SVI score. For example, one area’s total SVI could be derived from an even emphasis across all CDC-SVI themes (ie, 25% of each subscore accounts for the total CDC-SVI), while another would have differential emphasis on certain themes (eg, 32% on SES, 19% on ML, 21% on HH, 28% on HT) based on sociodemographic factors associated with the specific area as identified by the CDC-SVI. This feature provides added nuance that using individual-level SDoH alone cannot provide. For instance, 2 patients with similar levels of family income could have nonequivalent levels of health depending on their areas’ health resources, differentiating care based on income (eg, only one hospital that treats the entire local populace vs multiple clinics in the vicinity of varying quality), but the CDC-SVI would capture this sociodemographic context by adjusting the weighted emphasis on SES vulnerability in the CDC-SVI score. By accounting for these complex SDoH interactions, our CDC-SVI analysis could help to inform health care professionals about which SDoH should be discussed in leading further investigations, policies, and initiatives against specific pediatric HNC disparities.

### Strengths and Limitations

The principal strengths of our study are that it used a novel and comprehensive SDoH index to assess a variety of social vulnerabilities precisely measured by US Census tracts while providing quantifiable measures that combine the estimated effects of varied SDoH. The CDC-SVI themes highlight which SDoH contribute the most toward trends observed with the total CDC-SVI score. Additionally, the study encompassed nearly all pediatric HNC classes and included patient variables as well as level of care measurements and prognostic outcomes. Lastly, to our knowledge, it is the most comprehensive and contemporary analysis to date on the national trends in SDoH in pediatric HNC.

However, this study has limitations. The SEER database used in this study does not encompass the entirety of variables that would further characterize our findings due to the lack of data entries for specific variables of interest or the variable itself being inaccessible. Use of other SEER-associated databases that require paid access, such as SEER-Medicare linked databases, would provide additional information into operative details and treatment modalities beyond the stand-alone SEER and the means for future inquiry. In addition, most of the study population was White, which may have skewed our observed results. Despite some accountability taken within the CDC-SVI theme of ML status, future studies assessing for social vulnerability among different racial and ethnic groups should be performed. Also, despite its wide range of SDoH factors, the CDC-SVI does not measure all SDoH that would be of clinical interest, as more detailed measures accounting for sociodemographic contexts beyond its 15 social factors cannot be ascertained due to the design of CDC-SVI.

## Conclusions

In this cohort study of all patients with pediatric HNC in SEER and SDoH measures in the CDC-SVI, we found that increasing social vulnerability measured by the CDC-SVI and its theme scores were associated with decreases in both the surveillance and survival periods for many ICCC disease classes as well as increased odds of advanced preliminary staging and decreased odds of primary surgery receipt for some ICCC disease classes. Overall, these findings provide a quantitative and qualitative SDoH-based assessment of care and prognosis within pediatric HNC nationwide. Our results not only confirm anecdotal understandings of SDoH in pediatric HNC but also further explore the complex interactions across a multitude of SDoH through establishing an integrative measure applicable to patients from all US regions with differing sociodemographic and contextual influences. Furthermore, this study provided a method for identifying which and, more importantly, how much certain SDoH contribute to overall disparity trends in the contexts of varied, interacting SDoH factors. Our use of the CDC-SVI establishes the foundation for future inquiry into SDoH-related pediatric HNC studies and for advising health care professionals about which SDoH should be investigated to relay the most benefit against specific pediatric HNC disparities.

## Notes

Supplement 1. **eFigure 1.** Distribution of Total SVI and SVI Theme Ranked Scores Across the United States **eFigure 2.** Schematic Workflow of SVI and SEER Database Manipulation **eFigure 3.** Representative ICCC Disease Class Analysis: Linear Regression of Months’ Surveyed Trends With Increasing SVI Scores for Thyroid Carcinomas **eFigure 4.** Linear Regression of Months’ Surveyed Trends With Increasing SVI **eFigure 5.** Linear Regression of Months’ Survival Trends With Increasing SVI **eTable 1.** Patient Characteristics by SVI Score **eTable 2.** ICCC Disease Class Trends in Months Under Surveillance by Relative SVI Percentile **eTable 3.** ICCC Disease Class Trends in Survival Months by Relative SVI Percentile

Supplement 2.
Data Sharing Statement
