# Supplementary material for: Assessment of Social Vulnerability in Pediatric Head and Neck Cancer Care and Prognosis in the United States
Source: JAMA Netw Open. 2023 Feb 17;6(2):e230016. doi: 10.1001/jamanetworkopen.2023.0016 (PMC9938432; doi:10.1001/jamanetworkopen.2023.0016)
Supplement: Supplement 1. — eFigure 1. Distribution of Total SVI and SVI Theme Ranked Scores Across the United States eFigure 2. Schematic Workflow of SVI and SEER Database Manipulation eFigure 3. Representative ICCC Disease Class Analysis: Linear Regression of Months’ Surveyed Trends With Increasing SVI Scores for Thyroid Carcinomas eFigure 4. Linear Regression of Months’ Surveyed Trends With Increasing SVI eFigure 5. Linear Regression of Months’ Survival Trends With Increasing SVI eTable 1. Patient Characteristics by SVI Score eTable 2. ICCC Disease Class Trends in Months Under Surveillance by Relative SVI Percentile eTable 3. ICCC Disease Class Trends in Survival Months by Relative SVI Percentile [file jamanetwopen-e230016-s001.pdf]

# Supplemental Online Content

Fei-Zhang DJ, Chelius DC, Patel UA, Smith SS, Sheyn AM, Rastatter JC. Assessment of social vulnerability in pediatric head and neck cancer care and prognosis in the United States. *JAMA Netw Open.* 2023;6(2):e230016. doi:10.1001/jamanetworkopen.2023.0016

**eFigure 1.** Distribution of Total SVI and SVI Theme Ranked Scores Across the United States

**eFigure 2.** Schematic Workflow of SVI and SEER Database Manipulation

**eFigure 3.** Representative ICCC Disease Class Analysis: Linear Regression of Months' Surveyed Trends With Increasing SVI Scores for Thyroid Carcinomas

**eFigure 4.** Linear Regression of Months' Surveyed Trends With Increasing SVI

**eFigure 5.** Linear Regression of Months' Survival Trends With Increasing SVI

**eTable 1.** Patient Characteristics by SVI Score

**eTable 2.** ICCC Disease Class Trends in Months Under Surveillance by Relative SVI Percentile

**eTable 3.** ICCC Disease Class Trends in Survival Months by Relative SVI Percentile

This supplemental material has been provided by the authors to give readers additional information about their work.

© 2023 Fei-Zhang DJ et al. *JAMA Network Open.*

**eFigure 1.** Distribution of Total SVI and SVI Theme Ranked Scores Across the United States

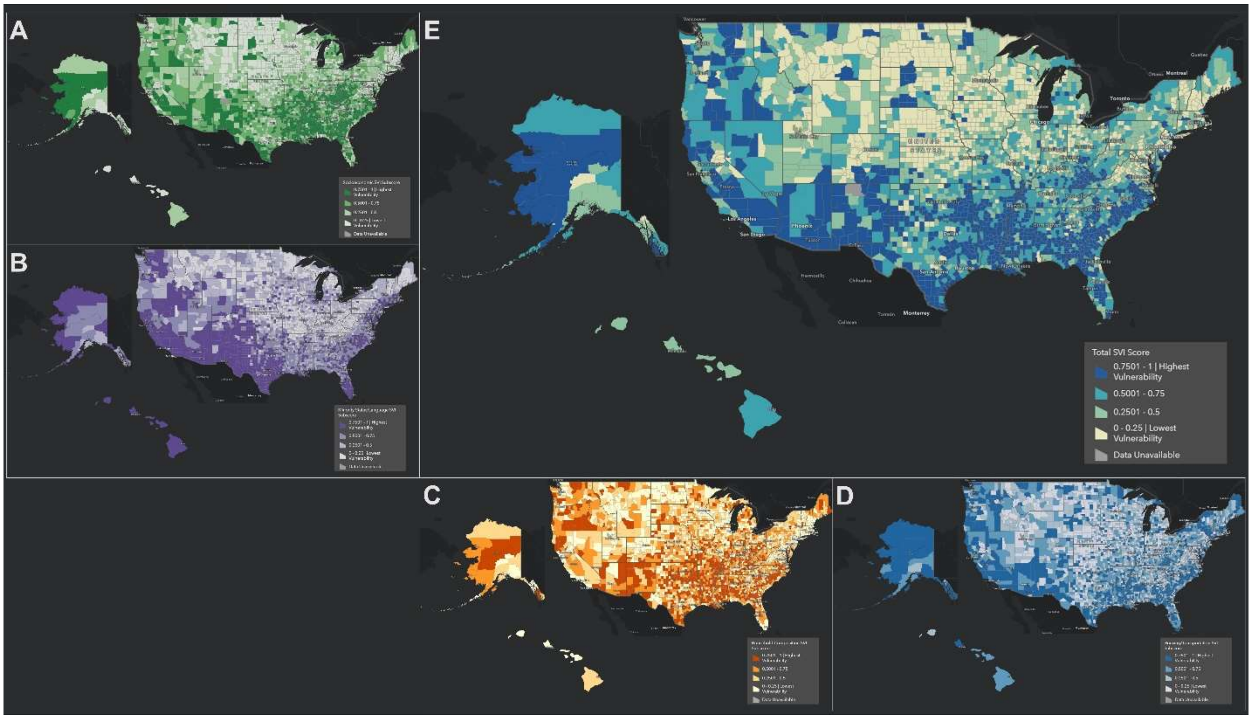


© 2023 Fei-Zhang DJ et al. *JAMA Network Open.*

**eFigure 2.** Schematic Workflow of SVI and SEER Database Manipulation

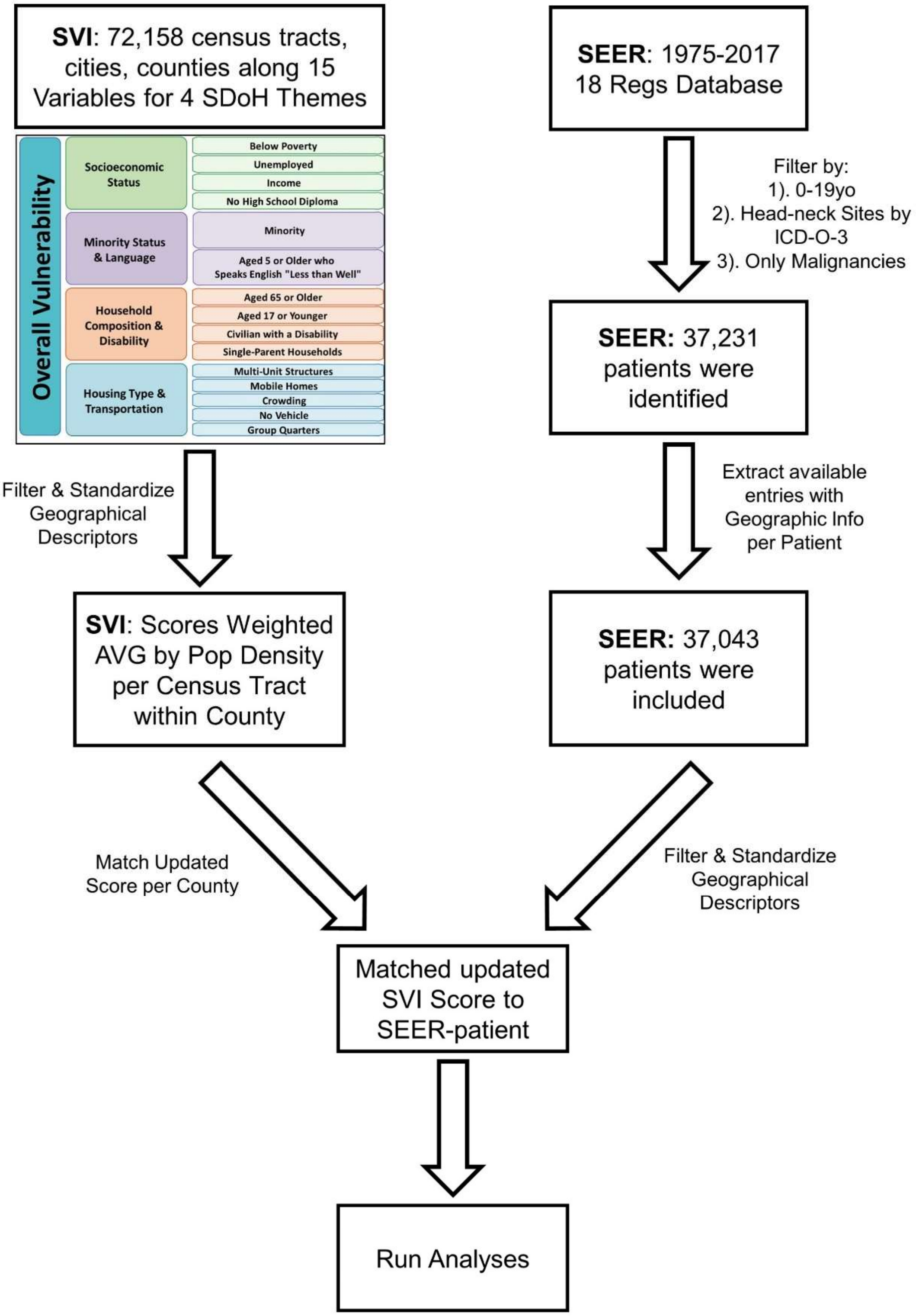


© 2023 Fei-Zhang DJ et al. *JAMA Network Open.*

**eFigure 3.** Representative ICCC Disease Class Analysis: Linear Regression of Months' Surveyed Trends With Increasing SVI Scores for Thyroid Carcinomas

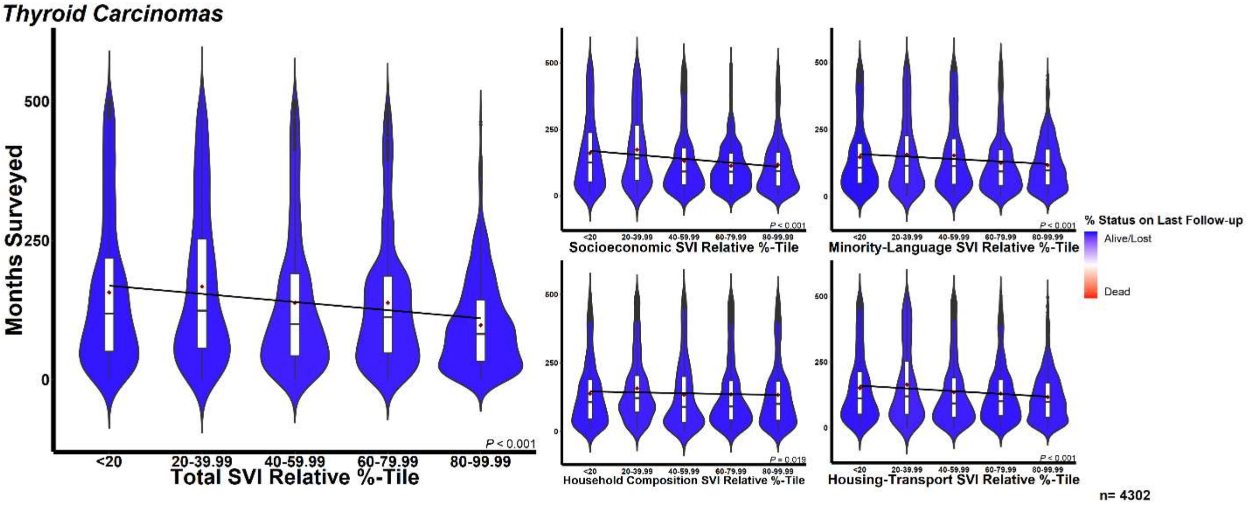


© 2023 Fei-Zhang DJ et al. *JAMA Network Open.*

**eFigure 4.** Linear Regression of Months' Surveyed Trends With Increasing SVI

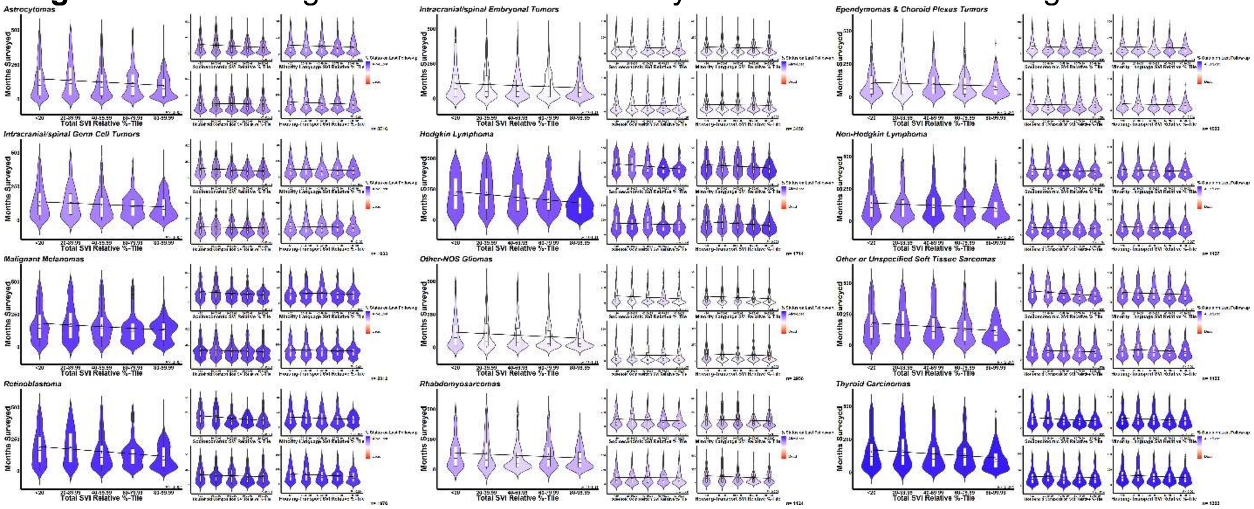


© 2023 Fei-Zhang DJ et al. *JAMA Network Open.*

**eFigure 5.** Linear Regression of Months' Survival Trends With Increasing SVI

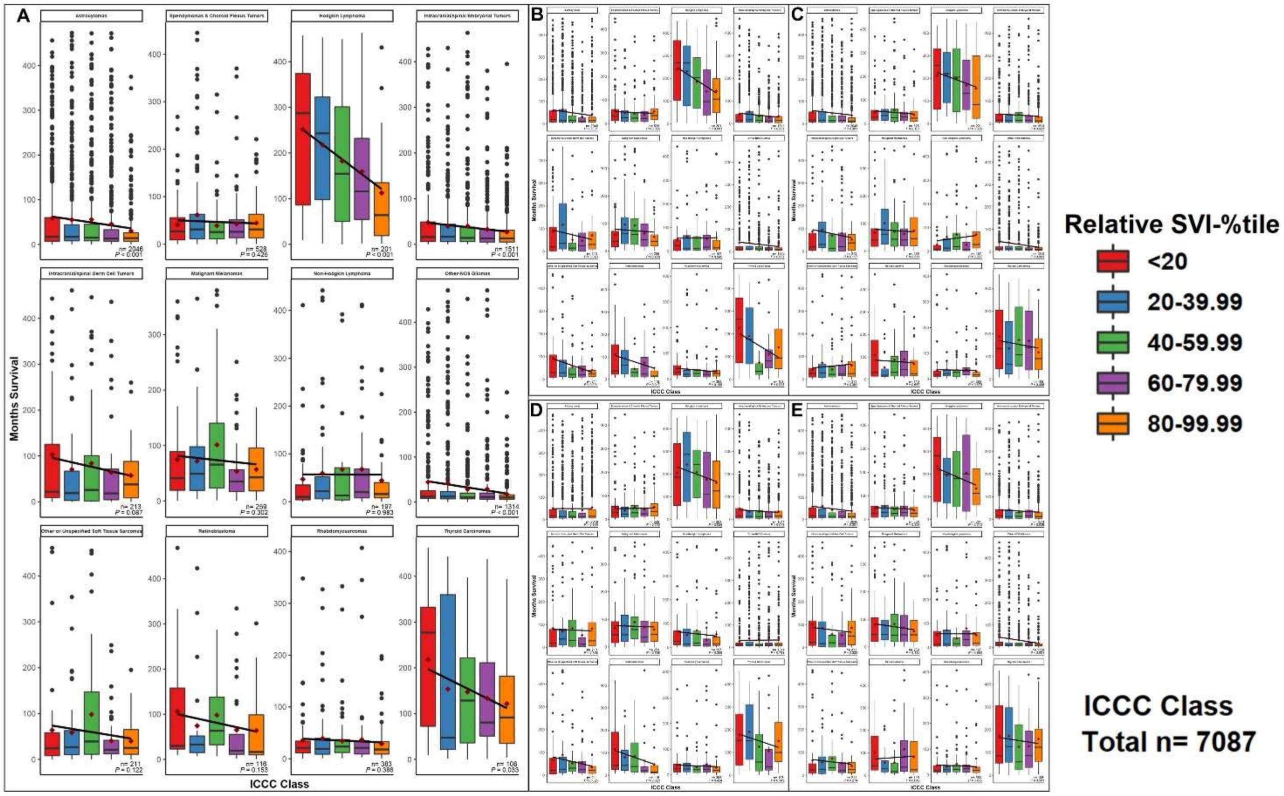


© 2023 Fei-Zhang DJ et al. *JAMA Network Open.*

## eTable 1. Patient Characteristics by SVI Score

| | | Total SVI Score | | | | |
|---|---|---|---|---|---|---|
| Characteristic | N | 0.000-0.199, N = 280 (0.8%) | 0.200-0.399, N = 8809 (24%) | 0.400-0.599, N = 17991 (49%) | 0.600-0.799, N = 9326 (25%) | 0.800-0.999, N = 637 (1.7%) |
| **Age** | 37,043 | | | | | |
| 0-9 years | | 110 (39%) | 3,738 (42%) | 7,938 (44%) | 4,228 (45%) | 300 (47%) |
| 10-19 years | | 170 (61%) | 5,071 (58%) | 10,053 (56%) | 5,098 (55%) | 337 (53%) |
| **Sex** | 37,043 | | | | | |
| Male | | 135 (48%) | 4,337 (49%) | 9,103 (51%) | 4,713 (51%) | 315 (49%) |
| Female | | 145 (52%) | 4,472 (51%) | 8,888 (49%) | 4,613 (49%) | 322 (51%) |
| **Race** | 37,043 | | | | | |
| White | | 236 (84%) | 7,192 (82%) | 10,795 (60%) | 3,949 (42%) | 258 (41%) |
| Hispanic | | 28 (10%) | 697 (7.9%) | 2,854 (16%) | 3,628 (39%) | 269 (42%) |
| Black | | 5 (1.8%) | 451 (5.1%) | 2,333 (13%) | 974 (10%) | 57 (8.9%) |
| Asian or Pacific Islander | | 5 (1.8%) | 313 (3.6%) | 1,644 (9.1%) | 597 (6.4%) | 10 (1.6%) |
| Unknown | | 1 (0.4%) | 126 (1.4%) | 267 (1.5%) | 127 (1.4%) | 1 (0.2%) |
| Native American | | 5 (1.8%) | 30 (0.3%) | 98 (0.5%) | 51 (0.5%) | 42 (6.6%) |
| **Region** | 37,043 | | | | | |
| Midwest | | 27 (9.6%) | 1,932 (22%) | 3,097 (17%) | 94 (1.0%) | 0 (0%) |
| Northeast | | 97 (35%) | 2,616 (30%) | 2,264 (13%) | 420 (4.5%) | 0 (0%) |
| South | | 93 (33%) | 1,011 (11%) | 3,413 (19%) | 1,787 (19%) | 168 (26%) |
| West | | 63 (22%) | 3,250 (37%) | 9,217 (51%) | 7,025 (75%) | 469 (74%) |
| **Primary Site in Head-Neck** | 36,968 | | | | | |
| Blood Plasma | | 1 (0.4%) | 2 (<0.1%) | 2 (<0.1%) | 6 (<0.1%) | 0 (0%) |
| Bones & Joints | | 12 (4.3%) | 299 (3.4%) | 620 (3.5%) | 380 (4.1%) | 21 (3.3%) |
| CNS & PNS | | 119 (42%) | 4,335 (49%) | 8,506 (47%) | 4,390 (47%) | 294 (46%) |
| Eye & Orbit | | 14 (5.0%) | 484 (5.5%) | 1,140 (6.4%) | 659 (7.1%) | 46 (7.2%) |
| Hypopharynx | | 0 (0%) | 1 (<0.1%) | 2 (<0.1%) | 1 (<0.1%) | 0 (0%) |

© 2023 Fei-Zhang DJ et al. *JAMA Network Open.*

| | | Total SVI Score | | | | |
|---|---|---|---|---|---|---|
| Characteristic | N | 0.000-0.199, N = 280 (0.8%) | 0.200-0.399, N = 8809 (24%) | 0.400-0.599, N = 17991 (49%) | 0.600-0.799, N = 9326 (25%) | 0.800-0.999, N = 637 (1.7%) |
| Larynx | | 0 (0%) | 3 (<0.1%) | 14 (<0.1%) | 8 (<0.1%) | 0 (0%) |
| Lip | | 0 (0%) | 3 (<0.1%) | 14 (<0.1%) | 4 (<0.1%) | 1 (0.2%) |
| Lymphatic | | 28 (10%) | 805 (9.1%) | 1,771 (9.9%) | 860 (9.2%) | 78 (12%) |
| Misc. | | 0 (0%) | 41 (0.5%) | 95 (0.5%) | 60 (0.6%) | 4 (0.6%) |
| Mouth | | 0 (0%) | 4 (<0.1%) | 7 (<0.1%) | 4 (<0.1%) | 2 (0.3%) |
| Mouth, Oral Cavity, & Pharynx | | 0 (0%) | 47 (0.5%) | 118 (0.7%) | 58 (0.6%) | 1 (0.2%) |
| Nasopharynx | | 2 (0.7%) | 61 (0.7%) | 236 (1.3%) | 127 (1.4%) | 16 (2.5%) |
| Non-Thyroid Endocrine | | 7 (2.5%) | 177 (2.0%) | 468 (2.6%) | 276 (3.0%) | 12 (1.9%) |
| Nose & Middle Ear | | 2 (0.7%) | 60 (0.7%) | 140 (0.8%) | 85 (0.9%) | 9 (1.4%) |
| Oropharynx | | 0 (0%) | 0 (0%) | 9 (<0.1%) | 1 (<0.1%) | 0 (0%) |
| Salivary Gland | | 3 (1.1%) | 111 (1.3%) | 258 (1.4%) | 130 (1.4%) | 7 (1.1%) |
| Skin | | 45 (16%) | 937 (11%) | 1,591 (8.9%) | 707 (7.6%) | 43 (6.8%) |
| Soft Tissue | | 13 (4.6%) | 373 (4.2%) | 816 (4.5%) | 420 (4.5%) | 35 (5.5%) |
| Thyroid | | 34 (12%) | 1,032 (12%) | 2,091 (12%) | 1,103 (12%) | 64 (10%) |
| Tongue | | 0 (0%) | 20 (0.2%) | 43 (0.2%) | 17 (0.2%) | 2 (0.3%) |
| Tonsil | | 0 (0%) | 2 (<0.1%) | 5 (<0.1%) | 4 (<0.1%) | 0 (0%) |
| Trachea | | 0 (0%) | 3 (<0.1%) | 5 (<0.1%) | 2 (<0.1%) | 0 (0%) |
| **ICCC Class** | 37,043 | | | | | |
| Acute Myeloid Leukemia | | 0 (0%) | 0 (0%) | 1 (<0.1%) | 0 (0%) | 0 (0%) |
| Astrocytomas | | 62 (22%) | 2,221 (25%) | 4,198 (23%) | 2,083 (22%) | 152 (24%) |
| Burkitt Lymphoma | | 4 (1.4%) | 68 (0.8%) | 177 (1.0%) | 78 (0.8%) | 3 (0.5%) |
| Chondrosarcomas | | 2 (0.7%) | 17 (0.2%) | 50 (0.3%) | 33 (0.4%) | 2 (0.3%) |

© 2023 Fei-Zhang DJ et al. *JAMA Network Open.*

| | | Total SVI Score | | | | |
|---|---|---|---|---|---|---|
| Characteristic | N | 0.000-0.199, N = 280 (0.8%) | 0.200-0.399, N = 8809 (24%) | 0.400-0.599, N = 17991 (49%) | 0.600-0.799, N = 9326 (25%) | 0.800-0.999, N = 637 (1.7%) |
| Ependymomas & Choroid Plexus Tumors | | 5 (1.8%) | 354 (4.0%) | 703 (3.9%) | 413 (4.4%) | 27 (4.2%) |
| Ewing Tumors & Related Bone Sarcomas | | 7 (2.5%) | 120 (1.4%) | 217 (1.2%) | 123 (1.3%) | 7 (1.1%) |
| Extracranial Germ Cell Tumors | | 0 (0%) | 12 (0.1%) | 47 (0.3%) | 20 (0.2%) | 5 (0.8%) |
| Fibrosarcomas, PNS Sheath Tumors, Other Fibrous Neoplasms | | 3 (1.1%) | 81 (0.9%) | 131 (0.7%) | 71 (0.8%) | 9 (1.4%) |
| Hodgkin Lymphoma | | 12 (4.3%) | 419 (4.8%) | 841 (4.7%) | 408 (4.4%) | 34 (5.3%) |
| Intracranial/spinal Embryonal Tumors | | 24 (8.6%) | 756 (8.6%) | 1,656 (9.2%) | 953 (10%) | 61 (9.6%) |
| Intracranial/spinal Germ Cell Tumors | | 7 (2.5%) | 204 (2.3%) | 504 (2.8%) | 304 (3.3%) | 14 (2.2%) |
| Kaposi Sarcoma | | 0 (0%) | 4 (<0.1%) | 5 (<0.1%) | 3 (<0.1%) | 1 (0.2%) |
| Malignant Melanomas | | 41 (15%) | 830 (9.4%) | 1,341 (7.5%) | 566 (6.1%) | 34 (5.3%) |
| Nasopharyngeal Carcinomas | | 1 (0.4%) | 33 (0.4%) | 183 (1.0%) | 90 (1.0%) | 15 (2.4%) |
| Neuroblastomas & Ganglioneuroblastomas | | 1 (0.4%) | 95 (1.1%) | 169 (0.9%) | 58 (0.6%) | 5 (0.8%) |
| Non-Hodgkin Lymphoma | | 13 (4.6%) | 302 (3.4%) | 709 (3.9%) | 363 (3.9%) | 40 (6.3%) |
| Osteosarcomas | | 3 (1.1%) | 111 (1.3%) | 263 (1.5%) | 147 (1.6%) | 8 (1.3%) |
| Other-NOS Gliomas | | 22 (7.9%) | 747 (8.5%) | 1,473 (8.2%) | 675 (7.2%) | 39 (6.1%) |

© 2023 Fei-Zhang DJ et al. *JAMA Network Open.*

| | | Total SVI Score | | | | |
|---|---|---|---|---|---|---|
| Characteristic | N | 0.000-0.199, N = 280 (0.8%) | 0.200-0.399, N = 8809 (24%) | 0.400-0.599, N = 17991 (49%) | 0.600-0.799, N = 9326 (25%) | 0.800-0.999, N = 637 (1.7%) |
| Other or Unspecified Carcinomas | | 4 (1.4%) | 185 (2.1%) | 458 (2.5%) | 216 (2.3%) | 11 (1.7%) |
| Other or Unspecified Intracranial/spinal CNS Tumors | | 4 (1.4%) | 111 (1.3%) | 256 (1.4%) | 142 (1.5%) | 10 (1.6%) |
| Other or Unspecified Leukemias | | 0 (0%) | 8 (<0.1%) | 25 (0.1%) | 21 (0.2%) | 2 (0.3%) |
| Other or Unspecified Lymphomas | | 0 (0%) | 50 (0.6%) | 120 (0.7%) | 64 (0.7%) | 4 (0.6%) |
| Other or Unspecified Malignant Bone Tumors | | 0 (0%) | 48 (0.5%) | 81 (0.5%) | 62 (0.7%) | 4 (0.6%) |
| Other or Unspecified Malignant Neoplasms | | 2 (0.7%) | 13 (0.1%) | 23 (0.1%) | 12 (0.1%) | 1 (0.2%) |
| Other or Unspecified PNS Tumors | | 1 (0.4%) | 22 (0.2%) | 25 (0.1%) | 21 (0.2%) | 1 (0.2%) |
| Other or Unspecified Soft Tissue Sarcomas | | 10 (3.6%) | 315 (3.6%) | 676 (3.8%) | 379 (4.1%) | 23 (3.6%) |
| Retinoblastoma | | 11 (3.9%) | 388 (4.4%) | 963 (5.4%) | 573 (6.1%) | 41 (6.4%) |
| Rhabdomyosarcomas | | 9 (3.2%) | 246 (2.8%) | 552 (3.1%) | 299 (3.2%) | 18 (2.8%) |
| Skin Carcinomas | | 0 (0%) | 5 (<0.1%) | 23 (0.1%) | 18 (0.2%) | 2 (0.3%) |
| Thyroid Carcinomas | | 32 (11%) | 1,025 (12%) | 2,082 (12%) | 1,099 (12%) | 64 (10%) |
| Unknown | | 0 (0%) | 19 (0.2%) | 39 (0.2%) | 32 (0.3%) | 0 (0%) |
| **Vital Status on Last Follow-up** | 37,043 | | | | | |
| Alive | | 241 (86%) | 6,860 (78%) | 13,691 (76%) | 7,372 (79%) | 489 (77%) |

© 2023 Fei-Zhang DJ et al. *JAMA Network Open.*

| | | Total SVI Score | | | | |
|---|---|---|---|---|---|---|
| Characteristic | N | 0.000-0.199, N = 280 (0.8%) | 0.200-0.399, N = 8809 (24%) | 0.400-0.599, N = 17991 (49%) | 0.600-0.799, N = 9326 (25%) | 0.800-0.999, N = 637 (1.7%) |
| Dead | | 39 (14%) | 1,949 (22%) | 4,300 (24%) | 1,954 (21%) | 148 (23%) |

| | | Socioeconomic SVI Subscore | | | | |
|---|---|---|---|---|---|---|
| Characteristic | N | 0.000-0.199, N = 663 (1.8%) | 0.200-0.399, N = 13131 (35%) | 0.400-0.599, N = 15936 (43%) | 0.600-0.799, N = 6660 (18%) | 0.800-0.999, N = 653 (1.8%) |
| **Age** | 37,043 | | | | | |
| 0-9 years | | 271 (41%) | 5,648 (43%) | 7,078 (44%) | 3,012 (45%) | 305 (47%) |
| 10-19 years | | 392 (59%) | 7,483 (57%) | 8,858 (56%) | 3,648 (55%) | 348 (53%) |
| **Sex** | 37,043 | | | | | |
| Male | | 323 (49%) | 6,623 (50%) | 8,011 (50%) | 3,310 (50%) | 336 (51%) |
| Female | | 340 (51%) | 6,508 (50%) | 7,925 (50%) | 3,350 (50%) | 317 (49%) |
| **Race** | 37,043 | | | | | |
| White | | 506 (76%) | 9,213 (70%) | 8,781 (55%) | 3,527 (53%) | 403 (62%) |
| Hispanic | | 82 (12%) | 1,499 (11%) | 4,165 (26%) | 1,601 (24%) | 129 (20%) |
| Black | | 17 (2.6%) | 797 (6.1%) | 1,707 (11%) | 1,224 (18%) | 75 (11%) |
| Asian or Pacific Islander | | 35 (5.3%) | 1,374 (10%) | 971 (6.1%) | 185 (2.8%) | 4 (0.6%) |
| Unknown | | 17 (2.6%) | 201 (1.5%) | 236 (1.5%) | 61 (0.9%) | 7 (1.1%) |
| Native American | | 6 (0.9%) | 47 (0.4%) | 76 (0.5%) | 62 (0.9%) | 35 (5.4%) |
| **Region** | 37,043 | | | | | |
| Midwest | | 56 (8.4%) | 2,327 (18%) | 1,303 (8.2%) | 1,464 (22%) | 0 (0%) |
| Northeast | | 339 (51%) | 2,966 (23%) | 2,038 (13%) | 54 (0.8%) | 0 (0%) |
| South | | 29 (4.4%) | 691 (5.3%) | 3,254 (20%) | 2,056 (31%) | 442 (68%) |
| West | | 239 (36%) | 7,147 (54%) | 9,341 (59%) | 3,086 (46%) | 211 (32%) |
| **Primary Site in Head-Neck** | 36,968 | | | | | |
| Blood Plasma | | 0 (0%) | 4 (<0.1%) | 4 (<0.1%) | 3 (<0.1%) | 0 (0%) |
| Bones & Joints | | 24 (3.6%) | 452 (3.4%) | 589 (3.7%) | 246 (3.7%) | 21 (3.2%) |

© 2023 Fei-Zhang DJ et al. *JAMA Network Open.*

| | | Socioeconomic SVI Subscore | | | | |
|---|---|---|---|---|---|---|
| Characteristic | N | 0.000-0.199, N = 663 (1.8%) | 0.200-0.399, N = 13131 (35%) | 0.400-0.599, N = 15936 (43%) | 0.600-0.799, N = 6660 (18%) | 0.800-0.999, N = 653 (1.8%) |
| CNS & PNS | | 313 (47%) | 6,343 (48%) | 7,440 (47%) | 3,217 (48%) | 331 (51%) |
| Eye & Orbit | | 39 (5.9%) | 775 (5.9%) | 1,038 (6.5%) | 446 (6.7%) | 45 (6.9%) |
| Hypopharynx | | 0 (0%) | 2 (<0.1%) | 1 (<0.1%) | 1 (<0.1%) | 0 (0%) |
| Larynx | | 0 (0%) | 4 (<0.1%) | 16 (0.1%) | 5 (<0.1%) | 0 (0%) |
| Lip | | 0 (0%) | 8 (<0.1%) | 10 (<0.1%) | 3 (<0.1%) | 1 (0.2%) |
| Lymphatic | | 67 (10%) | 1,236 (9.4%) | 1,510 (9.5%) | 663 (10.0%) | 66 (10%) |
| Misc. | | 0 (0%) | 79 (0.6%) | 90 (0.6%) | 26 (0.4%) | 5 (0.8%) |
| Mouth | | 0 (0%) | 7 (<0.1%) | 5 (<0.1%) | 4 (<0.1%) | 1 (0.2%) |
| Mouth, Oral Cavity, & Pharynx | | 4 (0.6%) | 68 (0.5%) | 115 (0.7%) | 35 (0.5%) | 2 (0.3%) |
| Nasopharynx | | 4 (0.6%) | 128 (1.0%) | 200 (1.3%) | 100 (1.5%) | 10 (1.5%) |
| Non-Thyroid Endocrine | | 17 (2.6%) | 325 (2.5%) | 400 (2.5%) | 191 (2.9%) | 7 (1.1%) |
| Nose & Middle Ear | | 6 (0.9%) | 98 (0.7%) | 123 (0.8%) | 62 (0.9%) | 7 (1.1%) |
| Oropharynx | | 0 (0%) | 5 (<0.1%) | 5 (<0.1%) | 0 (0%) | 0 (0%) |
| Salivary Gland | | 6 (0.9%) | 171 (1.3%) | 234 (1.5%) | 93 (1.4%) | 5 (0.8%) |
| Skin | | 61 (9.2%) | 1,351 (10%) | 1,378 (8.7%) | 478 (7.2%) | 55 (8.4%) |
| Soft Tissue | | 26 (3.9%) | 510 (3.9%) | 787 (5.0%) | 306 (4.6%) | 28 (4.3%) |
| Thyroid | | 95 (14%) | 1,506 (11%) | 1,905 (12%) | 752 (11%) | 66 (10%) |
| Tongue | | 1 (0.2%) | 32 (0.2%) | 34 (0.2%) | 13 (0.2%) | 2 (0.3%) |
| Tonsil | | 0 (0%) | 4 (<0.1%) | 6 (<0.1%) | 1 (<0.1%) | 0 (0%) |
| Trachea | | 0 (0%) | 3 (<0.1%) | 6 (<0.1%) | 1 (<0.1%) | 0 (0%) |
| **ICCC Class** | 37,043 | | | | | |
| Acute Myeloid Leukemia | | 0 (0%) | 0 (0%) | 1 (<0.1%) | 0 (0%) | 0 (0%) |
| Astrocytomas | | 158 (24%) | 3,172 (24%) | 3,628 (23%) | 1,589 (24%) | 169 (26%) |

© 2023 Fei-Zhang DJ et al. *JAMA Network Open.*

| | | Socioeconomic SVI Subscore | | | | |
|---|---|---|---|---|---|---|
| Characteristic | N | 0.000-0.199, N = 663 (1.8%) | 0.200-0.399, N = 13131 (35%) | 0.400-0.599, N = 15936 (43%) | 0.600-0.799, N = 6660 (18%) | 0.800-0.999, N = 653 (1.8%) |
| Burkitt Lymphoma | | 5 (0.8%) | 106 (0.8%) | 155 (1.0%) | 57 (0.9%) | 7 (1.1%) |
| Chondrosarcomas | | 3 (0.5%) | 40 (0.3%) | 39 (0.2%) | 20 (0.3%) | 2 (0.3%) |
| Ependymomas & Choroid Plexus Tumors | | 34 (5.1%) | 506 (3.9%) | 652 (4.1%) | 282 (4.2%) | 28 (4.3%) |
| Ewing Tumors & Related Bone Sarcomas | | 6 (0.9%) | 180 (1.4%) | 204 (1.3%) | 75 (1.1%) | 9 (1.4%) |
| Extracranial Germ Cell Tumors | | 0 (0%) | 29 (0.2%) | 30 (0.2%) | 22 (0.3%) | 3 (0.5%) |
| Fibrosarcomas, PNS Sheath Tumors, Other Fibrous Neoplasms | | 5 (0.8%) | 102 (0.8%) | 134 (0.8%) | 46 (0.7%) | 8 (1.2%) |
| Hodgkin Lymphoma | | 32 (4.8%) | 622 (4.7%) | 696 (4.4%) | 331 (5.0%) | 33 (5.1%) |
| Intracranial/spinal Embryonal Tumors | | 51 (7.7%) | 1,203 (9.2%) | 1,500 (9.4%) | 632 (9.5%) | 64 (9.8%) |
| Intracranial/spinal Germ Cell Tumors | | 11 (1.7%) | 373 (2.8%) | 452 (2.8%) | 186 (2.8%) | 11 (1.7%) |
| Kaposi Sarcoma | | 1 (0.2%) | 6 (<0.1%) | 4 (<0.1%) | 2 (<0.1%) | 0 (0%) |
| Malignant Melanomas | | 58 (8.7%) | 1,151 (8.8%) | 1,178 (7.4%) | 380 (5.7%) | 45 (6.9%) |
| Nasopharyngeal Carcinomas | | 1 (0.2%) | 86 (0.7%) | 149 (0.9%) | 77 (1.2%) | 9 (1.4%) |
| Neuroblastomas & Ganglioneuroblastomas | | 3 (0.5%) | 132 (1.0%) | 127 (0.8%) | 60 (0.9%) | 6 (0.9%) |
| Non-Hodgkin Lymphoma | | 30 (4.5%) | 487 (3.7%) | 630 (4.0%) | 254 (3.8%) | 26 (4.0%) |

© 2023 Fei-Zhang DJ et al. *JAMA Network Open.*

| | | Socioeconomic SVI Subscore | | | | |
|---|---|---|---|---|---|---|
| Characteristic | N | 0.000-0.199, N = 663 (1.8%) | 0.200-0.399, N = 13131 (35%) | 0.400-0.599, N = 15936 (43%) | 0.600-0.799, N = 6660 (18%) | 0.800-0.999, N = 653 (1.8%) |
| Osteosarcomas | | 8 (1.2%) | 167 (1.3%) | 246 (1.5%) | 101 (1.5%) | 10 (1.5%) |
| Other-NOS Gliomas | | 55 (8.3%) | 1,090 (8.3%) | 1,245 (7.8%) | 516 (7.7%) | 50 (7.7%) |
| Other or Unspecified Carcinomas | | 14 (2.1%) | 287 (2.2%) | 416 (2.6%) | 147 (2.2%) | 10 (1.5%) |
| Other or Unspecified Intracranial/spinal CNS Tumors | | 12 (1.8%) | 161 (1.2%) | 207 (1.3%) | 134 (2.0%) | 9 (1.4%) |
| Other or Unspecified Leukemias | | 0 (0%) | 11 (<0.1%) | 32 (0.2%) | 11 (0.2%) | 2 (0.3%) |
| Other or Unspecified Lymphomas | | 0 (0%) | 90 (0.7%) | 105 (0.7%) | 40 (0.6%) | 3 (0.5%) |
| Other or Unspecified Malignant Bone Tumors | | 5 (0.8%) | 58 (0.4%) | 92 (0.6%) | 39 (0.6%) | 1 (0.2%) |
| Other or Unspecified Malignant Neoplasms | | 0 (0%) | 20 (0.2%) | 19 (0.1%) | 11 (0.2%) | 1 (0.2%) |
| Other or Unspecified PNS Tumors | | 2 (0.3%) | 29 (0.2%) | 21 (0.1%) | 18 (0.3%) | 0 (0%) |
| Other or Unspecified Soft Tissue Sarcomas | | 19 (2.9%) | 482 (3.7%) | 619 (3.9%) | 263 (3.9%) | 20 (3.1%) |
| Retinoblastoma | | 31 (4.7%) | 639 (4.9%) | 895 (5.6%) | 371 (5.6%) | 40 (6.1%) |
| Rhabdomyosarcomas | | 22 (3.3%) | 362 (2.8%) | 501 (3.1%) | 221 (3.3%) | 18 (2.8%) |
| Skin Carcinomas | | 1 (0.2%) | 13 (<0.1%) | 21 (0.1%) | 10 (0.2%) | 3 (0.5%) |
| Thyroid Carcinomas | | 95 (14%) | 1,495 (11%) | 1,897 (12%) | 749 (11%) | 66 (10%) |
| Unknown | | 1 (0.2%) | 32 (0.2%) | 41 (0.3%) | 16 (0.2%) | 0 (0%) |

© 2023 Fei-Zhang DJ et al. *JAMA Network Open.*

| | | Socioeconomic SVI Subscore | | | | |
|---|---|---|---|---|---|---|
| Characteristic | N | 0.000-0.199, N = 663 (1.8%) | 0.200-0.399, N = 13131 (35%) | 0.400-0.599, N = 15936 (43%) | 0.600-0.799, N = 6660 (18%) | 0.800-0.999, N = 653 (1.8%) |
| **Vital Status on Last Follow-up** | 37,043 | | | | | |
| Alive | | 543 (82%) | 9,913 (75%) | 12,561 (79%) | 5,130 (77%) | 506 (77%) |
| Dead | | 120 (18%) | 3,218 (25%) | 3,375 (21%) | 1,530 (23%) | 147 (23%) |

| | | Minority-Language SVI Subscore | | | | |
|---|---|---|---|---|---|---|
| Characteristic | N | 0.000-0.199, N = 1435 (3.9%) | 0.200-0.399, N = 4832 (13%) | 0.400-0.599, N = 11866 (32%) | 0.600-0.799, N = 11506 (31%) | 0.800-0.999, N = 7404 (20%) |
| **Age** | 37,043 | | | | | |
| 0-9 years | | 634 (44%) | 2,124 (44%) | 5,162 (44%) | 4,987 (43%) | 3,407 (46%) |
| 10-19 years | | 801 (56%) | 2,708 (56%) | 6,704 (56%) | 6,519 (57%) | 3,997 (54%) |
| **Sex** | 37,043 | | | | | |
| Male | | 725 (51%) | 2,454 (51%) | 5,801 (49%) | 5,847 (51%) | 3,776 (51%) |
| Female | | 710 (49%) | 2,378 (49%) | 6,065 (51%) | 5,659 (49%) | 3,628 (49%) |
| **Race** | 37,043 | | | | | |
| White | | 1,380 (96%) | 4,178 (86%) | 8,539 (72%) | 5,908 (51%) | 2,425 (33%) |
| Hispanic | | 14 (1.0%) | 232 (4.8%) | 946 (8.0%) | 2,979 (26%) | 3,305 (45%) |
| Black | | 24 (1.7%) | 288 (6.0%) | 1,871 (16%) | 991 (8.6%) | 646 (8.7%) |
| Asian or Pacific Islander | | 4 (0.3%) | 53 (1.1%) | 302 (2.5%) | 1,344 (12%) | 866 (12%) |
| Unknown | | 12 (0.8%) | 61 (1.3%) | 141 (1.2%) | 185 (1.6%) | 123 (1.7%) |
| Native American | | 1 (<0.1%) | 20 (0.4%) | 67 (0.6%) | 99 (0.9%) | 39 (0.5%) |
| **Region** | 37,043 | | | | | |
| Midwest | | 687 (48%) | 955 (20%) | 3,495 (29%) | 13 (0.1%) | 0 (0%) |
| Northeast | | 0 (0%) | 1,068 (22%) | 2,685 (23%) | 1,494 (13%) | 150 (2.0%) |
| South | | 734 (51%) | 1,331 (28%) | 2,744 (23%) | 1,536 (13%) | 127 (1.7%) |
| West | | 14 (1.0%) | 1,478 (31%) | 2,942 (25%) | 8,463 (74%) | 7,127 (96%) |

© 2023 Fei-Zhang DJ et al. *JAMA Network Open.*

| | | Minority-Language SVI Subscore | | | | |
|---|---|---|---|---|---|---|
| Characteristic | N | 0.000-0.199, N = 1435 (3.9%) | 0.200-0.399, N = 4832 (13%) | 0.400-0.599, N = 11866 (32%) | 0.600-0.799, N = 11506 (31%) | 0.800-0.999, N = 7404 (20%) |
| **Primary Site in Head-Neck** | 36,968 | | | | | |
| Blood Plasma | | 0 (0%) | 1 (<0.1%) | 2 (<0.1%) | 4 (<0.1%) | 4 (<0.1%) |
| Bones & Joints | | 58 (4.0%) | 160 (3.3%) | 410 (3.5%) | 414 (3.6%) | 290 (3.9%) |
| CNS & PNS | | 700 (49%) | 2,439 (51%) | 5,719 (48%) | 5,339 (46%) | 3,447 (47%) |
| Eye & Orbit | | 87 (6.1%) | 287 (5.9%) | 712 (6.0%) | 709 (6.2%) | 548 (7.4%) |
| Hypopharynx | | 0 (0%) | 0 (0%) | 3 (<0.1%) | 1 (<0.1%) | 0 (0%) |
| Larynx | | 2 (0.1%) | 3 (<0.1%) | 6 (<0.1%) | 7 (<0.1%) | 7 (<0.1%) |
| Lip | | 0 (0%) | 2 (<0.1%) | 8 (<0.1%) | 5 (<0.1%) | 7 (<0.1%) |
| Lymphatic | | 130 (9.1%) | 413 (8.6%) | 1,231 (10%) | 1,103 (9.6%) | 665 (9.0%) |
| Misc. | | 3 (0.2%) | 14 (0.3%) | 58 (0.5%) | 69 (0.6%) | 56 (0.8%) |
| Mouth | | 0 (0%) | 0 (0%) | 7 (<0.1%) | 6 (<0.1%) | 4 (<0.1%) |
| Mouth, Oral Cavity, & Pharynx | | 7 (0.5%) | 28 (0.6%) | 75 (0.6%) | 73 (0.6%) | 41 (0.6%) |
| Nasopharynx | | 14 (1.0%) | 43 (0.9%) | 131 (1.1%) | 155 (1.3%) | 99 (1.3%) |
| Non-Thyroid Endocrine | | 26 (1.8%) | 111 (2.3%) | 262 (2.2%) | 296 (2.6%) | 245 (3.3%) |
| Nose & Middle Ear | | 11 (0.8%) | 37 (0.8%) | 102 (0.9%) | 75 (0.7%) | 71 (1.0%) |
| Oropharynx | | 0 (0%) | 0 (0%) | 4 (<0.1%) | 4 (<0.1%) | 2 (<0.1%) |
| Salivary Gland | | 15 (1.0%) | 64 (1.3%) | 160 (1.4%) | 156 (1.4%) | 114 (1.5%) |
| Skin | | 139 (9.7%) | 481 (10.0%) | 1,044 (8.8%) | 1,116 (9.7%) | 543 (7.4%) |
| Soft Tissue | | 61 (4.3%) | 186 (3.9%) | 528 (4.5%) | 541 (4.7%) | 341 (4.6%) |
| Thyroid | | 178 (12%) | 538 (11%) | 1,361 (11%) | 1,377 (12%) | 870 (12%) |
| Tongue | | 4 (0.3%) | 16 (0.3%) | 20 (0.2%) | 23 (0.2%) | 19 (0.3%) |
| Tonsil | | 0 (0%) | 2 (<0.1%) | 2 (<0.1%) | 3 (<0.1%) | 4 (<0.1%) |
| Trachea | | 0 (0%) | 0 (0%) | 3 (<0.1%) | 7 (<0.1%) | 0 (0%) |
| **ICCC Class** | 37,043 | | | | | |

© 2023 Fei-Zhang DJ et al. *JAMA Network Open.*

| | | Minority-Language SVI Subscore | | | | |
|---|---|---|---|---|---|---|
| Characteristic | N | 0.000-0.199, N = 1435 (3.9%) | 0.200-0.399, N = 4832 (13%) | 0.400-0.599, N = 11866 (32%) | 0.600-0.799, N = 11506 (31%) | 0.800-0.999, N = 7404 (20%) |
| Acute Myeloid Leukemia | | 0 (0%) | 0 (0%) | 1 (<0.1%) | 0 (0%) | 0 (0%) |
| Astrocytomas | | 376 (26%) | 1,261 (26%) | 2,851 (24%) | 2,614 (23%) | 1,614 (22%) |
| Burkitt Lymphoma | | 19 (1.3%) | 42 (0.9%) | 102 (0.9%) | 104 (0.9%) | 63 (0.9%) |
| Chondrosarcomas | | 9 (0.6%) | 11 (0.2%) | 35 (0.3%) | 29 (0.3%) | 20 (0.3%) |
| Ependymomas & Choroid Plexus Tumors | | 52 (3.6%) | 185 (3.8%) | 489 (4.1%) | 433 (3.8%) | 343 (4.6%) |
| Ewing Tumors & Related Bone Sarcomas | | 21 (1.5%) | 61 (1.3%) | 150 (1.3%) | 147 (1.3%) | 95 (1.3%) |
| Extracranial Germ Cell Tumors | | 3 (0.2%) | 9 (0.2%) | 22 (0.2%) | 34 (0.3%) | 16 (0.2%) |
| Fibrosarcomas, PNS Sheath Tumors, Other Fibrous Neoplasms | | 13 (0.9%) | 36 (0.7%) | 101 (0.9%) | 80 (0.7%) | 65 (0.9%) |
| Hodgkin Lymphoma | | 63 (4.4%) | 222 (4.6%) | 650 (5.5%) | 488 (4.2%) | 291 (3.9%) |
| Intracranial/spinal Embryonal Tumors | | 118 (8.2%) | 411 (8.5%) | 1,047 (8.8%) | 1,097 (9.5%) | 777 (10%) |
| Intracranial/spinal Germ Cell Tumors | | 27 (1.9%) | 111 (2.3%) | 258 (2.2%) | 359 (3.1%) | 278 (3.8%) |
| Kaposi Sarcoma | | 0 (0%) | 0 (0%) | 3 (<0.1%) | 6 (<0.1%) | 4 (<0.1%) |
| Malignant Melanomas | | 125 (8.7%) | 441 (9.1%) | 873 (7.4%) | 948 (8.2%) | 425 (5.7%) |
| Nasopharyngeal Carcinomas | | 7 (0.5%) | 26 (0.5%) | 101 (0.9%) | 110 (1.0%) | 78 (1.1%) |

© 2023 Fei-Zhang DJ et al. *JAMA Network Open.*

| | | Minority-Language SVI Subscore | | | | |
|---|---|---|---|---|---|---|
| Characteristic | N | 0.000-0.199, N = 1435 (3.9%) | 0.200-0.399, N = 4832 (13%) | 0.400-0.599, N = 11866 (32%) | 0.600-0.799, N = 11506 (31%) | 0.800-0.999, N = 7404 (20%) |
| Neuroblastomas & Ganglioneuroblastomas | | 11 (0.8%) | 47 (1.0%) | 120 (1.0%) | 98 (0.9%) | 52 (0.7%) |
| Non-Hodgkin Lymphoma | | 46 (3.2%) | 142 (2.9%) | 444 (3.7%) | 491 (4.3%) | 304 (4.1%) |
| Osteosarcomas | | 20 (1.4%) | 61 (1.3%) | 168 (1.4%) | 167 (1.5%) | 116 (1.6%) |
| Other-NOS Gliomas | | 122 (8.5%) | 431 (8.9%) | 1,014 (8.5%) | 886 (7.7%) | 503 (6.8%) |
| Other or Unspecified Carcinomas | | 24 (1.7%) | 105 (2.2%) | 285 (2.4%) | 270 (2.3%) | 190 (2.6%) |
| Other or Unspecified Intracranial/spinal CNS Tumors | | 19 (1.3%) | 78 (1.6%) | 190 (1.6%) | 133 (1.2%) | 103 (1.4%) |
| Other or Unspecified Leukemias | | 0 (0%) | 2 (<0.1%) | 14 (0.1%) | 25 (0.2%) | 15 (0.2%) |
| Other or Unspecified Lymphomas | | 4 (0.3%) | 21 (0.4%) | 80 (0.7%) | 72 (0.6%) | 61 (0.8%) |
| Other or Unspecified Malignant Bone Tumors | | 7 (0.5%) | 26 (0.5%) | 54 (0.5%) | 58 (0.5%) | 50 (0.7%) |
| Other or Unspecified Malignant Neoplasms | | 0 (0%) | 8 (0.2%) | 19 (0.2%) | 11 (<0.1%) | 13 (0.2%) |
| Other or Unspecified PNS Tumors | | 4 (0.3%) | 16 (0.3%) | 19 (0.2%) | 18 (0.2%) | 13 (0.2%) |
| Other or Unspecified Soft Tissue Sarcomas | | 48 (3.3%) | 159 (3.3%) | 428 (3.6%) | 465 (4.0%) | 303 (4.1%) |
| Retinoblastoma | | 77 (5.4%) | 234 (4.8%) | 582 (4.9%) | 604 (5.2%) | 479 (6.5%) |

© 2023 Fei-Zhang DJ et al. *JAMA Network Open.*

| | | Minority-Language SVI Subscore | | | | |
|---|---|---|---|---|---|---|
| Characteristic | N | 0.000-0.199, N = 1435 (3.9%) | 0.200-0.399, N = 4832 (13%) | 0.400-0.599, N = 11866 (32%) | 0.600-0.799, N = 11506 (31%) | 0.800-0.999, N = 7404 (20%) |
| Rhabdomyosarcomas | | 39 (2.7%) | 141 (2.9%) | 372 (3.1%) | 343 (3.0%) | 229 (3.1%) |
| Skin Carcinomas | | 3 (0.2%) | 2 (<0.1%) | 13 (0.1%) | 16 (0.1%) | 14 (0.2%) |
| Thyroid Carcinomas | | 177 (12%) | 532 (11%) | 1,353 (11%) | 1,373 (12%) | 867 (12%) |
| Unknown | | 1 (<0.1%) | 11 (0.2%) | 28 (0.2%) | 27 (0.2%) | 23 (0.3%) |
| **Vital Status on Last Follow-up** | 37,043 | | | | | |
| Alive | | 1,129 (79%) | 3,816 (79%) | 8,996 (76%) | 8,994 (78%) | 5,718 (77%) |
| Dead | | 306 (21%) | 1,016 (21%) | 2,870 (24%) | 2,512 (22%) | 1,686 (23%) |

| | | Household Composition SVI Subscore | | | | |
|---|---|---|---|---|---|---|
| Characteristic | N | 0.000-0.199, N = 469 (1.3%) | 0.200-0.399, N = 17629 (48%) | 0.400-0.599, N = 11830 (32%) | 0.600-0.799, N = 6358 (17%) | 0.800-0.999, N = 757 (2.0%) |
| **Age** | 37,043 | | | | | |
| 0-9 years | | 187 (40%) | 7,747 (44%) | 5,160 (44%) | 2,865 (45%) | 355 (47%) |
| 10-19 years | | 282 (60%) | 9,882 (56%) | 6,670 (56%) | 3,493 (55%) | 402 (53%) |
| **Sex** | 37,043 | | | | | |
| Male | | 242 (52%) | 8,899 (50%) | 5,897 (50%) | 3,182 (50%) | 383 (51%) |
| Female | | 227 (48%) | 8,730 (50%) | 5,933 (50%) | 3,176 (50%) | 374 (49%) |
| **Race** | 37,043 | | | | | |
| White | | 238 (51%) | 9,877 (56%) | 7,689 (65%) | 4,096 (64%) | 530 (70%) |
| Hispanic | | 60 (13%) | 4,278 (24%) | 2,111 (18%) | 933 (15%) | 94 (12%) |
| Black | | 56 (12%) | 1,339 (7.6%) | 1,309 (11%) | 1,029 (16%) | 87 (11%) |
| Asian or Pacific Islander | | 107 (23%) | 1,792 (10%) | 495 (4.2%) | 172 (2.7%) | 3 (0.4%) |
| Unknown | | 8 (1.7%) | 290 (1.6%) | 164 (1.4%) | 56 (0.9%) | 4 (0.5%) |
| Native American | | 0 (0%) | 53 (0.3%) | 62 (0.5%) | 72 (1.1%) | 39 (5.2%) |
| **Region** | 37,043 | | | | | |

© 2023 Fei-Zhang DJ et al. *JAMA Network Open.*

| | | Household Composition SVI Subscore | | | | |
|---|---|---|---|---|---|---|
| Characteristic | N | 0.000-0.199, N = 469 (1.3%) | 0.200-0.399, N = 17629 (48%) | 0.400-0.599, N = 11830 (32%) | 0.600-0.799, N = 6358 (17%) | 0.800-0.999, N = 757 (2.0%) |
| Midwest | | 76 (16%) | 1,010 (5.7%) | 1,700 (14%) | 2,332 (37%) | 32 (4.2%) |
| Northeast | | 36 (7.7%) | 2,942 (17%) | 2,365 (20%) | 54 (0.8%) | 0 (0%) |
| South | | 0 (0%) | 1,692 (9.6%) | 2,255 (19%) | 2,017 (32%) | 508 (67%) |
| West | | 357 (76%) | 11,985 (68%) | 5,510 (47%) | 1,955 (31%) | 217 (29%) |
| **Primary Site in Head-Neck** | 36,968 | | | | | |
| Blood Plasma | | 0 (0%) | 6 (<0.1%) | 5 (<0.1%) | 0 (0%) | 0 (0%) |
| Bones & Joints | | 17 (3.6%) | 639 (3.6%) | 403 (3.4%) | 248 (3.9%) | 25 (3.3%) |
| CNS & PNS | | 193 (41%) | 8,346 (47%) | 5,595 (47%) | 3,143 (49%) | 367 (49%) |
| Eye & Orbit | | 35 (7.5%) | 1,131 (6.4%) | 714 (6.0%) | 412 (6.5%) | 51 (6.8%) |
| Hypopharynx | | 0 (0%) | 2 (<0.1%) | 2 (<0.1%) | 0 (0%) | 0 (0%) |
| Larynx | | 0 (0%) | 10 (<0.1%) | 7 (<0.1%) | 8 (0.1%) | 0 (0%) |
| Lip | | 0 (0%) | 10 (<0.1%) | 8 (<0.1%) | 3 (<0.1%) | 1 (0.1%) |
| Lymphatic | | 42 (9.0%) | 1,605 (9.1%) | 1,216 (10%) | 599 (9.4%) | 80 (11%) |
| Misc. | | 5 (1.1%) | 113 (0.6%) | 56 (0.5%) | 22 (0.3%) | 4 (0.5%) |
| Mouth | | 0 (0%) | 7 (<0.1%) | 6 (<0.1%) | 3 (<0.1%) | 1 (0.1%) |
| Mouth, Oral Cavity, & Pharynx | | 2 (0.4%) | 98 (0.6%) | 83 (0.7%) | 35 (0.6%) | 6 (0.8%) |
| Nasopharynx | | 10 (2.1%) | 191 (1.1%) | 134 (1.1%) | 98 (1.5%) | 9 (1.2%) |
| Non-Thyroid Endocrine | | 21 (4.5%) | 475 (2.7%) | 274 (2.3%) | 150 (2.4%) | 20 (2.6%) |
| Nose & Middle Ear | | 2 (0.4%) | 137 (0.8%) | 88 (0.7%) | 61 (1.0%) | 8 (1.1%) |
| Oropharynx | | 0 (0%) | 5 (<0.1%) | 5 (<0.1%) | 0 (0%) | 0 (0%) |
| Salivary Gland | | 6 (1.3%) | 237 (1.3%) | 170 (1.4%) | 87 (1.4%) | 9 (1.2%) |
| Skin | | 44 (9.4%) | 1,651 (9.4%) | 1,088 (9.2%) | 481 (7.6%) | 59 (7.8%) |
| Soft Tissue | | 28 (6.0%) | 789 (4.5%) | 531 (4.5%) | 277 (4.4%) | 32 (4.2%) |
| Thyroid | | 62 (13%) | 2,089 (12%) | 1,391 (12%) | 701 (11%) | 81 (11%) |

© 2023 Fei-Zhang DJ et al. *JAMA Network Open.*

| | | Household Composition SVI Subscore | | | | |
|---|---|---|---|---|---|---|
| Characteristic | N | 0.000-0.199, N = 469 (1.3%) | 0.200-0.399, N = 17629 (48%) | 0.400-0.599, N = 11830 (32%) | 0.600-0.799, N = 6358 (17%) | 0.800-0.999, N = 757 (2.0%) |
| Tongue | | 2 (0.4%) | 38 (0.2%) | 22 (0.2%) | 18 (0.3%) | 2 (0.3%) |
| Tonsil | | 0 (0%) | 6 (<0.1%) | 4 (<0.1%) | 1 (<0.1%) | 0 (0%) |
| Trachea | | 0 (0%) | 4 (<0.1%) | 3 (<0.1%) | 3 (<0.1%) | 0 (0%) |
| **ICCC Class** | 37,043 | | | | | |
| Acute Myeloid Leukemia | | 0 (0%) | 0 (0%) | 1 (<0.1%) | 0 (0%) | 0 (0%) |
| Astrocytomas | | 93 (20%) | 4,071 (23%) | 2,766 (23%) | 1,603 (25%) | 183 (24%) |
| Burkitt Lymphoma | | 5 (1.1%) | 144 (0.8%) | 113 (1.0%) | 63 (1.0%) | 5 (0.7%) |
| Chondrosarcomas | | 1 (0.2%) | 43 (0.2%) | 34 (0.3%) | 23 (0.4%) | 3 (0.4%) |
| Ependymomas & Choroid Plexus Tumors | | 20 (4.3%) | 722 (4.1%) | 464 (3.9%) | 271 (4.3%) | 25 (3.3%) |
| Ewing Tumors & Related Bone Sarcomas | | 7 (1.5%) | 231 (1.3%) | 148 (1.3%) | 81 (1.3%) | 7 (0.9%) |
| Extracranial Germ Cell Tumors | | 1 (0.2%) | 36 (0.2%) | 27 (0.2%) | 15 (0.2%) | 5 (0.7%) |
| Fibrosarcomas, PNS Sheath Tumors, Other Fibrous Neoplasms | | 8 (1.7%) | 150 (0.9%) | 82 (0.7%) | 43 (0.7%) | 12 (1.6%) |
| Hodgkin Lymphoma | | 15 (3.2%) | 754 (4.3%) | 610 (5.2%) | 293 (4.6%) | 42 (5.5%) |
| Intracranial/spinal Embryonal Tumors | | 28 (6.0%) | 1,675 (9.5%) | 1,106 (9.3%) | 566 (8.9%) | 75 (9.9%) |
| Intracranial/spinal Germ Cell Tumors | | 23 (4.9%) | 542 (3.1%) | 307 (2.6%) | 143 (2.2%) | 18 (2.4%) |
| Kaposi Sarcoma | | 2 (0.4%) | 9 (<0.1%) | 0 (0%) | 2 (<0.1%) | 0 (0%) |

© 2023 Fei-Zhang DJ et al. *JAMA Network Open.*

| | | Household Composition SVI Subscore | | | | |
|---|---|---|---|---|---|---|
| Characteristic | N | 0.000-0.199, N = 469 (1.3%) | 0.200-0.399, N = 17629 (48%) | 0.400-0.599, N = 11830 (32%) | 0.600-0.799, N = 6358 (17%) | 0.800-0.999, N = 757 (2.0%) |
| Malignant Melanomas | | 35 (7.5%) | 1,399 (7.9%) | 931 (7.9%) | 396 (6.2%) | 51 (6.7%) |
| Nasopharyngeal Carcinomas | | 9 (1.9%) | 134 (0.8%) | 97 (0.8%) | 75 (1.2%) | 7 (0.9%) |
| Neuroblastomas & Ganglioneuroblastomas | | 4 (0.9%) | 143 (0.8%) | 118 (1.0%) | 58 (0.9%) | 5 (0.7%) |
| Non-Hodgkin Lymphoma | | 21 (4.5%) | 683 (3.9%) | 470 (4.0%) | 221 (3.5%) | 32 (4.2%) |
| Osteosarcomas | | 6 (1.3%) | 266 (1.5%) | 144 (1.2%) | 106 (1.7%) | 10 (1.3%) |
| Other-NOS Gliomas | | 42 (9.0%) | 1,399 (7.9%) | 938 (7.9%) | 516 (8.1%) | 61 (8.1%) |
| Other or Unspecified Carcinomas | | 9 (1.9%) | 410 (2.3%) | 291 (2.5%) | 147 (2.3%) | 17 (2.2%) |
| Other or Unspecified Intracranial/spinal CNS Tumors | | 4 (0.9%) | 230 (1.3%) | 150 (1.3%) | 122 (1.9%) | 17 (2.2%) |
| Other or Unspecified Leukemias | | 0 (0%) | 29 (0.2%) | 20 (0.2%) | 5 (<0.1%) | 2 (0.3%) |
| Other or Unspecified Lymphomas | | 5 (1.1%) | 126 (0.7%) | 65 (0.5%) | 39 (0.6%) | 3 (0.4%) |
| Other or Unspecified Malignant Bone Tumors | | 3 (0.6%) | 88 (0.5%) | 64 (0.5%) | 35 (0.6%) | 5 (0.7%) |
| Other or Unspecified Malignant Neoplasms | | 0 (0%) | 28 (0.2%) | 14 (0.1%) | 8 (0.1%) | 1 (0.1%) |
| Other or Unspecified PNS Tumors | | 1 (0.2%) | 35 (0.2%) | 16 (0.1%) | 17 (0.3%) | 1 (0.1%) |

© 2023 Fei-Zhang DJ et al. *JAMA Network Open.*

| | | Household Composition SVI Subscore | | | | |
|---|---|---|---|---|---|---|
| Characteristic | N | 0.000-0.199, N = 469 (1.3%) | 0.200-0.399, N = 17629 (48%) | 0.400-0.599, N = 11830 (32%) | 0.600-0.799, N = 6358 (17%) | 0.800-0.999, N = 757 (2.0%) |
| Other or Unspecified Soft Tissue Sarcomas | | 20 (4.3%) | 666 (3.8%) | 453 (3.8%) | 244 (3.8%) | 20 (2.6%) |
| Retinoblastoma | | 30 (6.4%) | 959 (5.4%) | 598 (5.1%) | 348 (5.5%) | 41 (5.4%) |
| Rhabdomyosarcomas | | 15 (3.2%) | 509 (2.9%) | 378 (3.2%) | 196 (3.1%) | 26 (3.4%) |
| Skin Carcinomas | | 0 (0%) | 25 (0.1%) | 13 (0.1%) | 8 (0.1%) | 2 (0.3%) |
| Thyroid Carcinomas | | 62 (13%) | 2,077 (12%) | 1,383 (12%) | 700 (11%) | 80 (11%) |
| Unknown | | 0 (0%) | 46 (0.3%) | 29 (0.2%) | 14 (0.2%) | 1 (0.1%) |
| **Vital Status on Last Follow-up** | 37,043 | | | | | |
| Alive | | 345 (74%) | 13,660 (77%) | 9,260 (78%) | 4,789 (75%) | 599 (79%) |
| Dead | | 124 (26%) | 3,969 (23%) | 2,570 (22%) | 1,569 (25%) | 158 (21%) |

| | | Housing-Transport SVI Subscore | | | | |
|---|---|---|---|---|---|---|
| Characteristic | N | 0.000-0.199, N = 82 (0.2%) | 0.200-0.399, N = 7440 (20%) | 0.400-0.599, N = 19399 (52%) | 0.600-0.799, N = 9948 (27%) | 0.800-0.999, N = 174 (0.5%) |
| **Age** | 37,043 | | | | | |
| 0-9 years | | 36 (44%) | 3,133 (42%) | 8,579 (44%) | 4,487 (45%) | 79 (45%) |
| 10-19 years | | 46 (56%) | 4,307 (58%) | 10,820 (56%) | 5,461 (55%) | 95 (55%) |
| **Sex** | 37,043 | | | | | |
| Male | | 39 (48%) | 3,670 (49%) | 9,773 (50%) | 5,021 (50%) | 100 (57%) |
| Female | | 43 (52%) | 3,770 (51%) | 9,626 (50%) | 4,927 (50%) | 74 (43%) |
| **Race** | 37,043 | | | | | |
| White | | 72 (88%) | 6,004 (81%) | 11,469 (59%) | 4,795 (48%) | 90 (52%) |
| Hispanic | | 5 (6.1%) | 601 (8.1%) | 3,645 (19%) | 3,202 (32%) | 23 (13%) |
| Black | | 4 (4.9%) | 539 (7.2%) | 2,421 (12%) | 833 (8.4%) | 23 (13%) |
| Asian or Pacific Islander | | 1 (1.2%) | 185 (2.5%) | 1,472 (7.6%) | 911 (9.2%) | 0 (0%) |

© 2023 Fei-Zhang DJ et al. *JAMA Network Open.*

| | | Housing-Transport SVI Subscore | | | | |
|---|---|---|---|---|---|---|
| Characteristic | N | 0.000-0.199, N = 82 (0.2%) | 0.200-0.399, N = 7440 (20%) | 0.400-0.599, N = 19399 (52%) | 0.600-0.799, N = 9948 (27%) | 0.800-0.999, N = 174 (0.5%) |
| Unknown | | 0 (0%) | 86 (1.2%) | 279 (1.4%) | 157 (1.6%) | 0 (0%) |
| Native American | | 0 (0%) | 25 (0.3%) | 113 (0.6%) | 50 (0.5%) | 38 (22%) |
| **Region** | 37,043 | | | | | |
| Midwest | | 0 (0%) | 1,695 (23%) | 3,118 (16%) | 324 (3.3%) | 13 (7.5%) |
| Northeast | | 0 (0%) | 2,139 (29%) | 3,108 (16%) | 150 (1.5%) | 0 (0%) |
| South | | 70 (85%) | 1,836 (25%) | 3,423 (18%) | 1,072 (11%) | 71 (41%) |
| West | | 12 (15%) | 1,770 (24%) | 9,750 (50%) | 8,402 (84%) | 90 (52%) |
| **Primary Site in Head-Neck** | 36,968 | | | | | |
| Blood Plasma | | 0 (0%) | 3 (<0.1%) | 5 (<0.1%) | 3 (<0.1%) | 0 (0%) |
| Bones & Joints | | 6 (7.3%) | 247 (3.3%) | 698 (3.6%) | 374 (3.8%) | 7 (4.0%) |
| CNS & PNS | | 31 (38%) | 3,567 (48%) | 9,300 (48%) | 4,660 (47%) | 86 (49%) |
| Eye & Orbit | | 4 (4.9%) | 389 (5.2%) | 1,251 (6.5%) | 688 (6.9%) | 11 (6.3%) |
| Hypopharynx | | 0 (0%) | 1 (<0.1%) | 3 (<0.1%) | 0 (0%) | 0 (0%) |
| Larynx | | 0 (0%) | 2 (<0.1%) | 14 (<0.1%) | 9 (<0.1%) | 0 (0%) |
| Lip | | 0 (0%) | 2 (<0.1%) | 14 (<0.1%) | 5 (<0.1%) | 1 (0.6%) |
| Lymphatic | | 10 (12%) | 766 (10%) | 1,851 (9.6%) | 899 (9.1%) | 16 (9.2%) |
| Misc. | | 0 (0%) | 28 (0.4%) | 95 (0.5%) | 73 (0.7%) | 4 (2.3%) |
| Mouth | | 0 (0%) | 5 (<0.1%) | 9 (<0.1%) | 2 (<0.1%) | 1 (0.6%) |
| Mouth, Oral Cavity, & Pharynx | | 0 (0%) | 40 (0.5%) | 123 (0.6%) | 60 (0.6%) | 1 (0.6%) |
| Nasopharynx | | 0 (0%) | 60 (0.8%) | 239 (1.2%) | 140 (1.4%) | 3 (1.7%) |
| Non-Thyroid Endocrine | | 4 (4.9%) | 153 (2.1%) | 491 (2.5%) | 289 (2.9%) | 3 (1.7%) |
| Nose & Middle Ear | | 0 (0%) | 59 (0.8%) | 150 (0.8%) | 84 (0.8%) | 3 (1.7%) |
| Oropharynx | | 0 (0%) | 0 (0%) | 8 (<0.1%) | 2 (<0.1%) | 0 (0%) |
| Salivary Gland | | 1 (1.2%) | 102 (1.4%) | 272 (1.4%) | 132 (1.3%) | 2 (1.1%) |

© 2023 Fei-Zhang DJ et al. *JAMA Network Open.*

| | | Housing-Transport SVI Subscore | | | | |
|---|---|---|---|---|---|---|
| Characteristic | N | 0.000-0.199, N = 82 (0.2%) | 0.200-0.399, N = 7440 (20%) | 0.400-0.599, N = 19399 (52%) | 0.600-0.799, N = 9948 (27%) | 0.800-0.999, N = 174 (0.5%) |
| Skin | | 16 (20%) | 764 (10%) | 1,673 (8.6%) | 859 (8.7%) | 11 (6.3%) |
| Soft Tissue | | 3 (3.7%) | 331 (4.5%) | 871 (4.5%) | 445 (4.5%) | 7 (4.0%) |
| Thyroid | | 7 (8.5%) | 892 (12%) | 2,242 (12%) | 1,166 (12%) | 17 (9.8%) |
| Tongue | | 0 (0%) | 16 (0.2%) | 42 (0.2%) | 23 (0.2%) | 1 (0.6%) |
| Tonsil | | 0 (0%) | 3 (<0.1%) | 3 (<0.1%) | 5 (<0.1%) | 0 (0%) |
| Trachea | | 0 (0%) | 3 (<0.1%) | 5 (<0.1%) | 2 (<0.1%) | 0 (0%) |
| **ICCC Class** | 37,043 | | | | | |
| Acute Myeloid Leukemia | | 0 (0%) | 0 (0%) | 1 (<0.1%) | 0 (0%) | 0 (0%) |
| Astrocytomas | | 19 (23%) | 1,844 (25%) | 4,576 (24%) | 2,238 (22%) | 39 (22%) |
| Burkitt Lymphoma | | 2 (2.4%) | 59 (0.8%) | 186 (1.0%) | 82 (0.8%) | 1 (0.6%) |
| Chondrosarcomas | | 0 (0%) | 15 (0.2%) | 62 (0.3%) | 27 (0.3%) | 0 (0%) |
| Ependymomas & Choroid Plexus Tumors | | 1 (1.2%) | 281 (3.8%) | 794 (4.1%) | 419 (4.2%) | 7 (4.0%) |
| Ewing Tumors & Related Bone Sarcomas | | 4 (4.9%) | 103 (1.4%) | 230 (1.2%) | 134 (1.3%) | 3 (1.7%) |
| Extracranial Germ Cell Tumors | | 0 (0%) | 9 (0.1%) | 50 (0.3%) | 24 (0.2%) | 1 (0.6%) |
| Fibrosarcomas, PNS Sheath Tumors, Other Fibrous Neoplasms | | 1 (1.2%) | 68 (0.9%) | 142 (0.7%) | 82 (0.8%) | 2 (1.1%) |
| Hodgkin Lymphoma | | 5 (6.1%) | 399 (5.4%) | 883 (4.6%) | 424 (4.3%) | 3 (1.7%) |
| Intracranial/spinal Embryonal Tumors | | 6 (7.3%) | 641 (8.6%) | 1,799 (9.3%) | 983 (9.9%) | 21 (12%) |

© 2023 Fei-Zhang DJ et al. *JAMA Network Open.*

| | | Housing-Transport SVI Subscore | | | | |
|---|---|---|---|---|---|---|
| Characteristic | N | 0.000-0.199, N = 82 (0.2%) | 0.200-0.399, N = 7440 (20%) | 0.400-0.599, N = 19399 (52%) | 0.600-0.799, N = 9948 (27%) | 0.800-0.999, N = 174 (0.5%) |
| Intracranial/spinal Germ Cell Tumors | | 4 (4.9%) | 173 (2.3%) | 535 (2.8%) | 316 (3.2%) | 5 (2.9%) |
| Kaposi Sarcoma | | 0 (0%) | 1 (<0.1%) | 4 (<0.1%) | 8 (<0.1%) | 0 (0%) |
| Malignant Melanomas | | 16 (20%) | 670 (9.0%) | 1,413 (7.3%) | 703 (7.1%) | 10 (5.7%) |
| Nasopharyngeal Carcinomas | | 0 (0%) | 36 (0.5%) | 180 (0.9%) | 105 (1.1%) | 1 (0.6%) |
| Neuroblastomas & Ganglioneuroblastomas | | 1 (1.2%) | 75 (1.0%) | 188 (1.0%) | 62 (0.6%) | 2 (1.1%) |
| Non-Hodgkin Lymphoma | | 3 (3.7%) | 292 (3.9%) | 744 (3.8%) | 376 (3.8%) | 12 (6.9%) |
| Osteosarcomas | | 2 (2.4%) | 89 (1.2%) | 292 (1.5%) | 145 (1.5%) | 4 (2.3%) |
| Other-NOS Gliomas | | 2 (2.4%) | 588 (7.9%) | 1,576 (8.1%) | 777 (7.8%) | 13 (7.5%) |
| Other or Unspecified Carcinomas | | 1 (1.2%) | 170 (2.3%) | 474 (2.4%) | 225 (2.3%) | 4 (2.3%) |
| Other or Unspecified Intracranial/spinal CNS Tumors | | 2 (2.4%) | 104 (1.4%) | 288 (1.5%) | 128 (1.3%) | 1 (0.6%) |
| Other or Unspecified Leukemias | | 0 (0%) | 7 (<0.1%) | 28 (0.1%) | 21 (0.2%) | 0 (0%) |
| Other or Unspecified Lymphomas | | 0 (0%) | 40 (0.5%) | 110 (0.6%) | 85 (0.9%) | 3 (1.7%) |
| Other or Unspecified Malignant Bone Tumors | | 0 (0%) | 38 (0.5%) | 95 (0.5%) | 61 (0.6%) | 1 (0.6%) |
| Other or Unspecified Malignant Neoplasms | | 1 (1.2%) | 14 (0.2%) | 20 (0.1%) | 15 (0.2%) | 1 (0.6%) |

© 2023 Fei-Zhang DJ et al. *JAMA Network Open.*

| | | Housing-Transport SVI Subscore | | | | |
|---|---|---|---|---|---|---|
| Characteristic | N | 0.000-0.199, N = 82 (0.2%) | 0.200-0.399, N = 7440 (20%) | 0.400-0.599, N = 19399 (52%) | 0.600-0.799, N = 9948 (27%) | 0.800-0.999, N = 174 (0.5%) |
| Other or Unspecified PNS Tumors | | 0 (0%) | 20 (0.3%) | 31 (0.2%) | 18 (0.2%) | 1 (0.6%) |
| Other or Unspecified Soft Tissue Sarcomas | | 3 (3.7%) | 273 (3.7%) | 735 (3.8%) | 386 (3.9%) | 6 (3.4%) |
| Retinoblastoma | | 3 (3.7%) | 317 (4.3%) | 1,044 (5.4%) | 602 (6.1%) | 10 (5.7%) |
| Rhabdomyosarcomas | | 0 (0%) | 210 (2.8%) | 619 (3.2%) | 289 (2.9%) | 6 (3.4%) |
| Skin Carcinomas | | 0 (0%) | 6 (<0.1%) | 23 (0.1%) | 19 (0.2%) | 0 (0%) |
| Thyroid Carcinomas | | 6 (7.3%) | 884 (12%) | 2,233 (12%) | 1,162 (12%) | 17 (9.8%) |
| Unknown | | 0 (0%) | 14 (0.2%) | 44 (0.2%) | 32 (0.3%) | 0 (0%) |
| **Vital Status on Last Follow-up** | 37,043 | | | | | |
| Alive | | 73 (89%) | 5,835 (78%) | 14,948 (77%) | 7,677 (77%) | 120 (69%) |
| Dead | | 9 (11%) | 1,605 (22%) | 4,451 (23%) | 2,271 (23%) | 54 (31%) |

© 2023 Fei-Zhang DJ et al. *JAMA Network Open.*

**eTable 2.** ICCC Disease Class Trends in Months Under Surveillance by Relative SVI Percentile

| | | Relative Total SVI %-Tile | | | | | |
|---|---|---|---|---|---|---|---|
| **ICCC Class[1]** | | **<20** | **20-39.99** | **40-59.99** | **60-79.99** | **80-99.99** | **p-value** |
| Astrocytomas | **Sample Size** | 1,744 | 1,743 | 1,743 | 1,743 | 1,743 | |
| | **Months Surveyed** | | | | | | <0.001 |
| | Mean (SD) | 140 (129) | 146 (130) | 122 (118) | 118 (110) | 93 (83) | |
| | Median (IQR) | 101 (28-210) | 112 (29-231) | 86 (25-178) | 92 (24-180) | 73 (19-149) | |
| | Range | 0-503 | 0-503 | 0-503 | 0-500 | 0-485 | |
| | **Total SVI Score** | | | | | | |
| | Mean (SD) | 0.30 (0.05) | 0.41 (0.02) | 0.48 (0.02) | 0.58 (0.04) | 0.69 (0.06) | |
| | Range | 0.00-0.37 | 0.37-0.44 | 0.44-0.51 | 0.51-0.64 | 0.64-0.95 | |
| | **% Alive/Lost on Last Follow-up** | 0.760 | 0.764 | 0.766 | 0.749 | 0.770 | |
| | **% Dead on Last Follow-up** | 0.240 | 0.236 | 0.234 | 0.251 | 0.230 | |
| Intracranial/spinal Embryonal Tumors | **Sample Size** | 690 | 690 | 690 | 690 | 690 | |
| | **Months Surveyed** | | | | | | <0.001 |
| | Mean (SD) | 111 (121) | 100 (114) | 88 (95) | 89 (99) | 78 (86) | |
| | Median (IQR) | 67 (14-168) | 50 (13-162) | 52 (12-134) | 53 (11-142) | 42 (11-120) | |
| | Range | 0-487 | 0-492 | 0-487 | 0-488 | 0-486 | |
| | **Total SVI Score** | | | | | | |
| | Mean (SD) | 0.31 (0.06) | 0.42 (0.02) | 0.50 (0.02) | 0.60 (0.03) | 0.70 (0.07) | |
| | Range | 0.00-0.40 | 0.40-0.46 | 0.46-0.54 | 0.54-0.64 | 0.64-0.95 | |
| | **% Alive/Lost on Last Follow-up** | 0.562 | 0.523 | 0.572 | 0.514 | 0.614 | |

© 2023 Fei-Zhang DJ et al. *JAMA Network Open.*

| | | Relative Total SVI %-Tile | | | | | |
|---|---|---|---|---|---|---|---|
| **ICCC Class[1]** | | **<20** | **20-39.99** | **40-59.99** | **60-79.99** | **80-99.99** | **p-value** |
| | **% Dead on Last Follow-up** | 0.438 | 0.477 | 0.428 | 0.486 | 0.386 | |
| Ependymomas & Choroid Plexus Tumors | **Sample Size** | 301 | 301 | 300 | 300 | 300 | |
| | **Months Surveyed** | | | | | | <0.001 |
| | Mean (SD) | 101 (107) | 112 (115) | 101 (95) | 94 (91) | 76 (72) | |
| | Median (IQR) | 60 (22-160) | 65 (20-178) | 71 (26-150) | 62 (22-145) | 55 (18-118) | |
| | Range | 0-495 | 0-493 | 0-422 | 0-486 | 0-407 | |
| | **Total SVI Score** | | | | | | |
| | Mean (SD) | 0.31 (0.06) | 0.42 (0.02) | 0.49 (0.03) | 0.60 (0.03) | 0.69 (0.07) | |
| | Range | 0.00-0.39 | 0.39-0.44 | 0.45-0.55 | 0.55-0.64 | 0.64-0.93 | |
| | **% Alive/Lost on Last Follow-up** | 0.66 | 0.55 | 0.70 | 0.63 | 0.69 | |
| | **% Dead on Last Follow-up** | 0.34 | 0.45 | 0.30 | 0.37 | 0.31 | |
| Intracranial/spinal Germ Cell Tumors | **Sample Size** | 207 | 207 | 207 | 206 | 206 | |
| | **Months Surveyed** | | | | | | <0.001 |
| | Mean (SD) | 137 (117) | 132 (112) | 112 (103) | 105 (82) | 102 (86) | |
| | Median (IQR) | 107 (38-205) | 110 (42-196) | 91 (27-171) | 98 (34-153) | 80 (32-157) | |
| | Range | 0-456 | 0-463 | 0-458 | 0-436 | 0-443 | |
| | **Total SVI Score** | | | | | | |
| | Mean (SD) | 0.32 (0.06) | 0.44 (0.02) | 0.50 (0.02) | 0.61 (0.03) | 0.69 (0.06) | |
| | Range | 0.15-0.40 | 0.40-0.47 | 0.47-0.55 | 0.55-0.64 | 0.64-0.93 | |

© 2023 Fei-Zhang DJ et al. *JAMA Network Open.*

| | | Relative Total SVI %-Tile | | | | | |
|---|---|---|---|---|---|---|---|
| **ICCC Class[1]** | | **<20** | **20-39.99** | **40-59.99** | **60-79.99** | **80-99.99** | **p-value** |
| | **% Alive/Lost on Last Follow-up** | 0.768 | 0.744 | 0.778 | 0.806 | 0.825 | |
| | **% Dead on Last Follow-up** | 0.232 | 0.256 | 0.222 | 0.194 | 0.175 | |
| Hodgkin Lymphomas | **Sample Size** | 343 | 343 | 343 | 343 | 342 | |
| | **Months Surveyed** | | | | | | <0.001 |
| | Mean (SD) | 216 (142) | 222 (147) | 184 (134) | 172 (129) | 127 (94) | |
| | Median (IQR) | 199 (90-339) | 201 (88-336) | 157 (72-290) | 142 (78-239) | 116 (52-183) | |
| | Range | 0-494 | 0-497 | 0-498 | 0-501 | 0-487 | |
| | **Total SVI Score** | | | | | | |
| | Mean (SD) | 0.30 (0.06) | 0.41 (0.02) | 0.48 (0.03) | 0.58 (0.03) | 0.69 (0.07) | |
| | Range | 0.03-0.37 | 0.37-0.44 | 0.44-0.52 | 0.52-0.64 | 0.64-0.95 | |
| | **% Alive/Lost on Last Follow-up** | 0.863 | 0.860 | 0.866 | 0.872 | 0.953 | |
| | **% Dead on Last Follow-up** | 0.137 | 0.140 | 0.134 | 0.128 | 0.047 | |
| Non-Hodgkin Lymphomas | **Sample Size** | 286 | 286 | 285 | 285 | 285 | |
| | **Months Surveyed** | | | | | | <0.001 |
| | Mean (SD) | 141 (126) | 126 (123) | 125 (114) | 117 (99) | 97 (88) | |
| | Median (IQR) | 104 (34-200) | 87 (33-165) | 103 (38-181) | 106 (43-166) | 74 (28-148) | |
| | Range | 0-491 | 0-477 | 0-498 | 0-494 | 0-467 | |
| | **Total SVI Score** | | | | | | |
| | Mean (SD) | 0.31 (0.06) | 0.42 (0.02) | 0.49 (0.02) | 0.59 (0.03) | 0.70 (0.08) | |

© 2023 Fei-Zhang DJ et al. *JAMA Network Open.*

| | | Relative Total SVI %-Tile | | | | | |
|---|---|---|---|---|---|---|---|
| **ICCC Class[1]** | | **<20** | **20-39.99** | **40-59.99** | **60-79.99** | **80-99.99** | **p-value** |
| | Range | 0.03-0.40 | 0.40-0.45 | 0.45-0.54 | 0.54-0.64 | 0.64-0.95 | |
| | **% Alive/Lost on Last Follow-up** | 0.86 | 0.80 | 0.93 | 0.86 | 0.86 | |
| | **% Dead on Last Follow-up** | 0.14 | 0.20 | 0.07 | 0.14 | 0.14 | |
| Malignant Melanomas | **Sample Size** | 563 | 563 | 562 | 562 | 562 | |
| | **Months Surveyed** | | | | | | <0.001 |
| | Mean (SD) | 170 (128) | 178 (133) | 168 (123) | 133 (109) | 129 (88) | |
| | Median (IQR) | 143 (63-246) | 158 (66-261) | 146 (77-228) | 114 (50-179) | 138 (53-183) | |
| | Range | 0-489 | 0-502 | 0-502 | 0-497 | 0-473 | |
| | **Total SVI Score** | | | | | | |
| | Mean (SD) | 0.29 (0.06) | 0.39 (0.02) | 0.46 (0.02) | 0.54 (0.03) | 0.68 (0.06) | |
| | Range | 0.00-0.36 | 0.36-0.43 | 0.43-0.50 | 0.50-0.61 | 0.61-0.95 | |
| | **% Alive/Lost on Last Follow-up** | 0.893 | 0.920 | 0.888 | 0.913 | 0.925 | |
| | **% Dead on Last Follow-up** | 0.107 | 0.080 | 0.112 | 0.087 | 0.075 | |
| Other-NOS Gliomas | **Sample Size** | 592 | 591 | 591 | 591 | 591 | |
| | **Months Surveyed** | | | | | | <0.001 |
| | Mean (SD) | 110 (117) | 109 (127) | 96 (119) | 81 (105) | 67 (79) | |
| | Median (IQR) | 74 (12-174) | 44 (11-180) | 38 (10-146) | 28 (10-126) | 26 (8-117) | |
| | Range | 0-503 | 0-503 | 0-497 | 0-501 | 0-498 | |
| | **Total SVI Score** | | | | | | |

© 2023 Fei-Zhang DJ et al. *JAMA Network Open.*

| | | Relative Total SVI %-Tile | | | | | |
|---|---|---|---|---|---|---|---|
| **ICCC Class[1]** | | **<20** | **20-39.99** | **40-59.99** | **60-79.99** | **80-99.99** | **p-value** |
| | Mean (SD) | 0.31 (0.06) | 0.41 (0.02) | 0.47 (0.02) | 0.57 (0.04) | 0.68 (0.06) | |
| | Range | 0.00-0.39 | 0.39-0.44 | 0.44-0.51 | 0.51-0.63 | 0.63-0.93 | |
| | **% Alive/Lost on Last Follow-up** | 0.610 | 0.525 | 0.558 | 0.531 | 0.536 | |
| | **% Dead on Last Follow-up** | 0.390 | 0.475 | 0.442 | 0.469 | 0.464 | |
| Other or Unspecified Soft Tissue Sarcomas | **Sample Size** | 281 | 281 | 281 | 280 | 280 | |
| | **Months Surveyed** | | | | | | <0.001 |
| | Mean (SD) | 167 (136) | 175 (135) | 154 (138) | 139 (123) | 105 (94) | |
| | Median (IQR) | 149 (52-251) | 154 (59-276) | 111 (48-222) | 118 (34-197) | 79 (30-154) | |
| | Range | 0-499 | 0-499 | 0-501 | 0-499 | 0-502 | |
| | **Total SVI Score** | | | | | | |
| | Mean (SD) | 0.32 (0.06) | 0.42 (0.02) | 0.50 (0.02) | 0.59 (0.03) | 0.69 (0.06) | |
| | Range | 0.13-0.40 | 0.40-0.45 | 0.46-0.55 | 0.55-0.64 | 0.64-0.95 | |
| | **% Alive/Lost on Last Follow-up** | 0.851 | 0.854 | 0.854 | 0.814 | 0.875 | |
| | **% Dead on Last Follow-up** | 0.149 | 0.146 | 0.146 | 0.186 | 0.125 | |
| Retinoblastomas | **Sample Size** | 396 | 395 | 395 | 395 | 395 | |
| | **Months Surveyed** | | | | | | <0.001 |
| | Mean (SD) | 180 (133) | 191 (135) | 144 (126) | 135 (108) | 113 (95) | |
| | Median (IQR) | 163 (67-269) | 180 (67-295) | 107 (47-200) | 123 (52-176) | 85 (32-186) | |

© 2023 Fei-Zhang DJ et al. *JAMA Network Open.*

| | | Relative Total SVI %-Tile | | | | | |
|---|---|---|---|---|---|---|---|
| ICCC Class[1] | | <20 | 20-39.99 | 40-59.99 | 60-79.99 | 80-99.99 | p-value |
| | Range | 0-502 | 0-501 | 0-498 | 1-496 | 0-482 | |
| | **Total SVI Score** | | | | | | |
| | Mean (SD) | 0.32 (0.06) | 0.43 (0.02) | 0.51 (0.03) | 0.61 (0.03) | 0.70 (0.07) | |
| | Range | 0.00-0.40 | 0.40-0.47 | 0.47-0.56 | 0.56-0.64 | 0.64-0.95 | |
| | **% Alive/Lost on Last Follow-up** | 0.929 | 0.947 | 0.944 | 0.939 | 0.944 | |
| | **% Dead on Last Follow-up** | 0.071 | 0.053 | 0.056 | 0.061 | 0.056 | |
| Rhabdomyosarcomas | **Sample Size** | 225 | 225 | 225 | 225 | 224 | |
| | **Months Surveyed** | | | | | | <0.001 |
| | Mean (SD) | 133 (126) | 137 (140) | 98 (115) | 124 (121) | 87 (86) | |
| | Median (IQR) | 92 (28-195) | 76 (21-218) | 46 (18-140) | 84 (24-183) | 54 (17-143) | |
| | Range | 0-500 | 0-503 | 1-489 | 0-502 | 0-389 | |
| | **Total SVI Score** | | | | | | |
| | Mean (SD) | 0.31 (0.07) | 0.42 (0.02) | 0.50 (0.02) | 0.59 (0.03) | 0.70 (0.06) | |
| | Range | 0.00-0.40 | 0.40-0.45 | 0.45-0.53 | 0.53-0.64 | 0.64-0.93 | |
| | **% Alive/Lost on Last Follow-up** | 0.70 | 0.64 | 0.60 | 0.69 | 0.67 | |
| | **% Dead on Last Follow-up** | 0.30 | 0.36 | 0.40 | 0.31 | 0.33 | |
| Thyroid Carcinomas | **Sample Size** | 861 | 861 | 860 | 860 | 860 | |
| | **Months Surveyed** | | | | | | <0.001 |
| | Mean (SD) | 156 (133) | 167 (136) | 138 (125) | 138 (118) | 98 (81) | |

© 2023 Fei-Zhang DJ et al. *JAMA Network Open.*

| | | Relative Total SVI %-Tile | | | | | |
|---|---|---|---|---|---|---|---|
| ICCC Class[1] | | <20 | 20-39.99 | 40-59.99 | 60-79.99 | 80-99.99 | p-value |
| | Median (IQR) | 119 (51-218) | 124 (57-252) | 100 (43-190) | 112 (48-186) | 82 (33-143) | |
| | Range | 0-503 | 0-502 | 0-500 | 0-496 | 0-463 | |
| | **Total SVI Score** | | | | | | |
| | Mean (SD) | 0.30 (0.06) | 0.42 (0.02) | 0.49 (0.02) | 0.58 (0.04) | 0.69 (0.06) | |
| | Range | 0.00-0.37 | 0.37-0.45 | 0.45-0.51 | 0.51-0.64 | 0.64-0.93 | |
| | **% Alive/Lost on Last Follow-up** | 0.979 | 0.976 | 0.967 | 0.970 | 0.976 | |
| | **% Dead on Last Follow-up** | 0.021 | 0.024 | 0.033 | 0.030 | 0.024 | |

[1]By International Classification of Childhood Cancer

| | | Relative Socioeconomic-SVI %-Tile | | | | | |
|---|---|---|---|---|---|---|---|
| ICCC Class[1] | | <20 | 20-39.99 | 40-59.99 | 60-79.99 | 80-99.99 | p-value |
| Astrocytomas | **Sample Size** | 1,744 | 1,743 | 1,743 | 1,743 | 1,743 | |
| | **Months Surveyed** | | | | | | <0.001 |
| | Mean (SD) | 137 (126) | 152 (135) | 124 (116) | 105 (95) | 101 (99) | |
| | Median (IQR) | 98 (29-208) | 121 (31-244) | 93 (26-179) | 84 (21-167) | 74 (20-156) | |
| | Range | 0-503 | 0-503 | 0-501 | 0-499 | 0-500 | |
| | **Socioeconomic SVI Subscore** | | | | | | |
| | Mean (SD) | 0.27 (0.04) | 0.37 (0.02) | 0.44 (0.02) | 0.56 (0.04) | 0.70 (0.07) | |
| | Range | 0.00-0.33 | 0.33-0.41 | 0.41-0.48 | 0.48-0.61 | 0.61-0.96 | |
| | **% Alive/Lost on Last Follow-up** | 0.755 | 0.752 | 0.780 | 0.770 | 0.752 | |

© 2023 Fei-Zhang DJ et al. *JAMA Network Open.*

| | | Relative Socioeconomic-SVI %-Tile | | | | | |
|---|---|---|---|---|---|---|---|
| **ICCC Class[1]** | | **<20** | **20-39.99** | **40-59.99** | **60-79.99** | **80-99.99** | **p-value** |
| | **% Dead on Last Follow-up** | 0.245 | 0.248 | 0.220 | 0.230 | 0.248 | |
| Intracranial/spinal Embryonal Tumors | **Sample Size** | 690 | 690 | 690 | 690 | 690 | |
| | **Months Surveyed** | | | | | | <0.001 |
| | Mean (SD) | 107 (120) | 105 (113) | 86 (97) | 85 (94) | 82 (93) | |
| | Median (IQR) | 58 (13-168) | 63 (14-172) | 49 (14-130) | 47 (10-141) | 46 (12-123) | |
| | Range | 0-490 | 0-484 | 0-492 | 0-488 | 0-486 | |
| | **Socioeconomic SVI Subscore** | | | | | | |
| | Mean (SD) | 0.28 (0.04) | 0.37 (0.02) | 0.45 (0.03) | 0.58 (0.02) | 0.70 (0.07) | |
| | Range | 0.00-0.33 | 0.33-0.42 | 0.42-0.53 | 0.53-0.61 | 0.61-0.94 | |
| | **% Alive/Lost on Last Follow-up** | 0.529 | 0.536 | 0.577 | 0.554 | 0.591 | |
| | **% Dead on Last Follow-up** | 0.471 | 0.464 | 0.423 | 0.446 | 0.409 | |
| Ependymomas & Choroid Plexus Tumors | **Sample Size** | 301 | 301 | 300 | 300 | 300 | |
| | **Months Surveyed** | | | | | | <0.001 |
| | Mean (SD) | 114 (113) | 104 (112) | 96 (91) | 84 (83) | 86 (84) | |
| | Median (IQR) | 70 (25-168) | 60 (20-158) | 70 (23-149) | 56 (16-130) | 59 (24-126) | |
| | Range | 0-495 | 0-486 | 0-454 | 0-422 | 0-486 | |
| | **Socioeconomic SVI Subscore** | | | | | | |
| | Mean (SD) | 0.27 (0.05) | 0.37 (0.02) | 0.45 (0.03) | 0.58 (0.02) | 0.69 (0.07) | |

© 2023 Fei-Zhang DJ et al. *JAMA Network Open.*

| | | Relative Socioeconomic-SVI %-Tile | | | | | |
|---|---|---|---|---|---|---|---|
| **ICCC Class[1]** | | **<20** | **20-39.99** | **40-59.99** | **60-79.99** | **80-99.99** | **p-value** |
| | Range | 0.00-0.33 | 0.33-0.42 | 0.42-0.53 | 0.53-0.61 | 0.61-0.93 | |
| | **% Alive/Lost on Last Follow-up** | 0.598 | 0.635 | 0.667 | 0.687 | 0.633 | |
| | **% Dead on Last Follow-up** | 0.402 | 0.365 | 0.333 | 0.313 | 0.367 | |
| Intracranial/spinal Germ Cell Tumors | **Sample Size** | 207 | 207 | 207 | 206 | 206 | |
| | **Months Surveyed** | | | | | | <0.001 |
| | Mean (SD) | 137 (117) | 142 (115) | 109 (101) | 97 (76) | 105 (89) | |
| | Median (IQR) | 117 (39-208) | 120 (41-215) | 90 (26-161) | 83 (33-149) | 83 (32-156) | |
| | Range | 0-463 | 0-461 | 0-452 | 0-295 | 0-443 | |
| | **Socioeconomic SVI Subscore** | | | | | | |
| | Mean (SD) | 0.28 (0.04) | 0.37 (0.03) | 0.46 (0.03) | 0.58 (0.02) | 0.69 (0.06) | |
| | Range | 0.14-0.33 | 0.33-0.42 | 0.42-0.54 | 0.54-0.60 | 0.60-0.90 | |
| | **% Alive/Lost on Last Follow-up** | 0.729 | 0.754 | 0.807 | 0.806 | 0.825 | |
| | **% Dead on Last Follow-up** | 0.271 | 0.246 | 0.193 | 0.194 | 0.175 | |
| Hodgkin Lymphomas | **Sample Size** | 343 | 343 | 343 | 343 | 342 | |
| | **Months Surveyed** | | | | | | <0.001 |
| | Mean (SD) | 214 (146) | 228 (141) | 184 (137) | 150 (110) | 147 (116) | |
| | Median (IQR) | 191 (86-332) | 224 (99-345) | 153 (74-288) | 130 (64-205) | 129 (62-188) | |
| | Range | 0-498 | 0-494 | 0-497 | 0-501 | 0-497 | |

© 2023 Fei-Zhang DJ et al. *JAMA Network Open.*

| | | Relative Socioeconomic-SVI %-Tile | | | | | |
|---|---|---|---|---|---|---|---|
| **ICCC Class[1]** | | **<20** | **20-39.99** | **40-59.99** | **60-79.99** | **80-99.99** | **p-value** |
| | **Socioeconomic SVI Subscore** | | | | | | |
| | Mean (SD) | 0.28 (0.05) | 0.37 (0.02) | 0.44 (0.02) | 0.57 (0.03) | 0.70 (0.07) | |
| | Range | 0.08-0.35 | 0.35-0.41 | 0.41-0.50 | 0.51-0.63 | 0.63-0.96 | |
| | **% Alive/Lost on Last Follow-up** | 0.88 | 0.83 | 0.86 | 0.94 | 0.91 | |
| | **% Dead on Last Follow-up** | 0.12 | 0.17 | 0.14 | 0.06 | 0.09 | |
| Non-Hodgkin Lymphomas | **Sample Size** | 286 | 286 | 285 | 285 | 285 | |
| | **Months Surveyed** | | | | | | <0.001 |
| | Mean (SD) | 131 (122) | 151 (136) | 111 (96) | 110 (94) | 103 (96) | |
| | Median (IQR) | 95 (32-188) | 114 (34-228) | 94 (39-154) | 86 (37-162) | 79 (29-155) | |
| | Range | 0-479 | 0-498 | 0-458 | 0-489 | 0-494 | |
| | **Socioeconomic SVI Subscore** | | | | | | |
| | Mean (SD) | 0.28 (0.05) | 0.38 (0.02) | 0.46 (0.03) | 0.57 (0.03) | 0.70 (0.07) | |
| | Range | 0.11-0.35 | 0.35-0.42 | 0.42-0.52 | 0.52-0.60 | 0.60-0.95 | |
| | **% Alive/Lost on Last Follow-up** | 0.836 | 0.839 | 0.912 | 0.870 | 0.849 | |
| | **% Dead on Last Follow-up** | 0.164 | 0.161 | 0.088 | 0.130 | 0.151 | |
| Malignant Melanomas | **Sample Size** | 563 | 563 | 562 | 562 | 562 | |
| | **Months Surveyed** | | | | | | <0.001 |
| | Mean (SD) | 173 (133) | 191 (137) | 145 (110) | 139 (101) | 130 (98) | |

© 2023 Fei-Zhang DJ et al. *JAMA Network Open.*

| | | Relative Socioeconomic-SVI %-Tile | | | | | |
|---|---|---|---|---|---|---|---|
| **ICCC Class[1]** | | **<20** | **20-39.99** | **40-59.99** | **60-79.99** | **80-99.99** | **p-value** |
| | Median (IQR) | 149 (64-250) | 162 (79-286) | 130 (59-187) | 131 (60-184) | 128 (45-184) | |
| | Range | 0-496 | 0-502 | 0-502 | 0-489 | 0-497 | |
| | **Socioeconomic SVI Subscore** | | | | | | |
| | Mean (SD) | 0.26 (0.04) | 0.36 (0.02) | 0.42 (0.02) | 0.52 (0.05) | 0.67 (0.07) | |
| | Range | 0.00-0.32 | 0.32-0.38 | 0.38-0.45 | 0.45-0.60 | 0.60-0.94 | |
| | **% Alive/Lost on Last Follow-up** | 0.902 | 0.895 | 0.915 | 0.907 | 0.920 | |
| | **% Dead on Last Follow-up** | 0.098 | 0.105 | 0.085 | 0.093 | 0.080 | |
| Other-NOS Gliomas | **Sample Size** | 592 | 591 | 591 | 591 | 591 | |
| | **Months Surveyed** | | | | | | <0.001 |
| | Mean (SD) | 114 (125) | 108 (127) | 87 (108) | 80 (95) | 74 (96) | |
| | Median (IQR) | 67 (11-182) | 44 (11-182) | 32 (11-133) | 35 (10-133) | 26 (9-116) | |
| | Range | 0-503 | 0-503 | 0-482 | 0-494 | 0-501 | |
| | **Socioeconomic SVI Subscore** | | | | | | |
| | Mean (SD) | 0.27 (0.04) | 0.36 (0.02) | 0.44 (0.02) | 0.56 (0.04) | 0.69 (0.07) | |
| | Range | 0.00-0.33 | 0.33-0.40 | 0.40-0.47 | 0.47-0.60 | 0.60-0.93 | |
| | **% Alive/Lost on Last Follow-up** | 0.579 | 0.528 | 0.557 | 0.582 | 0.514 | |
| | **% Dead on Last Follow-up** | 0.421 | 0.472 | 0.443 | 0.418 | 0.486 | |

© 2023 Fei-Zhang DJ et al. *JAMA Network Open.*

| | | Relative Socioeconomic-SVI %-Tile | | | | | |
|---|---|---|---|---|---|---|---|
| **ICCC Class[1]** | | **<20** | **20-39.99** | **40-59.99** | **60-79.99** | **80-99.99** | **p-value** |
| Other or Unspecified Soft Tissue Sarcomas | **Sample Size** | 281 | 281 | 281 | 280 | 280 | |
| | **Months Surveyed** | | | | | | <0.001 |
| | Mean (SD) | 189 (144) | 178 (140) | 133 (119) | 118 (102) | 122 (117) | |
| | Median (IQR) | 164 (68-297) | 152 (54-294) | 102 (40-177) | 88 (26-192) | 90 (32-172) | |
| | Range | 0-499 | 0-501 | 0-499 | 0-472 | 0-502 | |
| | **Socioeconomic SVI Subscore** | | | | | | |
| | Mean (SD) | 0.29 (0.05) | 0.38 (0.02) | 0.45 (0.03) | 0.58 (0.03) | 0.69 (0.06) | |
| | Range | 0.14-0.35 | 0.35-0.42 | 0.42-0.54 | 0.54-0.61 | 0.61-0.94 | |
| | **% Alive/Lost on Last Follow-up** | 0.879 | 0.822 | 0.872 | 0.814 | 0.861 | |
| | **% Dead on Last Follow-up** | 0.121 | 0.178 | 0.128 | 0.186 | 0.139 | |
| Retinoblastomas | **Sample Size** | 396 | 395 | 395 | 395 | 395 | |
| | **Months Surveyed** | | | | | | <0.001 |
| | Mean (SD) | 190 (135) | 196 (139) | 129 (115) | 124 (86) | 124 (115) | |
| | Median (IQR) | 184 (65-278) | 180 (76-299) | 98 (44-176) | 119 (51-188) | 101 (35-181) | |
| | Range | 0-498 | 0-502 | 0-499 | 0-448 | 0-496 | |
| | **Socioeconomic SVI Subscore** | | | | | | |
| | Mean (SD) | 0.28 (0.05) | 0.38 (0.03) | 0.47 (0.04) | 0.59 (0.02) | 0.71 (0.07) | |
| | Range | 0.00-0.35 | 0.35-0.43 | 0.43-0.54 | 0.54-0.62 | 0.62-0.95 | |

© 2023 Fei-Zhang DJ et al. *JAMA Network Open.*

| | | Relative Socioeconomic-SVI %-Tile | | | | | |
|---|---|---|---|---|---|---|---|
| ICCC Class[1] | | <20 | 20-39.99 | 40-59.99 | 60-79.99 | 80-99.99 | p-value |
| | **% Alive/Lost on Last Follow-up** | 0.909 | 0.952 | 0.952 | 0.959 | 0.932 | |
| | **% Dead on Last Follow-up** | 0.091 | 0.048 | 0.048 | 0.041 | 0.068 | |
| Rhabdomyosarcomas | **Sample Size** | 225 | 225 | 225 | 225 | 224 | |
| | **Months Surveyed** | | | | | | <0.001 |
| | Mean (SD) | 126 (127) | 138 (141) | 110 (119) | 105 (103) | 100 (103) | |
| | Median (IQR) | 76 (25-183) | 80 (20-228) | 65 (21-166) | 70 (19-170) | 58 (22-160) | |
| | Range | 0-475 | 0-503 | 1-501 | 0-502 | 0-426 | |
| | **Socioeconomic SVI Subscore** | | | | | | |
| | Mean (SD) | 0.28 (0.06) | 0.38 (0.02) | 0.45 (0.02) | 0.58 (0.03) | 0.70 (0.07) | |
| | Range | 0.00-0.35 | 0.35-0.42 | 0.42-0.52 | 0.52-0.63 | 0.63-0.98 | |
| | **% Alive/Lost on Last Follow-up** | 0.653 | 0.640 | 0.636 | 0.711 | 0.652 | |
| | **% Dead on Last Follow-up** | 0.347 | 0.360 | 0.364 | 0.289 | 0.348 | |
| Thyroid Carcinomas | **Sample Size** | 861 | 861 | 860 | 860 | 860 | |
| | **Months Surveyed** | | | | | | <0.001 |
| | Mean (SD) | 161 (135) | 174 (137) | 132 (123) | 113 (94) | 117 (106) | |
| | Median (IQR) | 125 (53-238) | 141 (58-266) | 92 (42-180) | 91 (42-161) | 93 (37-163) | |
| | Range | 0-503 | 0-500 | 0-500 | 0-496 | 0-489 | |
| | **Socioeconomic SVI Subscore** | | | | | | |

© 2023 Fei-Zhang DJ et al. *JAMA Network Open.*

| | | Relative Socioeconomic-SVI %-Tile | | | | | |
|---|---|---|---|---|---|---|---|
| **ICCC Class[1]** | | **<20** | **20-39.99** | **40-59.99** | **60-79.99** | **80-99.99** | **p-value** |
| | Mean (SD) | 0.27 (0.05) | 0.37 (0.02) | 0.44 (0.02) | 0.57 (0.03) | 0.69 (0.07) | |
| | Range | 0.00-0.33 | 0.33-0.42 | 0.42-0.48 | 0.48-0.60 | 0.60-0.93 | |
| | **% Alive/Lost on Last Follow-up** | 0.973 | 0.966 | 0.977 | 0.979 | 0.972 | |
| | **% Dead on Last Follow-up** | 0.027 | 0.034 | 0.023 | 0.021 | 0.028 | |
| [1]By International Classification of Childhood Cancer | | | | | | | |

| | | Relative Minority-Language-SVI %-Tile | | | | | |
|---|---|---|---|---|---|---|---|
| **ICCC Class[1]** | | **<20** | **20-39.99** | **40-59.99** | **60-79.99** | **80-99.99** | **p-value** |
| Astrocytomas | **Sample Size** | 1,744 | 1,743 | 1,743 | 1,743 | 1,743 | |
| | **Months Surveyed** | | | | | | <0.001 |
| | Mean (SD) | 136 (128) | 132 (122) | 134 (125) | 103 (97) | 116 (104) | |
| | Median (IQR) | 96 (29-196) | 96 (27-197) | 98 (25-198) | 81 (24-154) | 95 (22-183) | |
| | Range | 0-503 | 0-500 | 0-503 | 0-503 | 0-503 | |
| | **Minority-Language SVI Subscore** | | | | | | |
| | Mean (SD) | 0.28 (0.10) | 0.46 (0.03) | 0.58 (0.04) | 0.74 (0.03) | 0.83 (0.02) | |
| | Range | 0.00-0.40 | 0.40-0.50 | 0.50-0.67 | 0.67-0.79 | 0.79-0.95 | |
| | **% Alive/Lost on Last Follow-up** | 0.764 | 0.745 | 0.760 | 0.790 | 0.750 | |
| | **% Dead on Last Follow-up** | 0.236 | 0.255 | 0.240 | 0.210 | 0.250 | |
| Intracranial/spinal Embryonal Tumors | **Sample Size** | 690 | 690 | 690 | 690 | 690 | |
| | **Months Surveyed** | | | | | | <0.001 |

© 2023 Fei-Zhang DJ et al. *JAMA Network Open.*

| | | Relative Minority-Language-SVI %-Tile | | | | | |
|---|---|---|---|---|---|---|---|
| **ICCC Class[1]** | | **<20** | **20-39.99** | **40-59.99** | **60-79.99** | **80-99.99** | **p-value** |
| | Mean (SD) | 98 (107) | 103 (117) | 95 (111) | 87 (95) | 82 (87) | |
| | Median (IQR) | 57 (15-148) | 54 (13-166) | 47 (11-144) | 55 (13-133) | 45 (11-141) | |
| | Range | 0-492 | 0-490 | 0-488 | 0-463 | 0-446 | |
| | **Minority-Language SVI Subscore** | | | | | | |
| | Mean (SD) | 0.31 (0.10) | 0.48 (0.04) | 0.63 (0.05) | 0.77 (0.02) | 0.84 (0.02) | |
| | Range | 0.03-0.42 | 0.42-0.55 | 0.55-0.72 | 0.72-0.81 | 0.81-0.95 | |
| | **% Alive/Lost on Last Follow-up** | 0.575 | 0.543 | 0.526 | 0.568 | 0.574 | |
| | **% Dead on Last Follow-up** | 0.425 | 0.457 | 0.474 | 0.432 | 0.426 | |
| Ependymomas & Choroid Plexus Tumors | **Sample Size** | 301 | 301 | 300 | 300 | 300 | |
| | **Months Surveyed** | | | | | | 0.013 |
| | Mean (SD) | 103 (103) | 101 (105) | 98 (107) | 100 (93) | 81 (78) | |
| | Median (IQR) | 62 (23-158) | 60 (26-138) | 61 (18-140) | 70 (26-156) | 57 (16-128) | |
| | Range | 0-495 | 0-486 | 0-493 | 0-454 | 0-295 | |
| | **Minority-Language SVI Subscore** | | | | | | |
| | Mean (SD) | 0.30 (0.11) | 0.49 (0.03) | 0.61 (0.05) | 0.77 (0.03) | 0.84 (0.02) | |
| | Range | 0.01-0.42 | 0.42-0.54 | 0.54-0.72 | 0.72-0.81 | 0.81-0.95 | |
| | **% Alive/Lost on Last Follow-up** | 0.628 | 0.635 | 0.603 | 0.697 | 0.657 | |
| | **% Dead on Last Follow-up** | 0.372 | 0.365 | 0.397 | 0.303 | 0.343 | |

© 2023 Fei-Zhang DJ et al. *JAMA Network Open.*

| | | Relative Minority-Language-SVI %-Tile | | | | | |
|---|---|---|---|---|---|---|---|
| **ICCC Class[1]** | | **<20** | **20-39.99** | **40-59.99** | **60-79.99** | **80-99.99** | **p-value** |
| Intracranial/spinal Germ Cell Tumors | **Sample Size** | 207 | 207 | 207 | 206 | 206 | |
| | **Months Surveyed** | | | | | | 0.009 |
| | Mean (SD) | 133 (117) | 128 (113) | 104 (95) | 115 (100) | 110 (79) | |
| | Median (IQR) | 95 (32-190) | 104 (32-189) | 82 (23-157) | 97 (30-172) | 94 (43-165) | |
| | Range | 0-443 | 0-461 | 0-463 | 0-458 | 0-291 | |
| | **Minority-Language SVI Subscore** | | | | | | |
| | Mean (SD) | 0.33 (0.10) | 0.52 (0.05) | 0.70 (0.05) | 0.79 (0.01) | 0.84 (0.01) | |
| | Range | 0.00-0.45 | 0.45-0.61 | 0.61-0.76 | 0.76-0.81 | 0.81-0.88 | |
| | **% Alive/Lost on Last Follow-up** | 0.787 | 0.773 | 0.768 | 0.777 | 0.816 | |
| | **% Dead on Last Follow-up** | 0.213 | 0.227 | 0.232 | 0.223 | 0.184 | |
| Hodgkin Lymphomas | **Sample Size** | 343 | 343 | 343 | 343 | 342 | |
| | **Months Surveyed** | | | | | | <0.001 |
| | Mean (SD) | 194 (140) | 200 (140) | 213 (141) | 170 (132) | 145 (107) | |
| | Median (IQR) | 165 (84-290) | 165 (84-309) | 195 (86-332) | 137 (68-246) | 128 (60-206) | |
| | Range | 0-497 | 0-497 | 0-494 | 0-501 | 0-496 | |
| | **Minority-Language SVI Subscore** | | | | | | |
| | Mean (SD) | 0.30 (0.10) | 0.47 (0.03) | 0.58 (0.03) | 0.73 (0.04) | 0.83 (0.02) | |
| | Range | 0.00-0.42 | 0.42-0.54 | 0.54-0.64 | 0.64-0.79 | 0.79-0.95 | |

© 2023 Fei-Zhang DJ et al. *JAMA Network Open.*

| | | Relative Minority-Language-SVI %-Tile | | | | | |
|---|---|---|---|---|---|---|---|
| **ICCC Class[1]** | | **<20** | **20-39.99** | **40-59.99** | **60-79.99** | **80-99.99** | **p-value** |
| | **% Alive/Lost on Last Follow-up** | 0.91 | 0.83 | 0.86 | 0.88 | 0.94 | |
| | **% Dead on Last Follow-up** | 0.09 | 0.17 | 0.14 | 0.12 | 0.06 | |
| Non-Hodgkin Lymphomas | **Sample Size** | 286 | 286 | 285 | 285 | 285 | |
| | **Months Surveyed** | | | | | | <0.001 |
| | Mean (SD) | 134 (125) | 127 (115) | 126 (118) | 122 (112) | 98 (79) | |
| | Median (IQR) | 104 (32-184) | 99 (36-179) | 88 (35-178) | 99 (33-170) | 78 (32-148) | |
| | Range | 0-491 | 0-494 | 0-489 | 0-498 | 0-394 | |
| | **Minority-Language SVI Subscore** | | | | | | |
| | Mean (SD) | 0.32 (0.10) | 0.50 (0.03) | 0.64 (0.05) | 0.76 (0.03) | 0.83 (0.02) | |
| | Range | 0.00-0.43 | 0.43-0.56 | 0.56-0.72 | 0.72-0.80 | 0.80-0.95 | |
| | **% Alive/Lost on Last Follow-up** | 0.874 | 0.850 | 0.832 | 0.895 | 0.856 | |
| | **% Dead on Last Follow-up** | 0.126 | 0.150 | 0.168 | 0.105 | 0.144 | |
| Malignant Melanomas | **Sample Size** | 563 | 563 | 562 | 562 | 562 | |
| | **Months Surveyed** | | | | | | <0.001 |
| | Mean (SD) | 158 (126) | 163 (123) | 174 (127) | 142 (109) | 140 (105) | |
| | Median (IQR) | 131 (55-218) | 143 (64-204) | 156 (66-260) | 128 (60-184) | 138 (53-195) | |
| | Range | 0-502 | 0-498 | 0-502 | 0-489 | 0-500 | |
| | **Minority-Language SVI Subscore** | | | | | | |

© 2023 Fei-Zhang DJ et al. *JAMA Network Open.*

| | | Relative Minority-Language-SVI %-Tile | | | | | |
|---|---|---|---|---|---|---|---|
| ICCC Class[1] | | **<20** | **20-39.99** | **40-59.99** | **60-79.99** | **80-99.99** | **p-value** |
| | Mean (SD) | 0.28 (0.10) | 0.45 (0.04) | 0.58 (0.05) | 0.73 (0.03) | 0.82 (0.03) | |
| | Range | 0.00-0.40 | 0.40-0.50 | 0.50-0.67 | 0.67-0.77 | 0.77-0.95 | |
| | **% Alive/Lost on Last Follow-up** | 0.909 | 0.909 | 0.902 | 0.920 | 0.899 | |
| | **% Dead on Last Follow-up** | 0.091 | 0.091 | 0.098 | 0.080 | 0.101 | |
| Other-NOS Gliomas | **Sample Size** | 592 | 591 | 591 | 591 | 591 | |
| | **Months Surveyed** | | | | | | <0.001 |
| | Mean (SD) | 104 (118) | 103 (118) | 109 (123) | 72 (98) | 75 (93) | |
| | Median (IQR) | 59 (11-162) | 46 (11-163) | 58 (12-174) | 24 (9-107) | 22 (8-124) | |
| | Range | 0-498 | 0-503 | 0-503 | 0-485 | 0-498 | |
| | **Minority-Language SVI Subscore** | | | | | | |
| | Mean (SD) | 0.28 (0.10) | 0.47 (0.03) | 0.58 (0.03) | 0.73 (0.04) | 0.83 (0.02) | |
| | Range | 0.02-0.41 | 0.41-0.51 | 0.51-0.62 | 0.62-0.79 | 0.79-0.95 | |
| | **% Alive/Lost on Last Follow-up** | 0.62 | 0.54 | 0.57 | 0.54 | 0.50 | |
| | **% Dead on Last Follow-up** | 0.38 | 0.46 | 0.43 | 0.46 | 0.50 | |
| Other or Unspecified Soft Tissue Sarcomas | **Sample Size** | 281 | 281 | 281 | 280 | 280 | |
| | **Months Surveyed** | | | | | | <0.001 |
| | Mean (SD) | 158 (128) | 159 (138) | 157 (133) | 148 (139) | 118 (96) | |
| | Median (IQR) | 140 (52-234) | 126 (46-223) | 118 (46-233) | 109 (33-211) | 96 (33-188) | |

© 2023 Fei-Zhang DJ et al. *JAMA Network Open.*

| | | Relative Minority-Language-SVI %-Tile | | | | | |
|---|---|---|---|---|---|---|---|
| ICCC Class[1] | | <20 | 20-39.99 | 40-59.99 | 60-79.99 | 80-99.99 | p-value |
| | Range | 0-499 | 0-499 | 0-485 | 0-501 | 0-502 | |
| | **Minority-Language SVI Subscore** | | | | | | |
| | Mean (SD) | 0.31 (0.10) | 0.50 (0.03) | 0.63 (0.05) | 0.77 (0.03) | 0.83 (0.02) | |
| | Range | 0.04-0.43 | 0.43-0.56 | 0.56-0.72 | 0.72-0.80 | 0.80-0.95 | |
| | **% Alive/Lost on Last Follow-up** | 0.847 | 0.858 | 0.833 | 0.850 | 0.861 | |
| | **% Dead on Last Follow-up** | 0.153 | 0.142 | 0.167 | 0.150 | 0.139 | |
| Retinoblastomas | **Sample Size** | 396 | 395 | 395 | 395 | 395 | |
| | **Months Surveyed** | | | | | | <0.001 |
| | Mean (SD) | 166 (134) | 172 (134) | 156 (128) | 139 (127) | 129 (84) | |
| | Median (IQR) | 130 (58-254) | 150 (52-261) | 130 (43-244) | 102 (40-196) | 132 (52-196) | |
| | Range | 0-502 | 0-496 | 0-501 | 0-498 | 0-373 | |
| | **Minority-Language SVI Subscore** | | | | | | |
| | Mean (SD) | 0.31 (0.11) | 0.49 (0.03) | 0.64 (0.05) | 0.77 (0.03) | 0.84 (0.02) | |
| | Range | 0.03-0.42 | 0.42-0.56 | 0.56-0.72 | 0.72-0.81 | 0.81-0.95 | |
| | **% Alive/Lost on Last Follow-up** | 0.944 | 0.947 | 0.929 | 0.932 | 0.952 | |
| | **% Dead on Last Follow-up** | 0.056 | 0.053 | 0.071 | 0.068 | 0.048 | |
| Rhabdomyosarcomas | **Sample Size** | 225 | 225 | 225 | 225 | 224 | |
| | **Months Surveyed** | | | | | | 0.003 |

© 2023 Fei-Zhang DJ et al. *JAMA Network Open.*

| | | Relative Minority-Language-SVI %-Tile | | | | | |
|---|---|---|---|---|---|---|---|
| **ICCC Class[1]** | | **<20** | **20-39.99** | **40-59.99** | **60-79.99** | **80-99.99** | **p-value** |
| | Mean (SD) | 119 (124) | 126 (131) | 139 (137) | 99 (107) | 96 (92) | |
| | Median (IQR) | 66 (20-183) | 66 (20-186) | 84 (28-215) | 62 (24-135) | 62 (16-163) | |
| | Range | 0-500 | 1-479 | 0-503 | 1-489 | 0-453 | |
| | **Minority-Language SVI Subscore** | | | | | | |
| | Mean (SD) | 0.30 (0.10) | 0.48 (0.03) | 0.61 (0.05) | 0.76 (0.03) | 0.83 (0.02) | |
| | Range | 0.00-0.42 | 0.42-0.54 | 0.54-0.72 | 0.72-0.80 | 0.80-0.95 | |
| | **% Alive/Lost on Last Follow-up** | 0.62 | 0.65 | 0.72 | 0.62 | 0.69 | |
| | **% Dead on Last Follow-up** | 0.38 | 0.35 | 0.28 | 0.38 | 0.31 | |
| Thyroid Carcinomas | **Sample Size** | 861 | 861 | 860 | 860 | 860 | |
| | **Months Surveyed** | | | | | | <0.001 |
| | Mean (SD) | 147 (129) | 156 (135) | 153 (133) | 124 (113) | 117 (91) | |
| | Median (IQR) | 107 (49-197) | 114 (48-226) | 113 (45-215) | 93 (42-174) | 97 (43-176) | |
| | Range | 0-503 | 0-501 | 0-502 | 0-500 | 0-451 | |
| | **Minority-Language SVI Subscore** | | | | | | |
| | Mean (SD) | 0.30 (0.11) | 0.47 (0.04) | 0.62 (0.05) | 0.76 (0.03) | 0.83 (0.02) | |
| | Range | 0.00-0.42 | 0.42-0.54 | 0.54-0.72 | 0.72-0.80 | 0.80-0.95 | |
| | **% Alive/Lost on Last Follow-up** | 0.986 | 0.966 | 0.974 | 0.970 | 0.971 | |
| | **% Dead on Last Follow-up** | 0.014 | 0.034 | 0.026 | 0.030 | 0.029 | |

[1]By International Classification of Childhood Cancer

© 2023 Fei-Zhang DJ et al. *JAMA Network Open.*

| | | Relative Minority-Language-SVI %-Tile | | | | | |
|---|---|---|---|---|---|---|---|
| ICCC Class[1] | | <20 | 20-39.99 | 40-59.99 | 60-79.99 | 80-99.99 | p-value |
| | | Relative Household Composition-SVI %-Tile | | | | | |
| ICCC Class[1] | | <20 | 20-39.99 | 40-59.99 | 60-79.99 | 80-99.99 | p-value |
| Astrocytomas | **Sample Size** | 1,744 | 1,743 | 1,743 | 1,743 | 1,743 | |
| | **Months Surveyed** | | | | | | <0.001 |
| | Mean (SD) | 131 (120) | 125 (114) | 131 (121) | 116 (111) | 117 (115) | |
| | Median (IQR) | 96 (29-193) | 101 (26-183) | 98 (24-214) | 86 (25-169) | 82 (21-176) | |
| | Range | 0-503 | 0-503 | 0-500 | 0-503 | 0-501 | |
| | **Household Composition SVI Subscore** | | | | | | |
| | Mean (SD) | 0.25 (0.04) | 0.35 (0.01) | 0.40 (0.03) | 0.51 (0.04) | 0.69 (0.07) | |
| | Range | 0.15-0.33 | 0.33-0.36 | 0.36-0.46 | 0.46-0.60 | 0.60-0.96 | |
| | **% Alive/Lost on Last Follow-up** | 0.770 | 0.750 | 0.779 | 0.778 | 0.732 | |
| | **% Dead on Last Follow-up** | 0.230 | 0.250 | 0.221 | 0.222 | 0.268 | |
| Intracranial/spinal Embryonal Tumors | **Sample Size** | 690 | 690 | 690 | 690 | 690 | |
| | **Months Surveyed** | | | | | | 0.026 |
| | Mean (SD) | 98 (107) | 97 (108) | 94 (106) | 88 (97) | 89 (104) | |
| | Median (IQR) | 59 (13-156) | 59 (13-152) | 49 (12-151) | 52 (13-132) | 45 (12-138) | |
| | Range | 0-483 | 0-490 | 0-474 | 0-492 | 0-487 | |
| | **Household Composition SVI Subscore** | | | | | | |
| | Mean (SD) | 0.26 (0.04) | 0.35 (0.01) | 0.40 (0.03) | 0.50 (0.03) | 0.68 (0.08) | |
| | Range | 0.15-0.33 | 0.33-0.36 | 0.36-0.46 | 0.46-0.57 | 0.57-0.94 | |

© 2023 Fei-Zhang DJ et al. *JAMA Network Open.*

| | | Relative Minority-Language-SVI %-Tile | | | | | |
|---|---|---|---|---|---|---|---|
| **ICCC Class[1]** | | **<20** | **20-39.99** | **40-59.99** | **60-79.99** | **80-99.99** | **p-value** |
| | **% Alive/Lost on Last Follow-up** | 0.558 | 0.520 | 0.562 | 0.587 | 0.559 | |
| | **% Dead on Last Follow-up** | 0.442 | 0.480 | 0.438 | 0.413 | 0.441 | |
| Ependymomas & Choroid Plexus Tumors | **Sample Size** | 301 | 301 | 300 | 300 | 300 | |
| | **Months Surveyed** | | | | | | 0.297 |
| | Mean (SD) | 106 (107) | 91 (91) | 100 (103) | 92 (93) | 96 (95) | |
| | Median (IQR) | 68 (25-159) | 61 (20-138) | 60 (21-163) | 59 (20-142) | 63 (25-139) | |
| | Range | 0-493 | 0-482 | 0-486 | 0-495 | 0-486 | |
| | **Household Composition SVI Subscore** | | | | | | |
| | Mean (SD) | 0.26 (0.04) | 0.35 (0.01) | 0.40 (0.03) | 0.51 (0.03) | 0.68 (0.07) | |
| | Range | 0.15-0.33 | 0.33-0.36 | 0.36-0.46 | 0.46-0.59 | 0.59-0.96 | |
| | **% Alive/Lost on Last Follow-up** | 0.64 | 0.59 | 0.69 | 0.66 | 0.63 | |
| | **% Dead on Last Follow-up** | 0.36 | 0.41 | 0.31 | 0.34 | 0.37 | |
| Intracranial/spinal Germ Cell Tumors | **Sample Size** | 207 | 207 | 207 | 206 | 206 | |
| | **Months Surveyed** | | | | | | 0.097 |
| | Mean (SD) | 122 (108) | 130 (100) | 116 (101) | 103 (103) | 117 (98) | |
| | Median (IQR) | 100 (30-183) | 122 (53-184) | 89 (34-188) | 76 (26-156) | 95 (33-164) | |
| | Range | 0-463 | 0-443 | 0-452 | 0-461 | 0-443 | |

© 2023 Fei-Zhang DJ et al. *JAMA Network Open.*

| | | Relative Minority-Language-SVI %-Tile | | | | | |
|---|---|---|---|---|---|---|---|
| **ICCC Class[1]** | | **<20** | **20-39.99** | **40-59.99** | **60-79.99** | **80-99.99** | **p-value** |
| | **Household Composition SVI Subscore** | | | | | | |
| | Mean (SD) | 0.24 (0.04) | 0.34 (0.01) | 0.38 (0.02) | 0.48 (0.03) | 0.66 (0.09) | |
| | Range | 0.15-0.27 | 0.29-0.36 | 0.36-0.42 | 0.42-0.53 | 0.53-0.89 | |
| | **% Alive/Lost on Last Follow-up** | 0.758 | 0.749 | 0.826 | 0.820 | 0.767 | |
| | **% Dead on Last Follow-up** | 0.242 | 0.251 | 0.174 | 0.180 | 0.233 | |
| Hodgkin Lymphomas | **Sample Size** | 343 | 343 | 343 | 343 | 342 | |
| | **Months Surveyed** | | | | | | 0.004 |
| | Mean (SD) | 196 (141) | 179 (130) | 213 (135) | 161 (126) | 172 (134) | |
| | Median (IQR) | 169 (72-318) | 155 (77-262) | 203 (92-314) | 131 (70-201) | 140 (68-234) | |
| | Range | 0-490 | 0-496 | 0-501 | 0-496 | 0-497 | |
| | **Household Composition SVI Subscore** | | | | | | |
| | Mean (SD) | 0.27 (0.05) | 0.35 (0.01) | 0.41 (0.03) | 0.51 (0.04) | 0.69 (0.08) | |
| | Range | 0.15-0.34 | 0.34-0.36 | 0.36-0.47 | 0.47-0.59 | 0.59-0.91 | |
| | **% Alive/Lost on Last Follow-up** | 0.878 | 0.907 | 0.854 | 0.898 | 0.877 | |
| | **% Dead on Last Follow-up** | 0.122 | 0.093 | 0.146 | 0.102 | 0.123 | |
| Non-Hodgkin Lymphomas | **Sample Size** | 286 | 286 | 285 | 285 | 285 | |
| | **Months Surveyed** | | | | | | 0.107 |
| | Mean (SD) | 134 (126) | 120 (104) | 118 (108) | 115 (106) | 119 (112) | |

© 2023 Fei-Zhang DJ et al. *JAMA Network Open.*

| | | Relative Minority-Language-SVI %-Tile | | | | | |
|---|---|---|---|---|---|---|---|
| ICCC Class[1] | | <20 | 20-39.99 | 40-59.99 | 60-79.99 | 80-99.99 | p-value |
| | Median (IQR) | 96 (34-194) | 96 (38-170) | 86 (33-192) | 87 (32-160) | 103 (34-170) | |
| | Range | 0-498 | 0-479 | 0-477 | 0-489 | 0-494 | |
| | **Household Composition SVI Subscore** | | | | | | |
| | Mean (SD) | 0.25 (0.04) | 0.35 (0.01) | 0.39 (0.03) | 0.50 (0.03) | 0.68 (0.08) | |
| | Range | 0.09-0.33 | 0.33-0.36 | 0.36-0.44 | 0.44-0.57 | 0.57-0.96 | |
| | **% Alive/Lost on Last Follow-up** | 0.853 | 0.867 | 0.860 | 0.870 | 0.856 | |
| | **% Dead on Last Follow-up** | 0.147 | 0.133 | 0.140 | 0.130 | 0.144 | |
| Malignant Melanomas | **Sample Size** | 563 | 563 | 562 | 562 | 562 | |
| | **Months Surveyed** | | | | | | <0.001 |
| | Mean (SD) | 164 (122) | 170 (124) | 147 (109) | 156 (120) | 140 (117) | |
| | Median (IQR) | 147 (68-212) | 148 (70-248) | 138 (61-201) | 138 (60-198) | 123 (47-187) | |
| | Range | 0-500 | 0-498 | 0-490 | 0-502 | 0-497 | |
| | **Household Composition SVI Subscore** | | | | | | |
| | Mean (SD) | 0.24 (0.03) | 0.34 (0.01) | 0.39 (0.02) | 0.49 (0.03) | 0.67 (0.08) | |
| | Range | 0.15-0.29 | 0.29-0.36 | 0.36-0.44 | 0.44-0.54 | 0.54-0.91 | |
| | **% Alive/Lost on Last Follow-up** | 0.929 | 0.885 | 0.904 | 0.900 | 0.922 | |
| | **% Dead on Last Follow-up** | 0.071 | 0.115 | 0.096 | 0.100 | 0.078 | |
| Other-NOS Gliomas | **Sample Size** | 592 | 591 | 591 | 591 | 591 | |

© 2023 Fei-Zhang DJ et al. *JAMA Network Open.*

| | | Relative Minority-Language-SVI %-Tile | | | | | |
|---|---|---|---|---|---|---|---|
| ICCC Class[1] | | <20 | 20-39.99 | 40-59.99 | 60-79.99 | 80-99.99 | p-value |
| | **Months Surveyed** | | | | | | 0.009 |
| | Mean (SD) | 103 (123) | 98 (108) | 86 (110) | 85 (104) | 91 (112) | |
| | Median (IQR) | 40 (10-166) | 62 (11-150) | 26 (9-130) | 35 (11-134) | 42 (10-143) | |
| | Range | 0-503 | 0-503 | 0-496 | 0-498 | 0-501 | |
| | **Household Composition SVI Subscore** | | | | | | |
| | Mean (SD) | 0.25 (0.04) | 0.35 (0.01) | 0.40 (0.03) | 0.51 (0.03) | 0.69 (0.07) | |
| | Range | 0.15-0.33 | 0.33-0.36 | 0.36-0.46 | 0.46-0.59 | 0.59-0.95 | |
| | **% Alive/Lost on Last Follow-up** | 0.557 | 0.574 | 0.521 | 0.558 | 0.550 | |
| | **% Dead on Last Follow-up** | 0.443 | 0.426 | 0.479 | 0.442 | 0.450 | |
| Other or Unspecified Soft Tissue Sarcomas | **Sample Size** | 281 | 281 | 281 | 280 | 280 | |
| | **Months Surveyed** | | | | | | 0.009 |
| | Mean (SD) | 158 (133) | 159 (130) | 151 (133) | 130 (113) | 141 (131) | |
| | Median (IQR) | 130 (47-236) | 122 (57-226) | 115 (36-236) | 100 (34-186) | 106 (36-195) | |
| | Range | 0-486 | 0-498 | 0-501 | 0-478 | 0-502 | |
| | **Household Composition SVI Subscore** | | | | | | |
| | Mean (SD) | 0.26 (0.04) | 0.35 (0.01) | 0.40 (0.03) | 0.51 (0.03) | 0.67 (0.07) | |
| | Range | 0.15-0.33 | 0.33-0.36 | 0.36-0.46 | 0.46-0.58 | 0.58-0.91 | |
| | **% Alive/Lost on Last Follow-up** | 0.865 | 0.865 | 0.843 | 0.821 | 0.854 | |

© 2023 Fei-Zhang DJ et al. *JAMA Network Open.*

| | | Relative Minority-Language-SVI %-Tile | | | | | |
|---|---|---|---|---|---|---|---|
| **ICCC Class[1]** | | **<20** | **20-39.99** | **40-59.99** | **60-79.99** | **80-99.99** | **p-value** |
| | **% Dead on Last Follow-up** | 0.135 | 0.135 | 0.157 | 0.179 | 0.146 | |
| Retinoblastomas | **Sample Size** | 396 | 395 | 395 | 395 | 395 | |
| | **Months Surveyed** | | | | | | <0.001 |
| | Mean (SD) | 171 (128) | 156 (112) | 160 (127) | 129 (118) | 147 (131) | |
| | Median (IQR) | 149 (63-258) | 140 (70-196) | 141 (43-250) | 94 (38-183) | 116 (42-199) | |
| | Range | 0-498 | 0-486 | 0-501 | 0-502 | 0-496 | |
| | **Household Composition SVI Subscore** | | | | | | |
| | Mean (SD) | 0.26 (0.05) | 0.35 (0.01) | 0.40 (0.03) | 0.51 (0.03) | 0.69 (0.07) | |
| | Range | 0.15-0.34 | 0.34-0.36 | 0.36-0.46 | 0.46-0.59 | 0.59-0.91 | |
| | **% Alive/Lost on Last Follow-up** | 0.934 | 0.952 | 0.929 | 0.954 | 0.934 | |
| | **% Dead on Last Follow-up** | 0.066 | 0.048 | 0.071 | 0.046 | 0.066 | |
| Rhabdomyosarcomas | **Sample Size** | 225 | 225 | 225 | 225 | 224 | |
| | **Months Surveyed** | | | | | | 0.666 |
| | Mean (SD) | 120 (119) | 102 (107) | 136 (136) | 111 (113) | 111 (123) | |
| | Median (IQR) | 76 (21-185) | 65 (18-159) | 81 (27-222) | 78 (22-166) | 54 (18-171) | |
| | Range | 0-489 | 0-474 | 0-503 | 1-502 | 0-494 | |
| | **Household Composition SVI Subscore** | | | | | | |
| | Mean (SD) | 0.27 (0.05) | 0.35 (0.01) | 0.41 (0.03) | 0.51 (0.03) | 0.68 (0.08) | |
| | Range | 0.15-0.34 | 0.34-0.36 | 0.36-0.47 | 0.47-0.59 | 0.59-0.97 | |

© 2023 Fei-Zhang DJ et al. *JAMA Network Open.*

| | | Relative Minority-Language-SVI %-Tile | | | | | |
|---|---|---|---|---|---|---|---|
| **ICCC Class[1]** | | **<20** | **20-39.99** | **40-59.99** | **60-79.99** | **80-99.99** | **p-value** |
| | **% Alive/Lost on Last Follow-up** | 0.693 | 0.662 | 0.658 | 0.676 | 0.603 | |
| | **% Dead on Last Follow-up** | 0.307 | 0.338 | 0.342 | 0.324 | 0.397 | |
| Thyroid Carcinomas | **Sample Size** | 861 | 861 | 860 | 860 | 860 | |
| | **Months Surveyed** | | | | | | 0.019 |
| | Mean (SD) | 138 (117) | 157 (122) | 134 (127) | 135 (125) | 133 (120) | |
| | Median (IQR) | 107 (46-189) | 122 (71-204) | 90 (34-201) | 91 (43-185) | 100 (41-183) | |
| | Range | 0-502 | 0-501 | 0-503 | 0-500 | 0-490 | |
| | **Household Composition SVI Subscore** | | | | | | |
| | Mean (SD) | 0.25 (0.04) | 0.35 (0.01) | 0.39 (0.03) | 0.50 (0.03) | 0.68 (0.08) | |
| | Range | 0.15-0.32 | 0.32-0.36 | 0.36-0.45 | 0.45-0.57 | 0.57-0.96 | |
| | **% Alive/Lost on Last Follow-up** | 0.9768 | 0.9675 | 0.9698 | 0.9767 | 0.9767 | |
| | **% Dead on Last Follow-up** | 0.0232 | 0.0325 | 0.0302 | 0.0233 | 0.0233 | |
| [1]By International Classification of Childhood Cancer | | | | | | | |

| | | Relative Housing-Transportation-SVI %-Tile | | | | | |
|---|---|---|---|---|---|---|---|
| **ICCC Class[1]** | | **<20** | **20-39.99** | **40-59.99** | **60-79.99** | **80-99.99** | **p-value** |
| Astrocytomas | **Sample Size** | 1,744 | 1,743 | 1,743 | 1,743 | 1,743 | |
| | **Months Surveyed** | | | | | | <0.001 |
| | Mean (SD) | 139 (126) | 141 (131) | 118 (114) | 115 (110) | 107 (96) | |
| | Median (IQR) | 104 (29-201) | 100 (26-215) | 87 (22-178) | 84 (26-172) | 86 (22-167) | |

© 2023 Fei-Zhang DJ et al. *JAMA Network Open.*

| | | Relative Housing-Transportation-SVI %-Tile | | | | | |
|---|---|---|---|---|---|---|---|
| **ICCC Class[1]** | | **<20** | **20-39.99** | **40-59.99** | **60-79.99** | **80-99.99** | **p-value** |
| | Range | 0-503 | 0-503 | 0-502 | 0-503 | 0-503 | |
| | **Housing-Transportation SVI Subscore** | | | | | | |
| | Mean (SD) | 0.34 (0.05) | 0.44 (0.02) | 0.52 (0.02) | 0.58 (0.02) | 0.65 (0.05) | |
| | Range | 0.05-0.40 | 0.40-0.48 | 0.48-0.55 | 0.55-0.60 | 0.61-0.89 | |
| | **% Alive/Lost on Last Follow-up** | 0.763 | 0.755 | 0.758 | 0.776 | 0.757 | |
| | **% Dead on Last Follow-up** | 0.237 | 0.245 | 0.242 | 0.224 | 0.243 | |
| Intracranial/spinal Embryonal Tumors | **Sample Size** | 690 | 690 | 690 | 690 | 690 | |
| | **Months Surveyed** | | | | | | 0.003 |
| | Mean (SD) | 99 (110) | 99 (116) | 93 (107) | 90 (93) | 84 (93) | |
| | Median (IQR) | 55 (14-152) | 48 (11-160) | 50 (13-142) | 58 (14-143) | 49 (12-136) | |
| | Range | 0-484 | 0-488 | 0-492 | 0-483 | 0-463 | |
| | **Housing-Transportation SVI Subscore** | | | | | | |
| | Mean (SD) | 0.35 (0.05) | 0.46 (0.03) | 0.54 (0.02) | 0.59 (0.02) | 0.65 (0.05) | |
| | Range | 0.18-0.42 | 0.42-0.50 | 0.50-0.55 | 0.55-0.62 | 0.62-0.89 | |
| | **% Alive/Lost on Last Follow-up** | 0.554 | 0.525 | 0.565 | 0.577 | 0.567 | |
| | **% Dead on Last Follow-up** | 0.446 | 0.475 | 0.435 | 0.423 | 0.433 | |
| Ependymomas & Choroid Plexus Tumors | **Sample Size** | 301 | 301 | 300 | 300 | 300 | |

© 2023 Fei-Zhang DJ et al. *JAMA Network Open.*

| | | Relative Housing-Transportation-SVI %-Tile | | | | | |
|---|---|---|---|---|---|---|---|
| **ICCC Class[1]** | | **<20** | **20-39.99** | **40-59.99** | **60-79.99** | **80-99.99** | **p-value** |
| | **Months Surveyed** | | | | | | 0.013 |
| | Mean (SD) | 99 (103) | 107 (113) | 98 (98) | 100 (96) | 80 (75) | |
| | Median (IQR) | 61 (22-154) | 61 (21-162) | 62 (21-156) | 68 (29-148) | 60 (18-122) | |
| | Range | 0-478 | 0-495 | 0-445 | 0-493 | 0-407 | |
| | **Housing-Transportation SVI Subscore** | | | | | | |
| | Mean (SD) | 0.35 (0.04) | 0.45 (0.02) | 0.53 (0.02) | 0.58 (0.02) | 0.65 (0.05) | |
| | Range | 0.05-0.41 | 0.41-0.49 | 0.49-0.55 | 0.55-0.62 | 0.62-0.88 | |
| | **% Alive/Lost on Last Follow-up** | 0.641 | 0.608 | 0.647 | 0.657 | 0.667 | |
| | **% Dead on Last Follow-up** | 0.359 | 0.392 | 0.353 | 0.343 | 0.333 | |
| Intracranial/spinal Germ Cell Tumors | **Sample Size** | 207 | 207 | 207 | 206 | 206 | |
| | **Months Surveyed** | | | | | | 0.105 |
| | Mean (SD) | 126 (111) | 123 (115) | 114 (96) | 116 (94) | 111 (93) | |
| | Median (IQR) | 96 (40-186) | 86 (26-190) | 92 (35-176) | 102 (38-163) | 88 (34-161) | |
| | Range | 0-443 | 0-461 | 0-463 | 0-458 | 0-446 | |
| | **Housing-Transportation SVI Subscore** | | | | | | |
| | Mean (SD) | 0.35 (0.05) | 0.47 (0.03) | 0.55 (0.01) | 0.59 (0.02) | 0.65 (0.05) | |
| | Range | 0.18-0.43 | 0.43-0.52 | 0.52-0.55 | 0.55-0.62 | 0.62-0.89 | |
| | **% Alive/Lost on Last Follow-up** | 0.778 | 0.773 | 0.754 | 0.830 | 0.786 | |

© 2023 Fei-Zhang DJ et al. *JAMA Network Open.*

| | | Relative Housing-Transportation-SVI %-Tile | | | | | |
|---|---|---|---|---|---|---|---|
| ICCC Class[1] | | <20 | 20-39.99 | 40-59.99 | 60-79.99 | 80-99.99 | p-value |
| | **% Dead on Last Follow-up** | 0.222 | 0.227 | 0.246 | 0.170 | 0.214 | |
| Hodgkin Lymphomas | **Sample Size** | 343 | 343 | 343 | 343 | 342 | |
| | **Months Surveyed** | | | | | | <0.001 |
| | Mean (SD) | 197 (143) | 216 (139) | 195 (141) | 167 (132) | 146 (104) | |
| | Median (IQR) | 161 (79-316) | 195 (97-328) | 157 (84-298) | 133 (64-246) | 130 (63-202) | |
| | Range | 0-496 | 1-497 | 0-501 | 0-498 | 0-495 | |
| | **Housing-Transportation SVI Subscore** | | | | | | |
| | Mean (SD) | 0.34 (0.05) | 0.43 (0.02) | 0.50 (0.03) | 0.57 (0.02) | 0.65 (0.05) | |
| | Range | 0.05-0.40 | 0.40-0.46 | 0.46-0.54 | 0.54-0.60 | 0.60-0.86 | |
| | **% Alive/Lost on Last Follow-up** | 0.866 | 0.843 | 0.869 | 0.910 | 0.927 | |
| | **% Dead on Last Follow-up** | 0.134 | 0.157 | 0.131 | 0.090 | 0.073 | |
| Non-Hodgkin Lymphomas | **Sample Size** | 286 | 286 | 285 | 285 | 285 | |
| | **Months Surveyed** | | | | | | 0.007 |
| | Mean (SD) | 129 (116) | 131 (122) | 122 (115) | 115 (107) | 109 (94) | |
| | Median (IQR) | 96 (34-185) | 98 (34-188) | 87 (32-164) | 80 (34-173) | 95 (34-160) | |
| | Range | 0-491 | 0-494 | 0-479 | 0-498 | 0-473 | |
| | **Housing-Transportation SVI Subscore** | | | | | | |
| | Mean (SD) | 0.34 (0.05) | 0.45 (0.02) | 0.52 (0.02) | 0.58 (0.02) | 0.65 (0.06) | |

© 2023 Fei-Zhang DJ et al. *JAMA Network Open.*

| | | Relative Housing-Transportation-SVI %-Tile | | | | | |
|---|---|---|---|---|---|---|---|
| **ICCC Class[1]** | | **<20** | **20-39.99** | **40-59.99** | **60-79.99** | **80-99.99** | **p-value** |
| | Range | 0.05-0.40 | 0.40-0.49 | 0.49-0.55 | 0.55-0.62 | 0.62-0.89 | |
| | **% Alive/Lost on Last Follow-up** | 0.864 | 0.853 | 0.842 | 0.884 | 0.863 | |
| | **% Dead on Last Follow-up** | 0.136 | 0.147 | 0.158 | 0.116 | 0.137 | |
| Malignant Melanomas | **Sample Size** | 563 | 563 | 562 | 562 | 562 | |
| | **Months Surveyed** | | | | | | <0.001 |
| | Mean (SD) | 162 (122) | 165 (132) | 156 (122) | 154 (119) | 141 (96) | |
| | Median (IQR) | 140 (59-228) | 136 (58-242) | 142 (59-199) | 138 (66-195) | 142 (65-190) | |
| | Range | 0-489 | 0-502 | 0-498 | 0-500 | 0-477 | |
| | **Housing-Transportation SVI Subscore** | | | | | | |
| | Mean (SD) | 0.33 (0.05) | 0.43 (0.03) | 0.52 (0.02) | 0.57 (0.02) | 0.65 (0.05) | |
| | Range | 0.05-0.38 | 0.38-0.47 | 0.47-0.55 | 0.55-0.60 | 0.60-0.87 | |
| | **% Alive/Lost on Last Follow-up** | 0.890 | 0.895 | 0.911 | 0.922 | 0.922 | |
| | **% Dead on Last Follow-up** | 0.110 | 0.105 | 0.089 | 0.078 | 0.078 | |
| Other-NOS Gliomas | **Sample Size** | 592 | 591 | 591 | 591 | 591 | |
| | **Months Surveyed** | | | | | | <0.001 |
| | Mean (SD) | 110 (116) | 97 (122) | 86 (106) | 95 (119) | 75 (91) | |
| | Median (IQR) | 76 (13-168) | 32 (11-150) | 39 (10-132) | 37 (10-144) | 28 (8-125) | |
| | Range | 0-503 | 0-501 | 0-492 | 0-503 | 0-498 | |

© 2023 Fei-Zhang DJ et al. *JAMA Network Open.*

| | | Relative Housing-Transportation-SVI %-Tile | | | | | |
|---|---|---|---|---|---|---|---|
| **ICCC Class[1]** | | **<20** | **20-39.99** | **40-59.99** | **60-79.99** | **80-99.99** | **p-value** |
| | **Housing-Transportation SVI Subscore** | | | | | | |
| | Mean (SD) | 0.34 (0.04) | 0.45 (0.02) | 0.52 (0.02) | 0.58 (0.02) | 0.65 (0.05) | |
| | Range | 0.18-0.40 | 0.40-0.48 | 0.48-0.55 | 0.55-0.60 | 0.60-0.89 | |
| | **% Alive/Lost on Last Follow-up** | 0.63 | 0.50 | 0.53 | 0.55 | 0.55 | |
| | **% Dead on Last Follow-up** | 0.37 | 0.50 | 0.47 | 0.45 | 0.45 | |
| Other or Unspecified Soft Tissue Sarcomas | **Sample Size** | 281 | 281 | 281 | 280 | 280 | |
| | **Months Surveyed** | | | | | | <0.001 |
| | Mean (SD) | 152 (130) | 176 (144) | 151 (130) | 132 (122) | 129 (110) | |
| | Median (IQR) | 129 (44-222) | 148 (53-267) | 110 (45-208) | 98 (36-188) | 100 (34-198) | |
| | Range | 0-498 | 0-501 | 0-495 | 0-486 | 0-502 | |
| | **Housing-Transportation SVI Subscore** | | | | | | |
| | Mean (SD) | 0.35 (0.05) | 0.45 (0.02) | 0.53 (0.02) | 0.58 (0.02) | 0.64 (0.05) | |
| | Range | 0.18-0.41 | 0.41-0.49 | 0.49-0.55 | 0.55-0.62 | 0.62-0.88 | |
| | **% Alive/Lost on Last Follow-up** | 0.843 | 0.840 | 0.829 | 0.854 | 0.882 | |
| | **% Dead on Last Follow-up** | 0.157 | 0.160 | 0.171 | 0.146 | 0.118 | |
| Retinoblastomas | **Sample Size** | 396 | 395 | 395 | 395 | 395 | |

© 2023 Fei-Zhang DJ et al. *JAMA Network Open.*

| | | Relative Housing-Transportation-SVI %-Tile | | | | | |
|---|---|---|---|---|---|---|---|
| **ICCC Class[1]** | | **<20** | **20-39.99** | **40-59.99** | **60-79.99** | **80-99.99** | **p-value** |
| | **Months Surveyed** | | | | | | <0.001 |
| | Mean (SD) | 163 (127) | 181 (140) | 146 (122) | 149 (118) | 125 (102) | |
| | Median (IQR) | 135 (57-258) | 153 (55-284) | 115 (52-208) | 133 (52-195) | 103 (38-198) | |
| | Range | 0-502 | 0-501 | 0-497 | 0-498 | 0-482 | |
| | **Housing-Transportation SVI Subscore** | | | | | | |
| | Mean (SD) | 0.36 (0.05) | 0.46 (0.02) | 0.53 (0.02) | 0.59 (0.02) | 0.65 (0.05) | |
| | Range | 0.05-0.43 | 0.43-0.50 | 0.50-0.55 | 0.55-0.62 | 0.62-0.87 | |
| | **% Alive/Lost on Last Follow-up** | 0.944 | 0.942 | 0.942 | 0.949 | 0.927 | |
| | **% Dead on Last Follow-up** | 0.056 | 0.058 | 0.058 | 0.051 | 0.073 | |
| Rhabdomyosarcomas | **Sample Size** | 225 | 225 | 225 | 225 | 224 | |
| | **Months Surveyed** | | | | | | <0.001 |
| | Mean (SD) | 128 (121) | 149 (147) | 111 (118) | 87 (102) | 105 (99) | |
| | Median (IQR) | 92 (24-191) | 94 (26-256) | 69 (22-159) | 45 (17-123) | 64 (20-176) | |
| | Range | 0-500 | 0-503 | 0-494 | 1-489 | 0-454 | |
| | **Housing-Transportation SVI Subscore** | | | | | | |
| | Mean (SD) | 0.35 (0.04) | 0.45 (0.02) | 0.53 (0.02) | 0.58 (0.02) | 0.65 (0.05) | |
| | Range | 0.21-0.42 | 0.42-0.50 | 0.50-0.55 | 0.55-0.62 | 0.62-0.84 | |
| | **% Alive/Lost on Last Follow-up** | 0.70 | 0.67 | 0.67 | 0.59 | 0.67 | |

© 2023 Fei-Zhang DJ et al. *JAMA Network Open.*

| | | Relative Housing-Transportation-SVI %-Tile | | | | | |
|---|---|---|---|---|---|---|---|
| ICCC Class[1] | | <20 | 20-39.99 | 40-59.99 | 60-79.99 | 80-99.99 | p-value |
| | **% Dead on Last Follow-up** | 0.30 | 0.33 | 0.33 | 0.41 | 0.33 | |
| Thyroid Carcinomas | **Sample Size** | 861 | 861 | 860 | 860 | 860 | |
| | **Months Surveyed** | | | | | | <0.001 |
| | Mean (SD) | 152 (130) | 164 (141) | 135 (126) | 129 (110) | 117 (93) | |
| | Median (IQR) | 112 (52-213) | 120 (52-254) | 93 (40-190) | 101 (46-183) | 98 (40-171) | |
| | Range | 0-501 | 0-503 | 0-500 | 0-502 | 0-496 | |
| | **Housing-Transportation SVI Subscore** | | | | | | |
| | Mean (SD) | 0.34 (0.04) | 0.44 (0.02) | 0.52 (0.02) | 0.58 (0.02) | 0.65 (0.05) | |
| | Range | 0.05-0.40 | 0.40-0.48 | 0.48-0.55 | 0.55-0.62 | 0.62-0.87 | |
| | **% Alive/Lost on Last Follow-up** | 0.980 | 0.964 | 0.971 | 0.979 | 0.973 | |
| | **% Dead on Last Follow-up** | 0.020 | 0.036 | 0.029 | 0.021 | 0.027 | |

[1]By International Classification of Childhood Cancer

© 2023 Fei-Zhang DJ et al. *JAMA Network Open.*

**eTable 3.** ICCC Disease Class Trends in Survival Months by Relative SVI Percentile

| | | Relative Total SVI %-Tile | | | | | |
|---|---|---|---|---|---|---|---|
| **ICCC Class[1]** | | **<20** | **20-39.99** | **40-59.99** | **60-79.99** | **80-99.99** | **p-value** |
| Astrocytomas | **Sample Size** | 433 | 421 | 385 | 437 | 370 | |
| | **Months Survival** | | | | | | <0.001 |
| | Median (IQR) | 17 (7-60) | 17 (8-44) | 15 (8-45) | 13 (7-33) | 14 (7-26) | |
| | Range | 0-456 | 0-473 | 0-472 | 0-472 | 0-375 | |
| | Mean (SD) | 59 (94) | 55 (93) | 56 (95) | 46 (85) | 30 (51) | |
| | **Total SVI Score** | | | | | | |
| | Mean (SD) | 0.31 (0.06) | 0.41 (0.02) | 0.48 (0.02) | 0.58 (0.03) | 0.70 (0.07) | |
| | Range | 0.00-0.38 | 0.38-0.44 | 0.45-0.52 | 0.52-0.64 | 0.64-0.95 | |
| Intracranial/spinal Embryonal Tumors | **Sample Size** | 276 | 298 | 314 | 325 | 298 | |
| | **Months Survival** | | | | | | <0.001 |
| | Median (IQR) | 16 (6-49) | 16 (6-44) | 14 (6-38) | 13 (5-33) | 13 (5-31) | |
| | Range | 0-428 | 0-430 | 0-463 | 0-380 | 0-395 | |
| | Mean (SD) | 48 (79) | 40 (61) | 40 (75) | 33 (56) | 27 (42) | |
| | **Total SVI Score** | | | | | | |
| | Mean (SD) | 0.31 (0.05) | 0.42 (0.02) | 0.48 (0.02) | 0.59 (0.04) | 0.69 (0.07) | |
| | Range | 0.00-0.38 | 0.38-0.44 | 0.45-0.52 | 0.52-0.64 | 0.64-0.93 | |
| Ependymomas & Choroid Plexus Tumors | **Sample Size** | 100 | 135 | 84 | 107 | 102 | |
| | **Months Survival** | | | | | | 0.425 |
| | Median (IQR) | 27 (9-56) | 31 (11-63) | 26 (11-46) | 26 (14-52) | 31 (13-63) | |
| | Range | 0-268 | 0-445 | 0-315 | 0-370 | 0-221 | |

© 2023 Fei-Zhang DJ et al. *JAMA Network Open.*

| | | Relative Total SVI %-Tile | | | | | |
|---|---|---|---|---|---|---|---|
| **ICCC Class[1]** | | **<20** | **20-39.99** | **40-59.99** | **60-79.99** | **80-99.99** | **p-value** |
| | Mean (SD) | 41 (48) | 62 (90) | 39 (52) | 43 (61) | 44 (46) | |
| | **Total SVI Score** | | | | | | |
| | Mean (SD) | 0.31 (0.05) | 0.42 (0.02) | 0.48 (0.02) | 0.59 (0.03) | 0.69 (0.06) | |
| | Range | 0.13-0.38 | 0.38-0.44 | 0.45-0.51 | 0.52-0.64 | 0.64-0.93 | |
| Intracranial/spinal Germ Cell Tumors | **Sample Size** | 38 | 39 | 54 | 44 | 38 | |
| | **Months Survival** | | | | | | 0.087 |
| | Median (IQR) | 22 (9-126) | 19 (3-67) | 26 (2-101) | 18 (5-71) | 38 (9-88) | |
| | Range | 0-443 | 0-461 | 0-446 | 0-436 | 0-260 | |
| | Mean (SD) | 103 (143) | 71 (117) | 84 (119) | 64 (104) | 57 (64) | |
| | **Total SVI Score** | | | | | | |
| | Mean (SD) | 0.30 (0.05) | 0.41 (0.02) | 0.48 (0.02) | 0.59 (0.04) | 0.68 (0.06) | |
| | Range | 0.19-0.38 | 0.40-0.44 | 0.45-0.52 | 0.53-0.64 | 0.64-0.93 | |
| Hodgkin Lymphomas | **Sample Size** | 49 | 56 | 34 | 47 | 15 | |
| | **Months Survival** | | | | | | <0.001 |
| | Median (IQR) | 287 (86-374) | 243 (98-322) | 154 (50-301) | 116 (54-232) | 64 (19-135) | |
| | Range | 9-457 | 2-453 | 0-449 | 2-463 | 2-431 | |
| | Mean (SD) | 252 (143) | 218 (131) | 182 (151) | 159 (122) | 113 (133) | |
| | **Total SVI Score** | | | | | | |
| | Mean (SD) | 0.32 (0.05) | 0.42 (0.02) | 0.48 (0.02) | 0.57 (0.02) | 0.73 (0.08) | |
| | Range | 0.19-0.38 | 0.38-0.44 | 0.45-0.52 | 0.52-0.64 | 0.64-0.90 | |
| Non-Hodgkin Lymphomas | **Sample Size** | 33 | 61 | 20 | 42 | 41 | |

© 2023 Fei-Zhang DJ et al. *JAMA Network Open.*

| | | Relative Total SVI %-Tile | | | | | |
|---|---|---|---|---|---|---|---|
| **ICCC Class[1]** | | **<20** | **20-39.99** | **40-59.99** | **60-79.99** | **80-99.99** | **p-value** |
| | **Months Survival** | | | | | | 0.983 |
| | Median (IQR) | 10 (4-35) | 22 (6-52) | 13 (4-71) | 20 (8-68) | 16 (8-40) | |
| | Range | 0-411 | 0-442 | 0-392 | 0-412 | 0-277 | |
| | Mean (SD) | 47 (87) | 59 (102) | 67 (118) | 68 (104) | 44 (69) | |
| | **Total SVI Score** | | | | | | |
| | Mean (SD) | 0.32 (0.05) | 0.42 (0.02) | 0.48 (0.02) | 0.58 (0.03) | 0.71 (0.09) | |
| | Range | 0.20-0.37 | 0.38-0.44 | 0.45-0.52 | 0.52-0.64 | 0.64-0.95 | |
| Malignant Melanomas | **Sample Size** | 74 | 46 | 64 | 47 | 28 | |
| | **Months Survival** | | | | | | 0.302 |
| | Median (IQR) | 41 (19-89) | 49 (19-98) | 66 (23-140) | 35 (17-56) | 42 (18-96) | |
| | Range | 0-360 | 3-365 | 1-380 | 0-251 | 1-169 | |
| | Mean (SD) | 75 (88) | 72 (73) | 101 (97) | 54 (57) | 57 (50) | |
| | **Total SVI Score** | | | | | | |
| | Mean (SD) | 0.30 (0.06) | 0.41 (0.02) | 0.49 (0.02) | 0.59 (0.04) | 0.69 (0.07) | |
| | Range | 0.00-0.38 | 0.38-0.44 | 0.45-0.51 | 0.52-0.64 | 0.64-0.88 | |
| Other-NOS Gliomas | **Sample Size** | 221 | 324 | 260 | 262 | 247 | |
| | **Months Survival** | | | | | | <0.001 |
| | Median (IQR) | 12 (8-25) | 12 (7-23) | 10 (7-20) | 11 (6-20) | 10 (6-16) | |
| | Range | 0-429 | 0-471 | 0-452 | 0-471 | 0-244 | |
| | Mean (SD) | 44 (84) | 42 (86) | 28 (61) | 29 (65) | 17 (28) | |
| | **Total SVI Score** | | | | | | |

© 2023 Fei-Zhang DJ et al. *JAMA Network Open.*

| | | Relative Total SVI %-Tile | | | | | |
|---|---|---|---|---|---|---|---|
| ICCC Class[1] | | <20 | 20-39.99 | 40-59.99 | 60-79.99 | 80-99.99 | p-value |
| | Mean (SD) | 0.31 (0.06) | 0.42 (0.02) | 0.48 (0.02) | 0.58 (0.03) | 0.69 (0.07) | |
| | Range | 0.00-0.38 | 0.38-0.45 | 0.45-0.52 | 0.52-0.64 | 0.64-0.93 | |
| Other or Unspecified Soft Tissue Sarcomas | **Sample Size** | 37 | 42 | 41 | 54 | 37 | |
| | **Months Survival** | | | | | | 0.122 |
| | Median (IQR) | 24 (8-58) | 26 (10-63) | 39 (11-147) | 21 (12-42) | 25 (11-65) | |
| | Range | 0-461 | 0-354 | 0-455 | 0-249 | 0-195 | |
| | Mean (SD) | 64 (113) | 58 (79) | 98 (130) | 40 (52) | 40 (42) | |
| | **Total SVI Score** | | | | | | |
| | Mean (SD) | 0.32 (0.05) | 0.42 (0.02) | 0.49 (0.02) | 0.58 (0.03) | 0.69 (0.07) | |
| | Range | 0.24-0.37 | 0.38-0.44 | 0.45-0.51 | 0.54-0.64 | 0.64-0.90 | |
| Retinoblastomas | **Sample Size** | 21 | 21 | 20 | 30 | 24 | |
| | **Months Survival** | | | | | | 0.153 |
| | Median (IQR) | 30 (23-158) | 33 (15-52) | 64 (32-138) | 20 (11-56) | 16 (10-99) | |
| | Range | 10-468 | 7-423 | 7-287 | 2-334 | 1-301 | |
| | Mean (SD) | 106 (127) | 74 (112) | 98 (94) | 65 (89) | 64 (87) | |
| | **Total SVI Score** | | | | | | |
| | Mean (SD) | 0.29 (0.08) | 0.42 (0.02) | 0.48 (0.02) | 0.58 (0.03) | 0.70 (0.08) | |
| | Range | 0.00-0.37 | 0.40-0.44 | 0.45-0.51 | 0.52-0.64 | 0.64-0.93 | |
| Rhabdomyosarcomas | **Sample Size** | 62 | 76 | 94 | 74 | 77 | |
| | **Months Survival** | | | | | | 0.386 |

© 2023 Fei-Zhang DJ et al. *JAMA Network Open.*

| | | Relative Total SVI %-Tile | | | | | |
|---|---|---|---|---|---|---|---|
| ICCC Class[1] | | <20 | 20-39.99 | 40-59.99 | 60-79.99 | 80-99.99 | p-value |
| | Median (IQR) | 21 (11-34) | 19 (9-40) | 24 (12-37) | 21 (9-35) | 18 (9-33) | |
| | Range | 0-348 | 1-327 | 1-333 | 3-407 | 0-198 | |
| | Mean (SD) | 36 (50) | 39 (58) | 35 (48) | 37 (66) | 29 (36) | |
| | **Total SVI Score** | | | | | | |
| | Mean (SD) | 0.31 (0.04) | 0.41 (0.02) | 0.49 (0.02) | 0.58 (0.03) | 0.70 (0.07) | |
| | Range | 0.23-0.38 | 0.38-0.44 | 0.45-0.52 | 0.52-0.64 | 0.64-0.93 | |
| Thyroid Carcinomas | **Sample Size** | 18 | 16 | 32 | 22 | 20 | |
| | **Months Survival** | | | | | | 0.033 |
| | Median (IQR) | 277 (72-332) | 48 (22-360) | 128 (36-222) | 80 (51-211) | 92 (35-182) | |
| | Range | 9-462 | 2-443 | 0-398 | 1-438 | 4-394 | |
| | Mean (SD) | 218 (149) | 154 (178) | 148 (125) | 134 (130) | 122 (114) | |
| | **Total SVI Score** | | | | | | |
| | Mean (SD) | 0.32 (0.05) | 0.41 (0.02) | 0.48 (0.02) | 0.56 (0.03) | 0.70 (0.09) | |
| | Range | 0.23-0.37 | 0.40-0.44 | 0.45-0.52 | 0.52-0.64 | 0.64-0.93 | |
| [1]By International Classification of Childhood Cancer (ICCC) | | | | | | | |

| | | Relative Socioeconomic-SVI %-Tile | | | | | |
|---|---|---|---|---|---|---|---|
| ICCC Class[1] | | <20 | 20-39.99 | 40-59.99 | 60-79.99 | 80-99.99 | p-value |
| Astrocytomas | **Sample Size** | 430 | 447 | 368 | 366 | 435 | |
| | **Months Survival** | | | | | | <0.001 |
| | Median (IQR) | 18 (7-58) | 14 (7-64) | 16 (7-36) | 12 (7-26) | 15 (7-30) | |
| | Range | 0-466 | 0-473 | 0-456 | 0-368 | 0-472 | |
| | Mean (SD) | 58 (93) | 64 (104) | 49 (86) | 31 (54) | 42 (78) | |

© 2023 Fei-Zhang DJ et al. *JAMA Network Open.*

| | | Relative Socioeconomic-SVI %-Tile | | | | | |
|---|---|---|---|---|---|---|---|
| **ICCC Class[1]** | | **<20** | **20-39.99** | **40-59.99** | **60-79.99** | **80-99.99** | **p-value** |
| | **Socioeconomic SVI Subscore** | | | | | | |
| | Mean (SD) | 0.28 (0.04) | 0.37 (0.02) | 0.44 (0.02) | 0.57 (0.03) | 0.69 (0.07) | |
| | Range | 0.00-0.33 | 0.34-0.41 | 0.41-0.50 | 0.50-0.60 | 0.60-0.94 | |
| Intracranial/spinal Embryonal Tumors | **Sample Size** | 322 | 299 | 279 | 322 | 289 | |
| | **Months Survival** | | | | | | <0.001 |
| | Median (IQR) | 15 (6-41) | 17 (6-54) | 15 (7-38) | 12 (5-29) | 14 (4-32) | |
| | Range | 0-463 | 0-414 | 0-383 | 0-343 | 0-395 | |
| | Mean (SD) | 42 (74) | 52 (83) | 33 (50) | 27 (44) | 32 (56) | |
| | **Socioeconomic SVI Subscore** | | | | | | |
| | Mean (SD) | 0.28 (0.04) | 0.37 (0.02) | 0.45 (0.02) | 0.57 (0.03) | 0.69 (0.07) | |
| | Range | 0.00-0.33 | 0.33-0.41 | 0.41-0.50 | 0.50-0.60 | 0.60-0.94 | |
| Ependymomas & Choroid Plexus Tumors | **Sample Size** | 120 | 102 | 97 | 99 | 110 | |
| | **Months Survival** | | | | | | 0.366 |
| | Median (IQR) | 31 (12-60) | 25 (10-54) | 23 (8-45) | 22 (8-52) | 34 (16-63) | |
| | Range | 0-445 | 0-315 | 0-369 | 0-221 | 0-370 | |
| | Mean (SD) | 56 (80) | 46 (63) | 42 (63) | 37 (44) | 52 (62) | |
| | **Socioeconomic SVI Subscore** | | | | | | |
| | Mean (SD) | 0.28 (0.04) | 0.37 (0.02) | 0.44 (0.02) | 0.58 (0.03) | 0.69 (0.07) | |
| | Range | 0.14-0.33 | 0.34-0.41 | 0.42-0.49 | 0.50-0.60 | 0.61-0.93 | |

© 2023 Fei-Zhang DJ et al. *JAMA Network Open.*

| | | Relative Socioeconomic-SVI %-Tile | | | | | |
|---|---|---|---|---|---|---|---|
| **ICCC Class[1]** | | **<20** | **20-39.99** | **40-59.99** | **60-79.99** | **80-99.99** | **p-value** |
| Intracranial/spinal Germ Cell Tumors | **Sample Size** | 67 | 35 | 33 | 45 | 33 | |
| | **Months Survival** | | | | | | 0.049 |
| | Median (IQR) | 20 (2-107) | 36 (11-211) | 15 (4-38) | 25 (1-81) | 31 (11-85) | |
| | Range | 0-446 | 0-461 | 0-420 | 0-260 | 0-436 | |
| | Mean (SD) | 89 (126) | 117 (145) | 51 (96) | 47 (60) | 71 (106) | |
| | **Socioeconomic SVI Subscore** | | | | | | |
| | Mean (SD) | 0.29 (0.04) | 0.37 (0.02) | 0.45 (0.02) | 0.58 (0.03) | 0.69 (0.07) | |
| | Range | 0.17-0.33 | 0.34-0.41 | 0.42-0.49 | 0.53-0.60 | 0.61-0.90 | |
| Hodgkin Lymphomas | **Sample Size** | 35 | 68 | 44 | 21 | 33 | |
| | **Months Survival** | | | | | | <0.001 |
| | Median (IQR) | 269 (103-367) | 268 (79-344) | 203 (69-292) | 97 (38-237) | 108 (48-199) | |
| | Range | 6-453 | 0-457 | 2-422 | 2-436 | 2-463 | |
| | Mean (SD) | 239 (148) | 231 (142) | 184 (124) | 142 (127) | 144 (134) | |
| | **Socioeconomic SVI Subscore** | | | | | | |
| | Mean (SD) | 0.28 (0.04) | 0.37 (0.02) | 0.44 (0.02) | 0.55 (0.03) | 0.68 (0.06) | |
| | Range | 0.17-0.33 | 0.34-0.41 | 0.41-0.48 | 0.52-0.60 | 0.63-0.88 | |
| Non-Hodgkin Lymphomas | **Sample Size** | 40 | 49 | 27 | 38 | 43 | |
| | **Months Survival** | | | | | | 0.949 |
| | Median (IQR) | 24 (4-49) | 11 (6-67) | 12 (5-62) | 18 (6-68) | 15 (7-47) | |
| | Range | 0-442 | 0-421 | 0-427 | 0-281 | 0-412 | |

© 2023 Fei-Zhang DJ et al. *JAMA Network Open.*

| | | Relative Socioeconomic-SVI %-Tile | | | | | |
|---|---|---|---|---|---|---|---|
| **ICCC Class[1]** | | **<20** | **20-39.99** | **40-59.99** | **60-79.99** | **80-99.99** | **p-value** |
| | Mean (SD) | 50 (83) | 64 (115) | 57 (96) | 53 (75) | 58 (101) | |
| | **Socioeconomic SVI Subscore** | | | | | | |
| | Mean (SD) | 0.28 (0.03) | 0.37 (0.02) | 0.45 (0.02) | 0.57 (0.03) | 0.71 (0.08) | |
| | Range | 0.17-0.33 | 0.34-0.41 | 0.42-0.48 | 0.50-0.60 | 0.60-0.94 | |
| Malignant Melanomas | **Sample Size** | 65 | 63 | 53 | 45 | 33 | |
| | **Months Survival** | | | | | | 0.468 |
| | Median (IQR) | 35 (15-72) | 64 (26-122) | 60 (24-118) | 41 (17-99) | 35 (18-59) | |
| | Range | 0-330 | 3-380 | 1-372 | 0-258 | 1-183 | |
| | Mean (SD) | 58 (65) | 97 (97) | 93 (96) | 64 (65) | 51 (49) | |
| | **Socioeconomic SVI Subscore** | | | | | | |
| | Mean (SD) | 0.27 (0.05) | 0.37 (0.02) | 0.44 (0.02) | 0.56 (0.03) | 0.69 (0.08) | |
| | Range | 0.00-0.33 | 0.33-0.41 | 0.42-0.48 | 0.50-0.60 | 0.60-0.92 | |
| Other-NOS Gliomas | **Sample Size** | 272 | 285 | 251 | 228 | 278 | |
| | **Months Survival** | | | | | | <0.001 |
| | Median (IQR) | 11 (6-22) | 11 (8-24) | 12 (7-22) | 11 (6-18) | 10 (6-17) | |
| | Range | 0-398 | 0-452 | 0-471 | 0-415 | 0-450 | |
| | Mean (SD) | 40 (76) | 40 (82) | 36 (81) | 18 (36) | 25 (54) | |
| | **Socioeconomic SVI Subscore** | | | | | | |
| | Mean (SD) | 0.28 (0.04) | 0.37 (0.02) | 0.44 (0.02) | 0.57 (0.03) | 0.69 (0.07) | |
| | Range | 0.00-0.33 | 0.34-0.41 | 0.41-0.50 | 0.50-0.60 | 0.60-0.93 | |

© 2023 Fei-Zhang DJ et al. *JAMA Network Open.*

| | | Relative Socioeconomic-SVI %-Tile | | | | | |
|---|---|---|---|---|---|---|---|
| **ICCC Class[1]** | | **<20** | **20-39.99** | **40-59.99** | **60-79.99** | **80-99.99** | **p-value** |
| Other or Unspecified Soft Tissue Sarcomas | **Sample Size** | 28 | 52 | 35 | 57 | 39 | |
| | **Months Survival** | | | | | | 0.001 |
| | Median (IQR) | 30 (9-107) | 29 (12-84) | 22 (8-50) | 20 (10-59) | 24 (12-40) | |
| | Range | 0-461 | 0-452 | 0-366 | 0-236 | 0-249 | |
| | Mean (SD) | 94 (138) | 78 (109) | 57 (89) | 39 (44) | 41 (52) | |
| | **Socioeconomic SVI Subscore** | | | | | | |
| | Mean (SD) | 0.28 (0.04) | 0.37 (0.02) | 0.44 (0.02) | 0.56 (0.03) | 0.69 (0.07) | |
| | Range | 0.18-0.33 | 0.34-0.41 | 0.41-0.48 | 0.50-0.60 | 0.61-0.93 | |
| Retinoblastomas | **Sample Size** | 35 | 17 | 14 | 24 | 26 | |
| | **Months Survival** | | | | | | 0.017 |
| | Median (IQR) | 38 (22-155) | 64 (25-129) | 31 (11-48) | 24 (11-99) | 15 (11-38) | |
| | Range | 7-468 | 7-278 | 7-183 | 1-334 | 2-301 | |
| | Mean (SD) | 111 (128) | 93 (92) | 47 (55) | 74 (97) | 51 (80) | |
| | **Socioeconomic SVI Subscore** | | | | | | |
| | Mean (SD) | 0.28 (0.06) | 0.38 (0.02) | 0.45 (0.02) | 0.57 (0.03) | 0.71 (0.09) | |
| | Range | 0.00-0.33 | 0.35-0.41 | 0.41-0.48 | 0.52-0.60 | 0.63-0.93 | |
| Rhabdomyosarcomas | **Sample Size** | 70 | 78 | 87 | 66 | 82 | |
| | **Months Survival** | | | | | | 0.106 |
| | Median (IQR) | 28 (15-53) | 18 (10-34) | 22 (10-38) | 18 (8-31) | 22 (11-35) | |

© 2023 Fei-Zhang DJ et al. *JAMA Network Open.*

| | | Relative Socioeconomic-SVI %-Tile | | | | | |
|---|---|---|---|---|---|---|---|
| ICCC Class[1] | | **<20** | **20-39.99** | **40-59.99** | **60-79.99** | **80-99.99** | **p-value** |
| | Range | 0-204 | 1-348 | 1-407 | 0-345 | 0-274 | |
| | Mean (SD) | 41 (44) | 40 (70) | 34 (54) | 28 (50) | 31 (39) | |
| | **Socioeconomic SVI Subscore** | | | | | | |
| | Mean (SD) | 0.28 (0.04) | 0.37 (0.02) | 0.45 (0.02) | 0.57 (0.03) | 0.70 (0.07) | |
| | Range | 0.17-0.33 | 0.33-0.41 | 0.41-0.49 | 0.50-0.60 | 0.60-0.92 | |
| Thyroid Carcinomas | **Sample Size** | 24 | 24 | 22 | 18 | 20 | |
| | **Months Survival** | | | | | | 0.005 |
| | Median (IQR) | 266 (73-358) | 178 (68-314) | 35 (22-69) | 81 (50-130) | 94 (47-222) | |
| | Range | 2-462 | 1-427 | 0-426 | 1-438 | 8-394 | |
| | Mean (SD) | 227 (155) | 191 (137) | 74 (106) | 111 (111) | 142 (120) | |
| | **Socioeconomic SVI Subscore** | | | | | | |
| | Mean (SD) | 0.29 (0.05) | 0.37 (0.02) | 0.45 (0.02) | 0.57 (0.03) | 0.70 (0.09) | |
| | Range | 0.17-0.33 | 0.34-0.40 | 0.41-0.49 | 0.52-0.60 | 0.64-0.90 | |

[1]By International Classification of Childhood Cancer (ICCC)

| | | Relative Minority-Language-SVI %-Tile | | | | | |
|---|---|---|---|---|---|---|---|
| ICCC Class[1] | | **<20** | **20-39.99** | **40-59.99** | **60-79.99** | **80-99.99** | **p-value** |
| Astrocytomas | **Sample Size** | 438 | 470 | 407 | 341 | 390 | |
| | **Months Survival** | | | | | | <0.001 |
| | Median (IQR) | 16 (7-53) | 16 (7-55) | 14 (7-35) | 15 (8-35) | 12 (7-27) | |
| | Range | 0-456 | 0-472 | 0-473 | 0-425 | 0-472 | |
| | Mean (SD) | 53 (87) | 62 (102) | 47 (84) | 44 (75) | 38 (72) | |

© 2023 Fei-Zhang DJ et al. *JAMA Network Open.*

| | | Relative Minority-Language-SVI %-Tile | | | | | |
|---|---|---|---|---|---|---|---|
| **ICCC Class[1]** | | **<20** | **20-39.99** | **40-59.99** | **60-79.99** | **80-99.99** | **p-value** |
| | **Minority-Language SVI Subscore** | | | | | | |
| | Mean (SD) | 0.29 (0.10) | 0.47 (0.03) | 0.61 (0.05) | 0.76 (0.03) | 0.83 (0.02) | |
| | Range | 0.02-0.42 | 0.42-0.54 | 0.54-0.70 | 0.70-0.80 | 0.80-0.95 | |
| Intracranial/spinal Embryonal Tumors | **Sample Size** | 274 | 309 | 284 | 308 | 336 | |
| | **Months Survival** | | | | | | 0.023 |
| | Median (IQR) | 16 (7-42) | 14 (6-37) | 15 (5-49) | 14 (5-36) | 12 (5-31) | |
| | Range | 0-387 | 0-428 | 0-395 | 0-463 | 0-412 | |
| | Mean (SD) | 41 (64) | 41 (73) | 40 (61) | 36 (69) | 30 (52) | |
| | **Minority-Language SVI Subscore** | | | | | | |
| | Mean (SD) | 0.31 (0.10) | 0.47 (0.04) | 0.61 (0.05) | 0.75 (0.03) | 0.83 (0.02) | |
| | Range | 0.03-0.42 | 0.42-0.54 | 0.54-0.70 | 0.70-0.80 | 0.80-0.95 | |
| Ependymomas & Choroid Plexus Tumors | **Sample Size** | 103 | 107 | 117 | 83 | 118 | |
| | **Months Survival** | | | | | | 0.107 |
| | Median (IQR) | 27 (14-56) | 34 (14-60) | 25 (9-62) | 28 (12-48) | 23 (9-52) | |
| | Range | 0-369 | 0-370 | 0-445 | 0-356 | 0-315 | |
| | Mean (SD) | 47 (60) | 52 (64) | 56 (84) | 43 (60) | 37 (46) | |
| | **Minority-Language SVI Subscore** | | | | | | |
| | Mean (SD) | 0.29 (0.10) | 0.48 (0.03) | 0.60 (0.05) | 0.76 (0.03) | 0.83 (0.02) | |
| | Range | 0.01-0.42 | 0.42-0.54 | 0.54-0.69 | 0.70-0.80 | 0.80-0.95 | |

© 2023 Fei-Zhang DJ et al. *JAMA Network Open.*

| | | Relative Minority-Language-SVI %-Tile | | | | | |
|---|---|---|---|---|---|---|---|
| **ICCC Class[1]** | | **<20** | **20-39.99** | **40-59.99** | **60-79.99** | **80-99.99** | **p-value** |
| Intracranial/spinal Germ Cell Tumors | **Sample Size** | 30 | 41 | 34 | 61 | 47 | |
| | **Months Survival** | | | | | | 0.117 |
| | Median (IQR) | 20 (4-80) | 31 (17-130) | 12 (1-77) | 21 (2-81) | 38 (4-75) | |
| | Range | 0-420 | 0-436 | 0-461 | 0-446 | 0-260 | |
| | Mean (SD) | 88 (134) | 95 (122) | 69 (127) | 80 (117) | 52 (62) | |
| | **Minority-Language SVI Subscore** | | | | | | |
| | Mean (SD) | 0.30 (0.12) | 0.47 (0.03) | 0.62 (0.04) | 0.76 (0.03) | 0.83 (0.02) | |
| | Range | 0.00-0.41 | 0.42-0.51 | 0.55-0.70 | 0.72-0.80 | 0.80-0.84 | |
| Hodgkin Lymphomas | **Sample Size** | 31 | 60 | 66 | 27 | 17 | |
| | **Months Survival** | | | | | | 0.065 |
| | Median (IQR) | 258 (63-332) | 222 (92-314) | 205 (52-332) | 134 (62-281) | 84 (23-299) | |
| | Range | 10-433 | 31-463 | 2-457 | 3-441 | 0-449 | |
| | Mean (SD) | 212 (143) | 216 (130) | 199 (148) | 169 (129) | 155 (161) | |
| | **Minority-Language SVI Subscore** | | | | | | |
| | Mean (SD) | 0.31 (0.09) | 0.47 (0.03) | 0.60 (0.05) | 0.75 (0.03) | 0.83 (0.04) | |
| | Range | 0.05-0.40 | 0.42-0.53 | 0.54-0.69 | 0.72-0.79 | 0.80-0.95 | |
| Non-Hodgkin Lymphomas | **Sample Size** | 30 | 37 | 58 | 28 | 44 | |
| | **Months Survival** | | | | | | 0.267 |
| | Median (IQR) | 10 (5-26) | 14 (2-54) | 20 (8-77) | 8 (3-39) | 29 (14-83) | |
| | Range | 0-248 | 0-412 | 0-442 | 0-185 | 0-392 | |

© 2023 Fei-Zhang DJ et al. *JAMA Network Open.*

| | | Relative Minority-Language-SVI %-Tile | | | | | |
|---|---|---|---|---|---|---|---|
| **ICCC Class[1]** | | **<20** | **20-39.99** | **40-59.99** | **60-79.99** | **80-99.99** | **p-value** |
| | Mean (SD) | 28 (50) | 62 (116) | 71 (111) | 28 (42) | 71 (97) | |
| | **Minority-Language SVI Subscore** | | | | | | |
| | Mean (SD) | 0.32 (0.09) | 0.47 (0.03) | 0.60 (0.05) | 0.76 (0.03) | 0.83 (0.03) | |
| | Range | 0.11-0.42 | 0.42-0.52 | 0.54-0.69 | 0.70-0.79 | 0.80-0.95 | |
| Malignant Melanomas | **Sample Size** | 60 | 55 | 52 | 53 | 39 | |
| | **Months Survival** | | | | | | 0.511 |
| | Median (IQR) | 40 (17-82) | 61 (24-152) | 43 (17-104) | 40 (19-81) | 45 (19-122) | |
| | Range | 0-360 | 0-372 | 0-365 | 1-380 | 2-352 | |
| | Mean (SD) | 67 (78) | 102 (98) | 70 (74) | 62 (70) | 74 (74) | |
| | **Minority-Language SVI Subscore** | | | | | | |
| | Mean (SD) | 0.30 (0.09) | 0.47 (0.04) | 0.61 (0.04) | 0.75 (0.03) | 0.83 (0.02) | |
| | Range | 0.07-0.41 | 0.42-0.53 | 0.54-0.68 | 0.72-0.80 | 0.80-0.84 | |
| Other-NOS Gliomas | **Sample Size** | 244 | 276 | 290 | 258 | 246 | |
| | **Months Survival** | | | | | | <0.001 |
| | Median (IQR) | 11 (7-21) | 12 (7-24) | 12 (7-22) | 10 (6-17) | 10 (6-18) | |
| | Range | 0-471 | 0-450 | 0-471 | 0-452 | 0-324 | |
| | Mean (SD) | 42 (84) | 41 (79) | 36 (79) | 23 (53) | 19 (35) | |
| | **Minority-Language SVI Subscore** | | | | | | |
| | Mean (SD) | 0.29 (0.11) | 0.47 (0.03) | 0.60 (0.04) | 0.76 (0.03) | 0.83 (0.02) | |
| | Range | 0.03-0.42 | 0.42-0.54 | 0.54-0.70 | 0.70-0.80 | 0.80-0.95 | |

© 2023 Fei-Zhang DJ et al. *JAMA Network Open.*

| | | Relative Minority-Language-SVI %-Tile | | | | | |
|---|---|---|---|---|---|---|---|
| **ICCC Class[1]** | | **<20** | **20-39.99** | **40-59.99** | **60-79.99** | **80-99.99** | **p-value** |
| Other or Unspecified Soft Tissue Sarcomas | **Sample Size** | 37 | 40 | 51 | 38 | 45 | |
| | **Months Survival** | | | | | | 0.361 |
| | Median (IQR) | 26 (10-49) | 29 (8-68) | 22 (10-54) | 23 (16-62) | 25 (11-79) | |
| | Range | 0-341 | 0-461 | 0-285 | 0-455 | 0-449 | |
| | Mean (SD) | 42 (63) | 73 (114) | 43 (56) | 80 (118) | 61 (86) | |
| | **Minority-Language SVI Subscore** | | | | | | |
| | Mean (SD) | 0.29 (0.10) | 0.48 (0.03) | 0.62 (0.04) | 0.76 (0.03) | 0.83 (0.02) | |
| | Range | 0.06-0.41 | 0.42-0.53 | 0.54-0.68 | 0.72-0.80 | 0.80-0.95 | |
| Retinoblastomas | **Sample Size** | 16 | 24 | 28 | 23 | 25 | |
| | **Months Survival** | | | | | | 0.667 |
| | Median (IQR) | 31 (14-175) | 19 (13-46) | 32 (15-103) | 44 (15-126) | 25 (16-85) | |
| | Range | 9-468 | 2-263 | 2-423 | 7-301 | 1-278 | |
| | Mean (SD) | 108 (143) | 53 (71) | 88 (117) | 89 (96) | 68 (80) | |
| | **Minority-Language SVI Subscore** | | | | | | |
| | Mean (SD) | 0.25 (0.12) | 0.47 (0.03) | 0.62 (0.05) | 0.76 (0.03) | 0.83 (0.02) | |
| | Range | 0.06-0.41 | 0.42-0.51 | 0.54-0.68 | 0.70-0.79 | 0.80-0.84 | |
| Rhabdomyosarcomas | **Sample Size** | 83 | 81 | 61 | 87 | 71 | |
| | **Months Survival** | | | | | | 0.679 |
| | Median (IQR) | 18 (10-32) | 20 (12-35) | 26 (9-36) | 31 (16-44) | 16 (9-26) | |

© 2023 Fei-Zhang DJ et al. *JAMA Network Open.*

| | | Relative Minority-Language-SVI %-Tile | | | | | |
|---|---|---|---|---|---|---|---|
| ICCC Class[1] | | <20 | 20-39.99 | 40-59.99 | 60-79.99 | 80-99.99 | p-value |
| | Range | 0-327 | 2-407 | 0-345 | 3-204 | 0-333 | |
| | Mean (SD) | 34 (58) | 37 (64) | 36 (51) | 37 (35) | 30 (50) | |
| | **Minority-Language SVI Subscore** | | | | | | |
| | Mean (SD) | 0.30 (0.10) | 0.48 (0.04) | 0.61 (0.04) | 0.75 (0.03) | 0.83 (0.02) | |
| | Range | 0.03-0.42 | 0.42-0.54 | 0.54-0.70 | 0.70-0.80 | 0.80-0.95 | |
| Thyroid Carcinomas | **Sample Size** | 12 | 28 | 20 | 24 | 24 | |
| | **Months Survival** | | | | | | 0.408 |
| | Median (IQR) | 134 (47-305) | 68 (24-255) | 108 (59-318) | 138 (41-300) | 92 (42-180) | |
| | Range | 9-462 | 5-426 | 0-443 | 1-398 | 4-394 | |
| | Mean (SD) | 188 (161) | 136 (135) | 174 (161) | 170 (140) | 119 (107) | |
| | **Minority-Language SVI Subscore** | | | | | | |
| | Mean (SD) | 0.28 (0.13) | 0.48 (0.03) | 0.61 (0.05) | 0.76 (0.03) | 0.83 (0.03) | |
| | Range | 0.00-0.41 | 0.42-0.51 | 0.55-0.67 | 0.72-0.79 | 0.80-0.95 | |
| [1]By International Classification of Childhood Cancer (ICCC) | | | | | | | |

| | | Relative Household Composition-SVI %-Tile | | | | | |
|---|---|---|---|---|---|---|---|
| ICCC Class[1] | | <20 | 20-39.99 | 40-59.99 | 60-79.99 | 80-99.99 | p-value |
| Astrocytomas | **Sample Size** | 398 | 412 | 364 | 389 | 483 | |
| | **Months Survival** | | | | | | 0.989 |
| | Median (IQR) | 17 (8-45) | 14 (6-40) | 14 (8-36) | 14 (7-28) | 15 (7-46) | |
| | Range | 0-472 | 0-443 | 0-473 | 0-456 | 0-472 | |
| | Mean (SD) | 51 (85) | 51 (89) | 47 (80) | 42 (79) | 55 (93) | |

© 2023 Fei-Zhang DJ et al. *JAMA Network Open.*

| | | Relative Household Composition-SVI %-Tile | | | | | |
|---|---|---|---|---|---|---|---|
| **ICCC Class[1]** | | **<20** | **20-39.99** | **40-59.99** | **60-79.99** | **80-99.99** | **p-value** |
| | **Household Composition SVI Subscore** | | | | | | |
| | Mean (SD) | 0.25 (0.04) | 0.35 (0.01) | 0.40 (0.03) | 0.51 (0.03) | 0.68 (0.07) | |
| | Range | 0.15-0.33 | 0.33-0.36 | 0.36-0.46 | 0.46-0.58 | 0.58-0.95 | |
| Intracranial/spinal Embryonal Tumors | **Sample Size** | 295 | 343 | 293 | 289 | 291 | |
| | **Months Survival** | | | | | | 0.019 |
| | Median (IQR) | 16 (6-50) | 14 (6-38) | 13 (5-39) | 13 (6-33) | 15 (6-38) | |
| | Range | 0-463 | 0-428 | 0-345 | 0-355 | 0-395 | |
| | Mean (SD) | 49 (84) | 34 (59) | 35 (60) | 34 (55) | 35 (57) | |
| | **Household Composition SVI Subscore** | | | | | | |
| | Mean (SD) | 0.25 (0.04) | 0.35 (0.01) | 0.40 (0.03) | 0.51 (0.03) | 0.68 (0.07) | |
| | Range | 0.15-0.33 | 0.33-0.36 | 0.36-0.46 | 0.46-0.58 | 0.58-0.94 | |
| Ependymomas & Choroid Plexus Tumors | **Sample Size** | 99 | 129 | 90 | 100 | 110 | |
| | **Months Survival** | | | | | | 0.700 |
| | Median (IQR) | 29 (8-56) | 22 (10-52) | 28 (9-60) | 24 (13-50) | 34 (14-67) | |
| | Range | 0-430 | 0-445 | 0-347 | 0-369 | 0-370 | |
| | Mean (SD) | 52 (81) | 38 (51) | 52 (70) | 42 (55) | 52 (64) | |
| | **Household Composition SVI Subscore** | | | | | | |
| | Mean (SD) | 0.25 (0.04) | 0.35 (0.01) | 0.40 (0.03) | 0.50 (0.03) | 0.67 (0.06) | |
| | Range | 0.15-0.33 | 0.33-0.36 | 0.36-0.46 | 0.46-0.58 | 0.59-0.87 | |

© 2023 Fei-Zhang DJ et al. *JAMA Network Open.*

| | | Relative Household Composition-SVI %-Tile | | | | | |
|---|---|---|---|---|---|---|---|
| **ICCC Class[1]** | | **<20** | **20-39.99** | **40-59.99** | **60-79.99** | **80-99.99** | **p-value** |
| Intracranial/spinal Germ Cell Tumors | **Sample Size** | 54 | 52 | 38 | 30 | 39 | |
| | **Months Survival** | | | | | | 0.768 |
| | Median (IQR) | 18 (2-79) | 36 (10-81) | 30 (4-120) | 14 (3-42) | 29 (6-109) | |
| | Range | 0-446 | 0-403 | 0-461 | 0-420 | 0-436 | |
| | Mean (SD) | 83 (128) | 71 (101) | 84 (113) | 53 (104) | 82 (115) | |
| | **Household Composition SVI Subscore** | | | | | | |
| | Mean (SD) | 0.23 (0.05) | 0.35 (0.01) | 0.40 (0.03) | 0.51 (0.03) | 0.66 (0.07) | |
| | Range | 0.15-0.33 | 0.34-0.36 | 0.36-0.46 | 0.46-0.58 | 0.58-0.83 | |
| Hodgkin Lymphomas | **Sample Size** | 35 | 37 | 49 | 37 | 43 | |
| | **Months Survival** | | | | | | 0.025 |
| | Median (IQR) | 187 (62-335) | 287 (79-384) | 242 (97-306) | 112 (52-296) | 126 (50-258) | |
| | Range | 0-449 | 6-457 | 2-441 | 2-436 | 2-463 | |
| | Mean (SD) | 206 (152) | 243 (157) | 208 (120) | 176 (136) | 162 (137) | |
| | **Household Composition SVI Subscore** | | | | | | |
| | Mean (SD) | 0.26 (0.05) | 0.34 (0.01) | 0.41 (0.03) | 0.50 (0.03) | 0.66 (0.06) | |
| | Range | 0.15-0.33 | 0.33-0.36 | 0.36-0.46 | 0.46-0.57 | 0.59-0.84 | |
| Non-Hodgkin Lymphomas | **Sample Size** | 43 | 37 | 45 | 32 | 40 | |
| | **Months Survival** | | | | | | 0.359 |
| | Median (IQR) | 25 (10-69) | 13 (4-78) | 17 (6-68) | 12 (4-35) | 14 (8-44) | |
| | Range | 0-442 | 0-411 | 0-427 | 0-248 | 0-412 | |

© 2023 Fei-Zhang DJ et al. *JAMA Network Open.*

| | | Relative Household Composition-SVI %-Tile | | | | | |
|---|---|---|---|---|---|---|---|
| **ICCC Class[1]** | | **<20** | **20-39.99** | **40-59.99** | **60-79.99** | **80-99.99** | **p-value** |
| | Mean (SD) | 67 (108) | 52 (84) | 71 (111) | 30 (48) | 55 (99) | |
| | **Household Composition SVI Subscore** | | | | | | |
| | Mean (SD) | 0.25 (0.03) | 0.35 (0.01) | 0.41 (0.03) | 0.50 (0.03) | 0.69 (0.08) | |
| | Range | 0.22-0.33 | 0.33-0.36 | 0.36-0.46 | 0.46-0.57 | 0.59-0.91 | |
| Malignant Melanomas | **Sample Size** | 51 | 59 | 53 | 58 | 38 | |
| | **Months Survival** | | | | | | 0.758 |
| | Median (IQR) | 44 (15-92) | 44 (20-116) | 63 (30-109) | 35 (18-94) | 45 (20-89) | |
| | Range | 0-360 | 0-333 | 1-380 | 1-372 | 0-237 | |
| | Mean (SD) | 68 (79) | 77 (80) | 90 (93) | 72 (83) | 63 (62) | |
| | **Household Composition SVI Subscore** | | | | | | |
| | Mean (SD) | 0.26 (0.04) | 0.35 (0.01) | 0.39 (0.03) | 0.51 (0.03) | 0.67 (0.06) | |
| | Range | 0.15-0.33 | 0.33-0.36 | 0.36-0.44 | 0.46-0.58 | 0.59-0.84 | |
| Other-NOS Gliomas | **Sample Size** | 264 | 233 | 287 | 265 | 265 | |
| | **Months Survival** | | | | | | 0.799 |
| | Median (IQR) | 11 (7-20) | 11 (6-25) | 11 (7-18) | 12 (7-20) | 11 (6-19) | |
| | Range | 0-347 | 0-429 | 0-471 | 0-471 | 0-471 | |
| | Mean (SD) | 27 (54) | 40 (79) | 31 (74) | 32 (69) | 33 (70) | |
| | **Household Composition SVI Subscore** | | | | | | |
| | Mean (SD) | 0.25 (0.04) | 0.35 (0.01) | 0.40 (0.03) | 0.50 (0.03) | 0.68 (0.07) | |
| | Range | 0.15-0.33 | 0.33-0.36 | 0.36-0.46 | 0.46-0.58 | 0.58-0.91 | |

© 2023 Fei-Zhang DJ et al. *JAMA Network Open.*

| | | Relative Household Composition-SVI %-Tile | | | | | |
|---|---|---|---|---|---|---|---|
| ICCC Class[1] | | <20 | 20-39.99 | 40-59.99 | 60-79.99 | 80-99.99 | p-value |
| Other or Unspecified Soft Tissue Sarcomas | **Sample Size** | 38 | 39 | 42 | 52 | 40 | |
| | **Months Survival** | | | | | | 0.025 |
| | Median (IQR) | 22 (6-74) | 26 (11-80) | 31 (16-60) | 24 (11-61) | 21 (8-39) | |
| | Range | 0-449 | 0-461 | 5-285 | 0-366 | 0-249 | |
| | Mean (SD) | 68 (103) | 89 (134) | 52 (62) | 55 (78) | 35 (46) | |
| | **Household Composition SVI Subscore** | | | | | | |
| | Mean (SD) | 0.27 (0.03) | 0.35 (0.01) | 0.40 (0.03) | 0.51 (0.03) | 0.66 (0.05) | |
| | Range | 0.21-0.33 | 0.33-0.36 | 0.36-0.45 | 0.46-0.58 | 0.58-0.80 | |
| Retinoblastomas | **Sample Size** | 24 | 21 | 28 | 18 | 25 | |
| | **Months Survival** | | | | | | 0.020 |
| | Median (IQR) | 44 (26-192) | 44 (22-112) | 39 (15-144) | 22 (14-38) | 15 (10-43) | |
| | Range | 7-423 | 10-287 | 1-334 | 2-468 | 2-301 | |
| | Mean (SD) | 116 (125) | 81 (87) | 85 (94) | 65 (115) | 48 (79) | |
| | **Household Composition SVI Subscore** | | | | | | |
| | Mean (SD) | 0.24 (0.05) | 0.35 (0.01) | 0.40 (0.03) | 0.52 (0.03) | 0.69 (0.07) | |
| | Range | 0.15-0.32 | 0.33-0.36 | 0.36-0.45 | 0.47-0.58 | 0.61-0.90 | |
| Rhabdomyosarcomas | **Sample Size** | 66 | 66 | 80 | 79 | 92 | |
| | **Months Survival** | | | | | | 0.424 |
| | Median (IQR) | 24 (12-42) | 23 (12-45) | 20 (9-34) | 22 (12-40) | 18 (10-32) | |

© 2023 Fei-Zhang DJ et al. *JAMA Network Open.*

| | | Relative Household Composition-SVI %-Tile | | | | | |
|---|---|---|---|---|---|---|---|
| **ICCC Class[1]** | | **<20** | **20-39.99** | **40-59.99** | **60-79.99** | **80-99.99** | **p-value** |
| | Range | 0-333 | 0-348 | 0-288 | 1-345 | 0-407 | |
| | Mean (SD) | 39 (53) | 37 (48) | 31 (42) | 41 (63) | 29 (53) | |
| | **Household Composition SVI Subscore** | | | | | | |
| | Mean (SD) | 0.25 (0.04) | 0.35 (0.01) | 0.40 (0.03) | 0.50 (0.03) | 0.68 (0.07) | |
| | Range | 0.15-0.33 | 0.33-0.36 | 0.36-0.46 | 0.46-0.58 | 0.59-0.88 | |
| Thyroid Carcinomas | **Sample Size** | 18 | 28 | 27 | 16 | 19 | |
| | **Months Survival** | | | | | | 0.149 |
| | Median (IQR) | 153 (43-269) | 151 (69-311) | 58 (19-180) | 36 (22-113) | 100 (60-232) | |
| | Range | 2-462 | 20-413 | 1-427 | 0-438 | 10-394 | |
| | Mean (SD) | 178 (154) | 190 (139) | 126 (139) | 107 (135) | 150 (115) | |
| | **Household Composition SVI Subscore** | | | | | | |
| | Mean (SD) | 0.25 (0.04) | 0.35 (0.01) | 0.41 (0.03) | 0.51 (0.03) | 0.68 (0.08) | |
| | Range | 0.15-0.32 | 0.33-0.36 | 0.36-0.46 | 0.48-0.58 | 0.60-0.89 | |

[1]By International Classification of Childhood Cancer (ICCC)

| | | Relative Housing-Transportation-SVI %-Tile | | | | | |
|---|---|---|---|---|---|---|---|
| **ICCC Class[1]** | | **<20** | **20-39.99** | **40-59.99** | **60-79.99** | **80-99.99** | **p-value** |
| Astrocytomas | **Sample Size** | 427 | 436 | 407 | 392 | 384 | |
| | **Months Survival** | | | | | | <0.001 |
| | Median (IQR) | 16 (7-53) | 17 (8-48) | 14 (7-31) | 15 (8-37) | 14 (7-30) | |
| | Range | 0-456 | 0-473 | 0-443 | 0-472 | 0-425 | |
| | Mean (SD) | 58 (95) | 61 (100) | 44 (82) | 44 (77) | 39 (68) | |

© 2023 Fei-Zhang DJ et al. *JAMA Network Open.*

| | | Relative Housing-Transportation-SVI %-Tile | | | | | |
|---|---|---|---|---|---|---|---|
| **ICCC Class[1]** | | **<20** | **20-39.99** | **40-59.99** | **60-79.99** | **80-99.99** | **p-value** |
| | **Housing-Transportation SVI Subscore** | | | | | | |
| | Mean (SD) | 0.35 (0.04) | 0.44 (0.02) | 0.53 (0.02) | 0.58 (0.02) | 0.65 (0.06) | |
| | Range | 0.05-0.40 | 0.40-0.49 | 0.49-0.55 | 0.55-0.62 | 0.62-0.89 | |
| Intracranial/spinal Embryonal Tumors | **Sample Size** | 283 | 294 | 308 | 295 | 331 | |
| | **Months Survival** | | | | | | 0.036 |
| | Median (IQR) | 17 (6-44) | 13 (5-42) | 14 (5-32) | 16 (7-42) | 13 (5-33) | |
| | Range | 0-414 | 0-428 | 0-430 | 0-412 | 0-463 | |
| | Mean (SD) | 42 (65) | 43 (78) | 32 (59) | 37 (57) | 33 (60) | |
| | **Housing-Transportation SVI Subscore** | | | | | | |
| | Mean (SD) | 0.34 (0.04) | 0.44 (0.02) | 0.53 (0.02) | 0.58 (0.02) | 0.65 (0.05) | |
| | Range | 0.18-0.40 | 0.40-0.48 | 0.49-0.55 | 0.55-0.62 | 0.62-0.84 | |
| Ependymomas & Choroid Plexus Tumors | **Sample Size** | 102 | 117 | 103 | 101 | 105 | |
| | **Months Survival** | | | | | | 0.338 |
| | Median (IQR) | 27 (9-57) | 29 (10-59) | 25 (13-54) | 29 (13-57) | 27 (12-54) | |
| | Range | 0-369 | 0-370 | 0-445 | 0-430 | 0-221 | |
| | Mean (SD) | 45 (61) | 56 (78) | 44 (58) | 51 (75) | 39 (39) | |
| | **Housing-Transportation SVI Subscore** | | | | | | |
| | Mean (SD) | 0.35 (0.04) | 0.44 (0.02) | 0.52 (0.02) | 0.58 (0.02) | 0.65 (0.05) | |

© 2023 Fei-Zhang DJ et al. *JAMA Network Open.*

| | | Relative Housing-Transportation-SVI %-Tile | | | | | |
|---|---|---|---|---|---|---|---|
| **ICCC Class[1]** | | **<20** | **20-39.99** | **40-59.99** | **60-79.99** | **80-99.99** | **p-value** |
| | Range | 0.24-0.40 | 0.41-0.48 | 0.49-0.55 | 0.55-0.61 | 0.62-0.88 | |
| Intracranial/spinal Germ Cell Tumors | **Sample Size** | 40 | 36 | 46 | 41 | 50 | |
| | **Months Survival** | | | | | | 0.323 |
| | Median (IQR) | 22 (8-116) | 16 (4-113) | 20 (1-53) | 16 (2-64) | 50 (11-116) | |
| | Range | 0-443 | 0-461 | 0-371 | 0-301 | 0-446 | |
| | Mean (SD) | 102 (145) | 85 (128) | 58 (101) | 53 (76) | 86 (104) | |
| | **Housing-Transportation SVI Subscore** | | | | | | |
| | Mean (SD) | 0.33 (0.06) | 0.45 (0.02) | 0.53 (0.02) | 0.58 (0.02) | 0.65 (0.05) | |
| | Range | 0.18-0.40 | 0.40-0.48 | 0.49-0.55 | 0.55-0.61 | 0.62-0.81 | |
| Hodgkin Lymphomas | **Sample Size** | 53 | 73 | 27 | 23 | 25 | |
| | **Months Survival** | | | | | | 0.013 |
| | Median (IQR) | 282 (79-363) | 199 (72-271) | 180 (56-300) | 201 (36-372) | 116 (64-224) | |
| | Range | 9-457 | 2-463 | 2-453 | 0-449 | 15-342 | |
| | Mean (SD) | 236 (149) | 194 (127) | 188 (145) | 205 (174) | 137 (102) | |
| | **Housing-Transportation SVI Subscore** | | | | | | |
| | Mean (SD) | 0.36 (0.04) | 0.45 (0.02) | 0.52 (0.02) | 0.59 (0.02) | 0.65 (0.03) | |
| | Range | 0.18-0.40 | 0.40-0.48 | 0.49-0.55 | 0.55-0.60 | 0.62-0.73 | |
| Non-Hodgkin Lymphomas | **Sample Size** | 39 | 42 | 47 | 29 | 40 | |
| | **Months Survival** | | | | | | 0.980 |

© 2023 Fei-Zhang DJ et al. *JAMA Network Open.*

| | | Relative Housing-Transportation-SVI %-Tile | | | | | |
|---|---|---|---|---|---|---|---|
| **ICCC Class[1]** | | **<20** | **20-39.99** | **40-59.99** | **60-79.99** | **80-99.99** | **p-value** |
| | Median (IQR) | 12 (4-67) | 13 (5-74) | 15 (4-40) | 33 (7-69) | 18 (8-61) | |
| | Range | 0-411 | 0-427 | 0-281 | 0-442 | 0-277 | |
| | Mean (SD) | 52 (87) | 72 (125) | 38 (61) | 79 (124) | 51 (73) | |
| | **Housing-Transportation SVI Subscore** | | | | | | |
| | Mean (SD) | 0.35 (0.04) | 0.45 (0.02) | 0.53 (0.02) | 0.59 (0.02) | 0.65 (0.07) | |
| | Range | 0.28-0.40 | 0.40-0.48 | 0.50-0.55 | 0.55-0.60 | 0.62-0.89 | |
| Malignant Melanomas | **Sample Size** | 77 | 55 | 41 | 47 | 39 | |
| | **Months Survival** | | | | | | 0.130 |
| | Median (IQR) | 45 (19-106) | 45 (26-97) | 43 (19-118) | 44 (18-114) | 44 (19-96) | |
| | Range | 0-360 | 0-380 | 1-372 | 1-352 | 0-169 | |
| | Mean (SD) | 81 (90) | 79 (85) | 83 (93) | 69 (70) | 56 (47) | |
| | **Housing-Transportation SVI Subscore** | | | | | | |
| | Mean (SD) | 0.35 (0.04) | 0.45 (0.02) | 0.52 (0.02) | 0.58 (0.02) | 0.65 (0.05) | |
| | Range | 0.23-0.40 | 0.41-0.48 | 0.49-0.55 | 0.55-0.62 | 0.62-0.81 | |
| Other-NOS Gliomas | **Sample Size** | 213 | 301 | 293 | 261 | 246 | |
| | **Months Survival** | | | | | | <0.001 |
| | Median (IQR) | 13 (8-23) | 12 (7-21) | 11 (6-20) | 11 (7-20) | 10 (6-18) | |
| | Range | 0-471 | 0-471 | 0-471 | 0-324 | 0-290 | |
| | Mean (SD) | 45 (87) | 40 (86) | 34 (72) | 25 (47) | 19 (34) | |

© 2023 Fei-Zhang DJ et al. *JAMA Network Open.*

| | | Relative Housing-Transportation-SVI %-Tile | | | | | |
|---|---|---|---|---|---|---|---|
| **ICCC Class[1]** | | **<20** | **20-39.99** | **40-59.99** | **60-79.99** | **80-99.99** | **p-value** |
| | **Housing-Transportation SVI Subscore** | | | | | | |
| | Mean (SD) | 0.34 (0.04) | 0.45 (0.02) | 0.52 (0.02) | 0.58 (0.02) | 0.65 (0.05) | |
| | Range | 0.21-0.40 | 0.40-0.48 | 0.49-0.55 | 0.55-0.62 | 0.62-0.89 | |
| Other or Unspecified Soft Tissue Sarcomas | **Sample Size** | 44 | 45 | 50 | 39 | 33 | |
| | **Months Survival** | | | | | | 0.274 |
| | Median (IQR) | 23 (8-51) | 26 (7-81) | 33 (20-82) | 20 (11-57) | 25 (10-56) | |
| | Range | 0-461 | 0-285 | 3-455 | 0-449 | 0-236 | |
| | Mean (SD) | 65 (116) | 57 (73) | 74 (102) | 52 (85) | 41 (48) | |
| | **Housing-Transportation SVI Subscore** | | | | | | |
| | Mean (SD) | 0.35 (0.04) | 0.45 (0.02) | 0.52 (0.02) | 0.58 (0.02) | 0.63 (0.03) | |
| | Range | 0.28-0.40 | 0.41-0.48 | 0.49-0.55 | 0.55-0.62 | 0.62-0.73 | |
| Retinoblastomas | **Sample Size** | 16 | 24 | 24 | 20 | 32 | |
| | **Months Survival** | | | | | | 0.649 |
| | Median (IQR) | 41 (23-174) | 29 (15-64) | 18 (10-54) | 36 (17-154) | 24 (12-150) | |
| | Range | 10-333 | 5-219 | 2-334 | 7-468 | 1-301 | |
| | Mean (SD) | 101 (106) | 56 (66) | 56 (87) | 116 (147) | 81 (94) | |
| | **Housing-Transportation SVI Subscore** | | | | | | |
| | Mean (SD) | 0.33 (0.04) | 0.44 (0.02) | 0.52 (0.02) | 0.59 (0.02) | 0.66 (0.05) | |

© 2023 Fei-Zhang DJ et al. *JAMA Network Open.*

| | | Relative Housing-Transportation-SVI %-Tile | | | | | |
|---|---|---|---|---|---|---|---|
| **ICCC Class[1]** | | **<20** | **20-39.99** | **40-59.99** | **60-79.99** | **80-99.99** | **p-value** |
| | Range | 0.24-0.40 | 0.42-0.48 | 0.49-0.55 | 0.55-0.61 | 0.62-0.81 | |
| Rhabdomyosarcomas | **Sample Size** | 63 | 74 | 77 | 94 | 75 | |
| | **Months Survival** | | | | | | 0.455 |
| | Median (IQR) | 17 (11-36) | 20 (11-34) | 22 (12-40) | 24 (11-39) | 18 (9-30) | |
| | Range | 1-348 | 0-345 | 2-407 | 1-333 | 0-288 | |
| | Mean (SD) | 37 (58) | 32 (51) | 43 (65) | 34 (43) | 29 (45) | |
| | **Housing-Transportation SVI Subscore** | | | | | | |
| | Mean (SD) | 0.35 (0.04) | 0.45 (0.02) | 0.53 (0.02) | 0.58 (0.02) | 0.65 (0.05) | |
| | Range | 0.25-0.40 | 0.40-0.48 | 0.49-0.55 | 0.55-0.62 | 0.62-0.82 | |
| Thyroid Carcinomas | **Sample Size** | 17 | 29 | 24 | 16 | 22 | |
| | **Months Survival** | | | | | | 0.501 |
| | Median (IQR) | 85 (53-306) | 126 (57-297) | 58 (24-222) | 87 (34-194) | 123 (61-203) | |
| | Range | 5-462 | 0-438 | 1-398 | 2-443 | 4-426 | |
| | Mean (SD) | 172 (158) | 173 (141) | 125 (136) | 127 (125) | 160 (135) | |
| | **Housing-Transportation SVI Subscore** | | | | | | |
| | Mean (SD) | 0.36 (0.04) | 0.45 (0.02) | 0.53 (0.02) | 0.59 (0.02) | 0.68 (0.07) | |
| | Range | 0.30-0.40 | 0.40-0.48 | 0.49-0.55 | 0.55-0.60 | 0.62-0.84 | |

[1]By International Classification of Childhood Cancer (ICCC)

© 2023 Fei-Zhang DJ et al. *JAMA Network Open.*
