# Supplementary material for: Assessment of Social Vulnerability in Pediatric Head and Neck Cancer Care and Prognosis in the United States
Source: JAMA Netw Open. 2023 Feb 17;6(2):e230016. doi: 10.1001/jamanetworkopen.2023.0016 (PMC9938432; doi:10.1001/jamanetworkopen.2023.0016)
Supplement: Supplement 2. — Data Sharing Statement [file jamanetwopen-e230016-s002.pdf]

Data Sharing Statement

Fei-Zhang. Assessment of Social Vulnerability in Pediatric Head and Neck Cancer Care and Prognosis in the United States. *JAMA Netw Open*. Published February 17, 2023. doi:10.1001/jamanetworkopen.2023.0016

Data
Data available: No

Additional Information
Explanation for why data not available: Due to designated data-sharing agreements with the Surveillance, Epidemiology, and End-Results (SEER) datasets, we will be unable to provide this data readily.
